# Systematic Investigation of Tumor Immune Microenvironment Modulation by *Cynomorium songaricum* Against Breast Cancer Through Integrated Chemomics, Network Pharmacology and Molecular Docking

**DOI:** 10.3390/ph19020314

**Published:** 2026-02-13

**Authors:** Ze-An Mao, Mei-Ling Zhang, Zi-Yi An, Wei-Lin Jin

**Affiliations:** 1 Institute of Cancer Neuroscience, Medical Frontier Innovation Research Center, The First Hospital of Lanzhou University, The First Clinical Medical College of Lanzhou University, Lanzhou 730000, China; 320220927421@lzu.edu.cn (Z.-A.M.); 320220912160@lzu.edu.cn (M.-L.Z.); 120220903500@lzu.edu.cn (Z.-Y.A.); 2 School of Life Science, Lanzhou University, Lanzhou 730000, China

**Keywords:** *Cynomorium songaricum* Rupr., breast cancer, UHPLC-Q-Exactive Orbitrap MS/MS, network pharmacology, tumor immune microenvironment, multi-target therapy

## Abstract

**Background/Objectives**: Breast cancer remains a leading cause of cancer-related mortality in women, with therapeutic resistance frequently arising from tumor heterogeneity and an immunosuppressive tumor immune microenvironment (TIME). While *Cynomorium songaricum* Rupr. (CS) has been used traditionally in Chinese medicine and exhibits preliminary anti-tumor activity, its bioactive constituents and precise mechanisms against breast cancer remain to be elucidated. **Methods**: The chemical constituents of CS were systematically profiled using ultra-high-performance liquid chromatography coupled with Q Exactive Orbitrap mass spectrometry (UHPLC-Q-Exactive Orbitrap MS/MS). Network pharmacology and functional enrichment analyses were performed to identify immuno-related targets and pathways, followed by molecular docking to prioritize component–target pairs. Molecular dynamics (MD) simulations were conducted to validate the stability of a representative docked complex and to characterize binding stability, interaction persistence, molecular mechanics/(Poisson–Boltzmann) surface area (MM/(P)BSA) energetics, and principal component analysis (PCA)-based conformational landscapes. **Results**: We identified 1100 compounds, of which 84 satisfied the in silico drug-likeness criteria, including 12 phenylpropanoids, 4 terpenes, 35 flavonoids, 2 quinones, 1 phenol, 3 alkaloids, and other phytochemicals. Network pharmacology analysis revealed 776 overlapping targets associated with both breast cancer and immune regulation. Functional enrichment analysis underscored significant involvement in immune-related pathways, and molecular docking studies supported high-affinity interactions between the components and their targets. MD analyses further supported a stable bound ensemble for the representative SRC–Tomentogenin complex during the equilibrated window, with persistent pocket occupancy, consistent interaction signatures, favorable MM/(P)BSA binding energetics, and a concentrated low-energy basin on the PCA-based free energy landscape. **Conclusions**: These findings elucidate the chemical basis of CS and uncover its immunomodulatory mechanism against breast cancer, offering a foundation for developing CS-based immunotherapeutic strategies and supporting multi-target drug discovery from traditional medicines.

## 1. Introduction

Breast cancer is the most frequently diagnosed malignancy and a leading cause of cancer-related death among women globally [1,2]. Despite remarkable progress in endocrine therapies, HER2-targeted agents, and *CDK4/6* inhibitors, therapeutic resistance and disease recurrence persist as major clinical challenges [1]. Mounting evidence implicates the tumor immune microenvironment (TIME) as a fundamental driver of this resistance, wherein immunosuppressive cellular networks—including exhausted CD8^+^ T lymphocytes, expanded regulatory T cell (Treg) populations, and protumoral M2-polarized tumor-associated macrophages (TAMs)—establish a niche that facilitates immune evasion and tumor progression [3,4]. While immune checkpoint inhibitors have revolutionized treatment paradigms, their efficacy remains limited in hormone receptor-positive breast cancer. Moreover, compensatory activation of alternative signaling pathways and adaptive immune resistance mechanisms underscore the need for multi-targeted therapeutic strategies capable of simultaneously modulating diverse oncogenic and immunoregulatory nodes [5]. Therefore, food-medicinal substances, which integrate nutritional support with pharmacological activity, offer accessible and generally well-tolerated alternatives for cancer intervention.

In this context, Traditional Chinese Medicines (TCMs) emerge as promising candidates for integrated cancer therapy, owing to their inherent polypharmacological profiles and systems-level modulatory effects. The network pharmacology paradigm, a computational framework that maps the complex interactions between multi-component botanical formulations and their biological targets, offers a theoretically grounded approach to decode the mechanistic basis of TCM efficacy [6]. This methodology is particularly suited for investigating immune-modulating botanicals, as it can capture synergistic interactions across inflammatory signaling cascades, immune cell differentiation pathways, and tumor-stromal crosstalk networks that would be overlooked by conventional single-target drug discovery models.

*Cynomorium songaricum* Rupr. (CS), a perennial parasitic herb indigenous to arid regions of Central Asia, has been employed for centuries in TCM to “tonify kidney-yang” and enrich blood essence [7,8]. Modern cultivation advances in Gansu Province have enabled sustainable production. Phytochemical studies have revealed that CS is rich in diverse bioactive constituents, including flavonoids, terpenoids, and phenolic acids [9,10], which contribute to its reported pharmacological activities such as immunomodulation and anti-tumor effects [11]. However, despite these promising leads, critical gaps in knowledge severely hinder its clinical translation and pharmaceutical development for breast cancer. Specifically, a comprehensive and systematic characterization of its chemical constituents is still lacking. Moreover, its anti-breast cancer mechanisms remain poorly understood, and its potential influence on the TIME has not yet been explored. The absence of such knowledge represents a major bottleneck in advancing CS as a credible therapeutic agent.

To address these gaps, we conducted an integrated study combining state-of-the-art chemical analysis with computational biology. We first employed UHPLC-Q-Exactive Orbitrap MS/MS to systematically profile the chemical constituents of CS. Next, network pharmacology was applied to screen for bioactive drug-like compounds and predict their potential targets. By focusing on the intersection of CS targets, breast cancer-related genes, and immune system-related genes, we specifically aimed to decipher the immunomodulatory dimension of its anti-cancer activity. Finally, molecular docking was performed to validate the binding affinities between the core potential drug-like compounds and prioritized candidate targets. This study provides a comprehensive chemical foundation and a testable mechanistic framework for developing CS-based multi-targeted immunotherapies, while exemplifying a replicable paradigm for decoding complex botanical medicines through systems pharmacology approaches.

## 2. Results

### 2.1. Main Chemical Components of Cynomorium songaricum Metabolites

**Figure 1:**
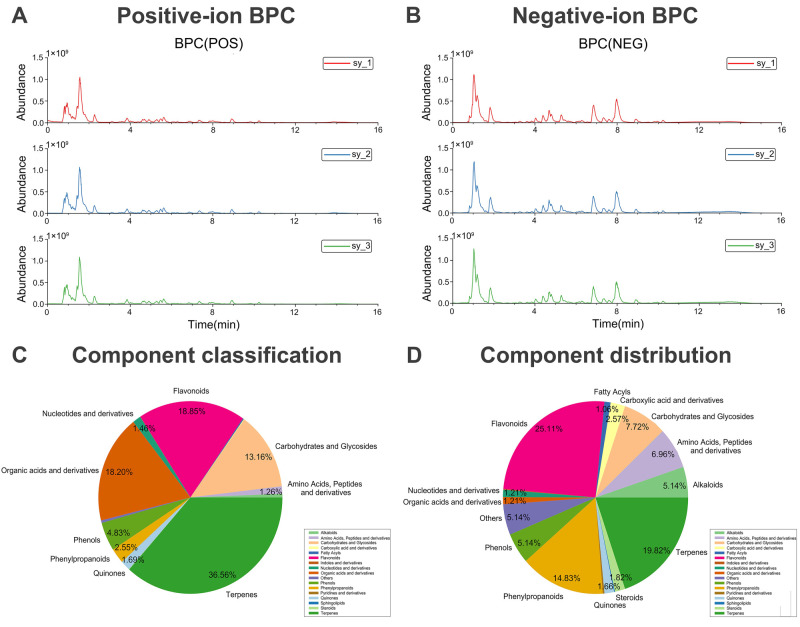
Characterization of the chemical components of CS. Base peak chromatograms (BPCs) of CS were obtained by LC–MS analysis in (**A**) positive and (**B**) negative ion modes. (**C**) Distribution of the chemical constituents of CS by classification. (**D**) Quantitative distribution of the identified constituents. Percentages may not sum to 100% due to rounding. Segments representing <1% are not labeled with specific percentages in the main figure; see Table S1 for complete data.

UHPLC-Q-Exactive Orbitrap MS/MS analysis in both positive and negative ion modes identified 1100 putative chemical constituents in CS (Figure 1A,B, Table S1). All metabolic profiling analyses were performed in three independent technical replicates (*n* = 3) prepared from the same CS batch. Peak alignment and integration were accomplished using Progenesis QI v3.0 and matched against a traditional Chinese medicine (TCM) database; only features with a coefficient of variation (CV) < 15% across replicates were retained for downstream identification. These metabolites were categorized into 17 major classes, with relative abundances and quantitative distributions presented in Figure 1C,D. Subsequent screening based on oral bioavailability (OB ≥ 30%), drug-likeness (DL ≥ 0.18), and high gastrointestinal absorption identified 84 potential drug-like compounds for further investigation (Table S2). This approach of integrating LC-MS-based metabolic profiling with subsequent bioactive compound screening aligns with recent advances in decoding the material basis of traditional Chinese medicine formulas [12].

### 2.2. Screening of Common Genes for Drugs and Diseases

**Figure 2:**
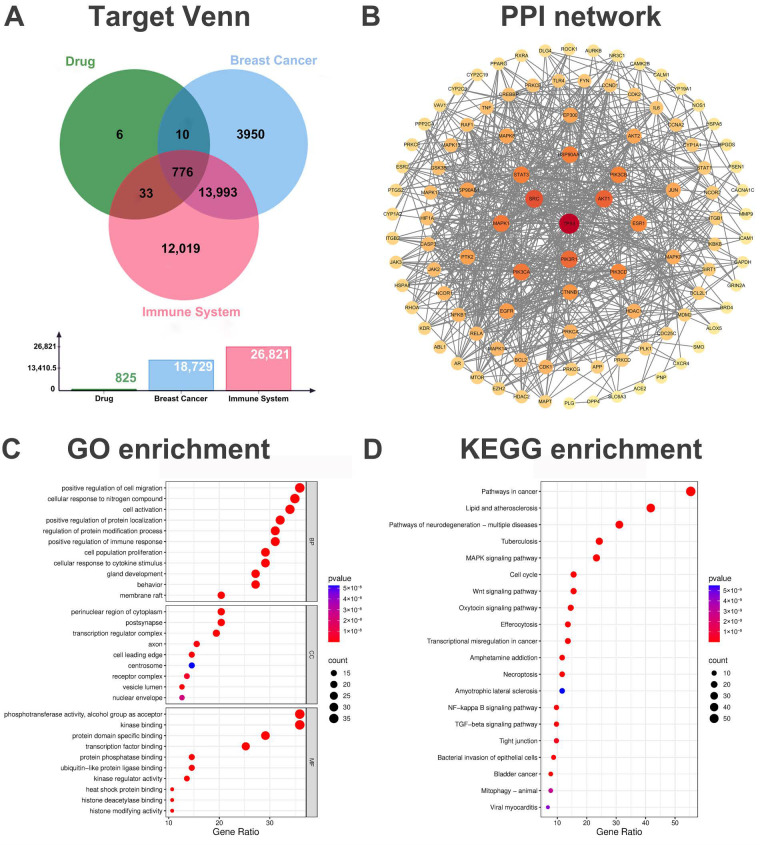
Network pharmacological analysis of CS for the treatment of breast cancer. (**A**) Venn diagram illustrating the overlapping targets among CS, breast cancer, and the immune system. (**B**) Protein–protein interaction (PPI) network, where node size and color intensity reflect the target’s importance as a hub within the network. (**C**) Gene Ontology (GO) enrichment analysis. (**D**) Kyoto Encyclopedia of Genes and Genomes (KEGG) pathway enrichment analysis.

To identify potential therapeutic targets, we retrieved breast cancer-related genes from GeneCards, OMIM, and DisGeNET databases. After removing duplicates, we compiled 18,729 breast cancer-associated targets and 26,821 immune system-related targets. Intersection with the predicted targets of CS components identified 776 common genes (Figure 2A). These overlapping targets include prioritized candidate proteins such as AKT1 [13], STAT3 [14], TP53 [15], PIK3CA [16], and MTOR [17], which are known to play central roles in both oncogenesis and immune regulation, suggesting that CS may exert coordinated effects on tumor cells and the immune landscape through these hubs.

### 2.3. Construction and Analysis of the Protein–Protein Interaction (PPI) Network

The protein–protein interaction (PPI) network, constructed using the intersecting targets of CS active components and anti-breast cancer-related genes, consisted of 590 nodes and 2487 edges. Core targets within the network were identified using the CentiScaPe 2.2 plugin in Cytoscape based on three centrality metrics: degree, closeness, and betweenness centrality. The average values for these metrics were as follows: Betweenness (unDir) = 1798.389831, Closeness (unDir) = 0.000434019929702828, Degree (unDir) = 8.43050847457627. Nodes exceeding these averages in all three criteria were classified as core targets, reflecting their pivotal roles in mediating cross-pathway communication and biological functionality (Figure 2B).

Notably, the top core targets identified in the PPI network are widely recognized for their dual involvement in breast cancer progression and immune regulation. AKT1 serves as a central signaling hub that integrates growth factor and cytokine signaling to promote tumor cell survival and concurrently modulates T cell activation and differentiation—thereby bridging oncogenic proliferation with immune response pathways [18]. STAT3, a prioritized candidate transcription factor, not only drives the expression of pro-inflammatory cytokines within the TIME and recruits immunosuppressive cells but also regulates B cell and T cell activities [14,19]. Meanwhile, MTOR coordinates tumor cell cycle progression and DNA damage repair while also influencing the polarization of tumor-associated macrophages (TAMs) and the cytotoxic function of natural killer (NK) cells [20]. As central nodes in the PPI network, these core targets connect CS-derived bioactive components to downstream anti-tumor and immune-modulating pathways, underscoring their essential role in mediating the “multi-component, multi-target” therapeutic mechanism of CS against breast cancer.

### 2.4. GO Functional Enrichment Analysis

GO functional enrichment analysis identified 3894 significant entries (*p* < 0.05), including 2553 biological process (BP), 152 cellular component (CC), and 1189 molecular function (MF) terms (Figure 2C). Among BP terms, processes critically involved in TIME modulation were significantly enriched. These included “inflammatory response,” “positive regulation of immune response,” “cellular response to cytokine stimulus,” and “positive regulation of cell migration,” collectively pointing to the activation of immune cells, cytokine signaling, and the regulation of immune cell infiltration within the tumor microenvironment. For instance, regulation of “inflammatory response” influences the accumulation of myeloid-derived suppressor cells (MDSCs), a hallmark of immunosuppressive TIME, by attenuating the secretion of pro-inflammatory mediators that recruit these cells [21,22]. “Positive regulation of immune response” enhances the cytotoxic activity of CD8^+^ T cells, improving their capacity to recognize and eliminate tumor cells [23]. Meanwhile, “cellular response to cytokine stimulus” modulates bidirectional signaling between immune cells and tumor cells, counteracting the induction of T cell exhaustion by cytokines such as IL-10 and TGF-β [24]. Enriched CC and MF terms, such as “transcription regulator complex,” “kinase binding,” and “protein phosphatase binding,” further suggested that CS components may intervene in these structural and functional modules to exert multi-faceted control over the TIME.

### 2.5. KEGG Enrichment Analysis

KEGG pathway enrichment analysis identified 201 significant pathways (*p* < 0.05), including general pathways such as Pathways in cancer, MAPK signaling pathway, and Wnt signaling pathway (Figure 2D). Of particular importance was the significant enrichment of the NF-κB and TGF-β signaling pathways, both recognized as core regulators of the TIME. Hyperactivation of the NF-κB pathway promotes the recruitment of immunosuppressive cells like MDSCs and M2-type TAMs and the secretion of pro-inflammatory cytokines, thereby facilitating immune evasion [25,26]. The TGF-β pathway contributes to immunosuppression by driving Treg differentiation and epithelial-mesenchymal transition (EMT), while simultaneously impairing CD8^+^ T cell and NK cell cytotoxicity [27,28]. Additionally, TGF-β drives epithelial-mesenchymal transition (EMT) in tumor cells, thereby enhancing metastatic potential [29]. The enrichment of these pathways implies that CS active components may cooperatively suppress NF-κB and TGF-β signaling, thereby reversing the immunosuppressive TIME and restoring anti-tumor immunity.

### 2.6. Analysis of the “Potential Drug-like Compounds–Target–Pathway” Network

**Figure 3:**
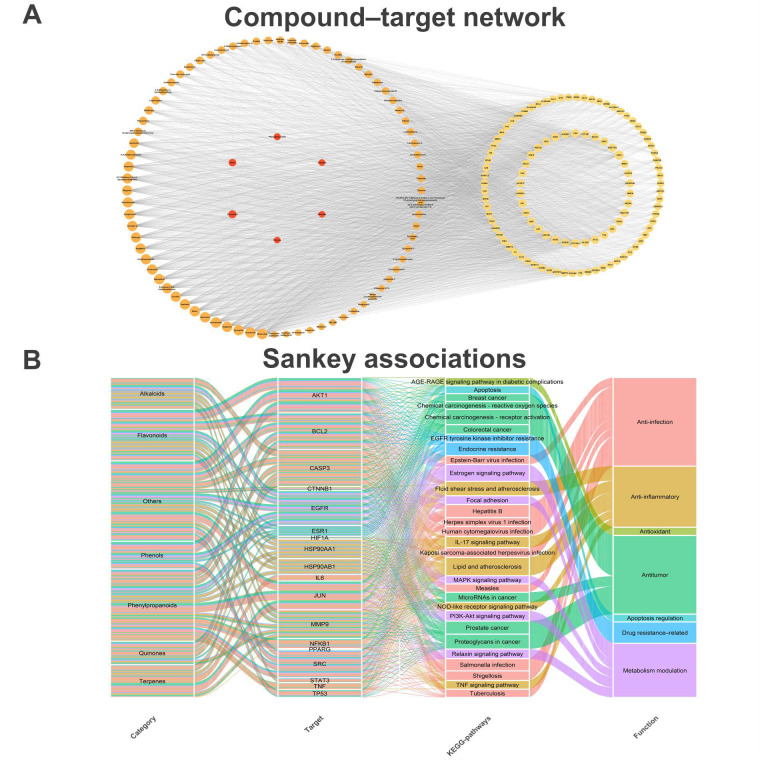
Integrated network analysis of CS in breast cancer treatment. (**A**) Network depicting substance categories, drug-like compounds, and core targets. Nodes are color-coded: red for substance categories, orange for drug-like compounds, and yellow for core targets. Larger nodes indicate compounds with key roles in interacting with core targets. (**B**) Sankey diagram showing the associations among substance categories (e.g., flavonoids, alkaloids), corresponding targets, enriched KEGG pathways, and core biological functions. The four tiers (left to right) represent substance categories, targets, KEGG pathways, and biological functions, respectively. Line widths correspond to the number of associated targets or the extent of pathway enrichment, with distinct colors used to differentiate core biological functions.

Based on the KEGG pathway enrichment results, these targets implicated in the significant pathways were mapped back to their corresponding potential drug-like compounds. A comprehensive network integrating substance categories, compounds, and targets was constructed using Cytoscape (version 3.10.1). The resulting network comprised 188 nodes and 3018 edges, encompassing 6 major substance categories, 77 potential drug-like compounds, and 539 targets (Figure 3A). All edges represent unweighted, predicted associations with confidence derived from database prediction scores and enrichment significance, not continuous biophysical parameters. To further elucidate the multi-scale relationships among these entities, a Sankey diagram was generated to visualize the specific connections between potential drug-like compounds, their corresponding targets, and the associated signaling pathways (Figure 3B). This network topology demonstrates that the active components of CS can act synergistically against breast cancer by modulating multiple targets across different biological pathways, reflecting a characteristic “multi-component, multi-target, multi-pathway” therapeutic mechanism.

### 2.7. Molecular Docking Verification of Different Active Components and Core Targets in Cynomorium songaricum

To validate the interactions between CS’s chemical components and the core targets identified from the PPI network, molecular docking was performed using the top 20 core targets ranked by the CentiScaPe 2.2 plugin in Cytoscape. The binding affinity was evaluated based on binding energy, with lower values indicating more stable interactions. All docking runs were repeated three independent times with distinct random seeds; binding energies are reported as the mean of the three replicates.

**Figure 4:**
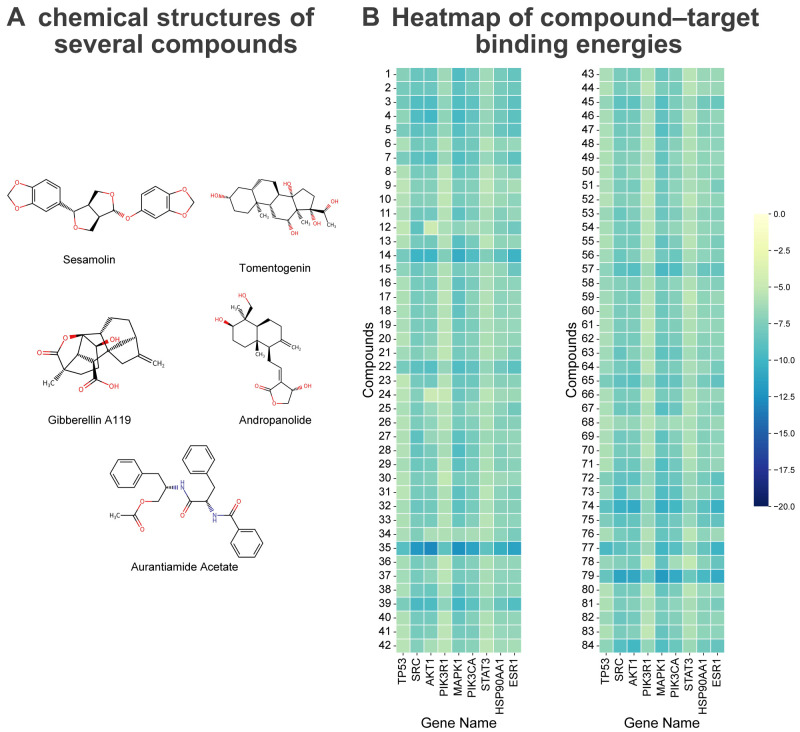
Representative compound structures and compound–target docking heatmap. (**A**) 2D chemical structures of compounds highlighted in the text (Tomentogenin, Sesamolin, Andropanolide, liquiritigenin, and paeoniflorin). In panel (**A**), oxygen atoms are shown in red and nitrogen atoms in blue. (**B**) Heatmap of predicted binding energies between 84 drug-like compounds and core targets (lower values indicate more favorable binding).

The docking outcomes are summarized by highlighting representative bioactive compounds in Figure 4A, where the 2D chemical structures of the key compounds discussed in the text are shown. For transparency and reproducibility, the corresponding structure identifiers (SMILES and/or InChIKey) together with the full compound–target docking energy matrix are provided in Supplementary Table S3. The average binding energies for the top four core targets were −6.41, −8.18, −7.89, and −5.92 kcal/mol, respectively. According to commonly accepted criteria, a docking energy below −4.25 kcal/mol (1 cal ≈ 4.186 J) suggests detectable binding activity, whereas values < −5.0 kcal/mol and <−7.0 kcal/mol indicate good and high-affinity interactions, respectively. All 84 potential drug-like compounds exhibited binding energies below −4.25 kcal/mol, suggesting widespread binding potential across the prioritized targets, and most compounds showed energies less than −5.0 kcal/mol, consistent with generally favorable predicted affinity (Supplementary Table S3). Notably, several compounds—including Gibberellin A119, Tomentogenin, Sesamolin, Andropanolide, and Aurantiamide acetate—showed high-affinity interactions (energy < −7.0 kcal/mol) with multiple core proteins, with Tomentogenin, Sesamolin, and Andropanolide consistently ranking among the strongest binders across several prioritized candidate targets.

**Figure 5:**
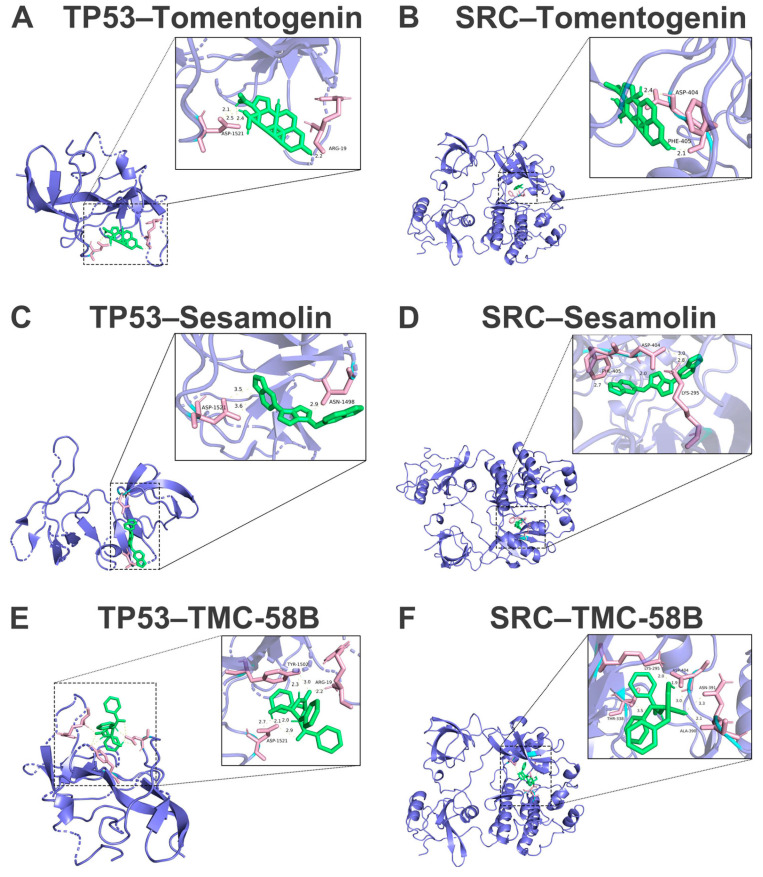
Molecular docking visualization of potential drug-like compounds with core proteins. Representative docking poses are displayed for (**A**) TP53 (PDB: 2IG0) with Tomentogenin; (**B**) SRC (PDB: 2SRC) with Tomentogenin; (**C**) TP53 with Sesamolin; (**D**) SRC with Sesamolin; (**E**) TP53 with TMC-58B; and (**F**) SRC with TMC-58B.

Representative binding poses and key intermolecular contacts are shown in Figure 5. Sesamolin inhibits TP53 at micromolar level (Ki = 2.01 µM; ΔG = −7.77 kcal mol^−1^), employing a bifurcated hydrogen-bond architecture with Asp-1521 (3.5, 3.6 Å) reinforced by a 2.9 Å contact to Asn-1498; the intermolecular energy totals –8.67 kcal mol^−1^. At SRC, Sesamolin retains comparable activity (Ki = 2.4 µM; ΔG = −7.67 kcal mol^−1^) through simultaneous engagement of Asp-404 (2.0, 3.0 Å), Lys-295 (2.8 Å) and Phe-405 (2.7 Å), registering an intermolecular term of –8.56 kcal mol^−1^. Tomentogenin achieves sub-micromolar TP53 blockade (Ki = 364 nM; ΔG = −8.78 kcal mol^−1^) by anchoring its steroid core via three hydrogen bonds to Asp-1521 (2.1, 2.2, 2.4 Å) and an auxiliary Arg-19 bond (2.2 Å), generating −10.57 kcal mol^−1^ of intermolecular stabilization. Nanomolar SRC inhibition is realized by Tomentogenin (Ki = 61 nM; ΔG = −9.85 kcal mol^−1^), wherein dual symmetric hydrogen bonds to Asp-404 (2.4 Å each) and an aromatic interaction with Phe-405 (2.1 Å) produce an intermolecular energy of −11.64 kcal mol^−1^.

These docking results suggest generally favorable interactions between CS-derived drug-like compounds and core breast-cancer-related targets. The prevalence of good-to-strong binding scores supports the plausibility of a ‘multi-component, multi-target’ mode of action and provides testable hypotheses for downstream experimental validation.

### 2.8. Molecular Dynamics Simulations Validate the Stability of Key Complexes

#### 2.8.1. System Stability

**Figure 6:**
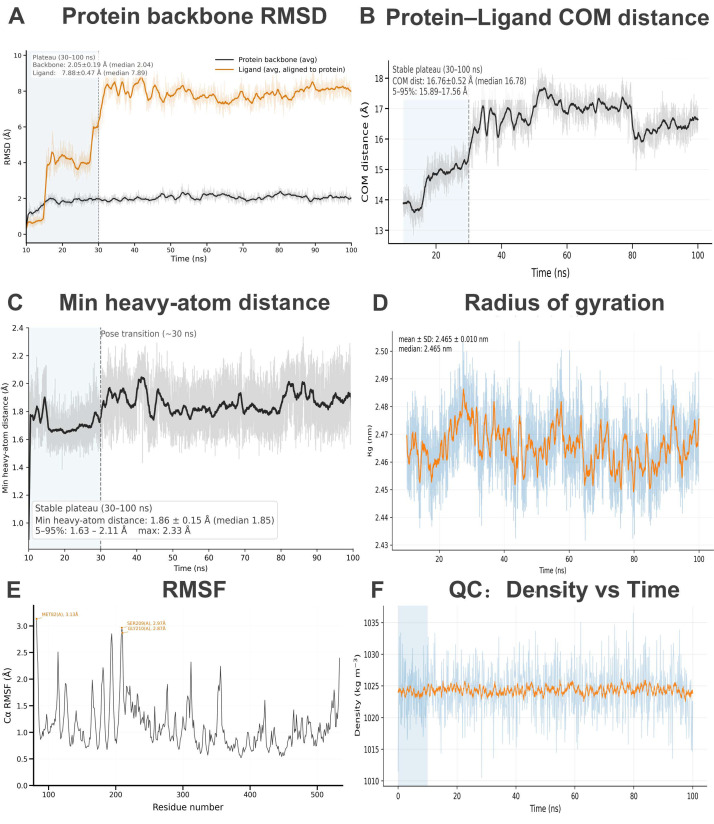
Conformational stability of SRC–Tomentogenin during molecular dynamics simulations. (**A**) Time evolution of the protein backbone RMSD (black) and ligand RMSD aligned to the protein (orange) for t ≥ 10 ns. A stable plateau was observed after the pose transition (~30 ns), and quantitative statistics were computed from 30–100 ns (values shown in panel). (**B**) Protein–ligand center-of-mass (COM) distance for t ≥ 10 ns, showing a stable binding-distance plateau in 30–100 ns; mean ± SD, median, and 5–95% range are reported in the panel. (**C**) Minimum heavy-atom distance between protein and ligand for t ≥ 10 ns, highlighting the pose-transition region (~30 ns) and the stable plateau (30–100 ns). Statistics (mean ± SD, median, 5–95%, and maximum) were calculated over 30–100 ns. (**D**) Radius of gyration (Rg) of the protein for 10–100 ns, indicating overall compactness and the absence of large-scale unfolding during the simulation (mean ± SD and median shown). (**E**) Residue-wise root-mean-square fluctuation (RMSF) of protein Cα atoms computed over 30–100 ns, with the most flexible residues annotated. (**F**) System density versus time (QC) demonstrating stable equilibration under production conditions; the shaded region indicates the initial equilibration period.

The trajectories reached stable thermodynamic conditions, as indicated by the equilibration of temperature, pressure, and density (Figure S1; Table S6). The system density quickly converged and fluctuated around ~1024–1025 kg m^−3^ without long-term drift, indicating stable NPT equilibration (Figure 6F; Table S6).

To evaluate the dynamic stability of the docked SRC–Tomentogenin pose, we performed a 100 ns all-atom MD simulation. After the initial relaxation period, the SRC backbone RMSD reached a stable plateau and remained around 2.05 ± 0.19 Å during 30–100 ns, indicating a well-converged protein structure (Figure 6A; Table S5). The protein compactness was also stable, with Rg fluctuating narrowly around 2.465 ± 0.010 nm (10–100 ns) (Figure 6D; Table S5). Residue-level flexibility (Cα RMSF, 30–100 ns) showed that higher fluctuations were confined to a few flexible regions (e.g., MET82, SER209, GLY210), while most residues exhibited low mobility (Figure 6E; Table S7).

#### 2.8.2. Persistent Pocket Occupancy

Although the ligand RMSD exhibited a higher absolute value (consistent with internal conformational adjustment/rotation within the pocket), the protein–ligand COM distance remained stable in the plateau region (16.76 ± 0.52 Å, 5–95%: 15.89–17.56 Å), suggesting persistent binding-site occupancy without dissociation (Figure 6B; Table S5). The minimum heavy-atom distance between protein and ligand remained low during the stable window, supporting sustained close contacts after the pose transition (~30 ns) (Figure 6C; Table S5).

**Figure 7:**
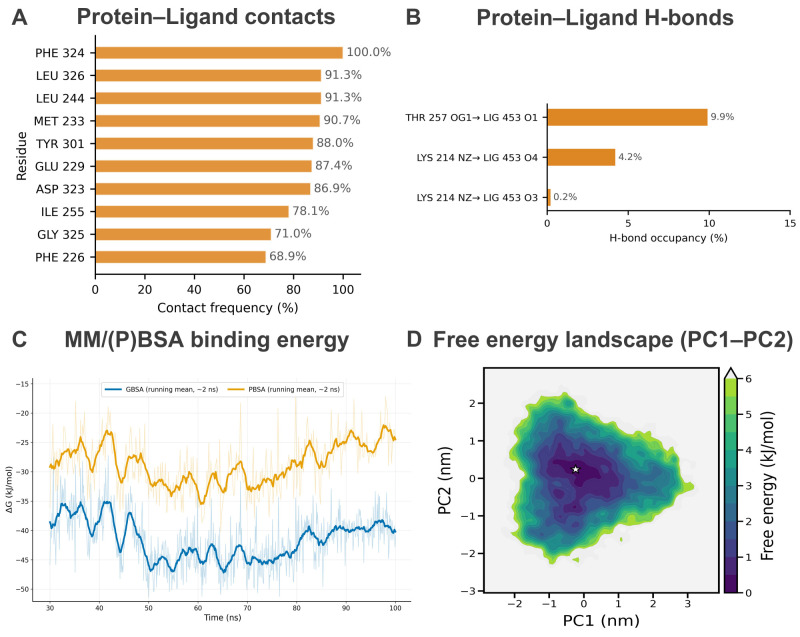
Interaction and energetic characterization of SRC–Tomentogenin during the stable plateau. (**A**) Protein–ligand contact frequency of the top residues, calculated over 30–100 ns using a distance cutoff of <4.0 Å (frame stride = 50, *n* = 141 frames). (**B**) Protein–ligand hydrogen-bond occupancy of the dominant H-bond pairs over 30–100 ns, defined by donor–acceptor distance ≤ 3.5 Å and angle ≥ 150° (stride = 50). (**C**) MM/(P)BSA binding free energy (ΔG) time series computed for 30–100 ns (running mean window ~2 ns; shading shows raw values). (**D**) Free energy landscape (FEL) projected onto the first two principal components (PC1–PC2) for conformations sampled in 30–100 ns; the star indicates the global minimum basin, and the color scale represents relative free energy (kJ/mol).

Contact analysis further revealed long-lived interactions with a set of pocket residues, dominated by hydrophobic contacts (e.g., PHE324, LEU326, LEU244, MET233, TYR301) and complemented by polar residues (GLU229, ASP323) (Figure 7A; Table S8).

#### 2.8.3. Hydrogen-Bond Persistence

In the production phase (30–100 ns), the protein–ligand hydrogen-bond network was stable, with an average of 0.98 ± 0.53 hydrogen bonds per frame. The system predominantly maintained one hydrogen bond (74.7% of frames), while frames with zero hydrogen bonds accounted for 13.9%, indicating transient breaking/reforming events typical of solvated complexes. Occasional formation of a second hydrogen bond (10.6% of frames) was observed. Importantly, the most persistent hydrogen bond involved GLU229 (occupancy 71.0%), suggesting an anchoring interaction that helps stabilize Tomentogenin in the binding pocket (Figure 7B and Figure S4A–B; Table S9).

#### 2.8.4. Binding Energetics and Conformational Landscape (MM/(P)BSA, PCA/FEL)

To further quantify binding propensity, MM/GBSA and MM/PBSA calculations were performed on the equilibrated trajectory (30–100 ns). The estimated binding free energies remained consistently negative over time, supporting sustained association of Tomentogenin with SRC (Figure 7C). Energy decomposition suggested that binding was mainly driven by favorable van der Waals interactions, with additional electrostatic contributions partially compensated by polar solvation penalties (Figure S4C; Table S10).

PCA revealed that the first few principal components captured the majority of large-scale motions (Figure S4E–F). The free-energy landscape projected onto PC1–PC2 exhibited a dominant low-energy basin, suggesting that the SRC–Tomentogenin complex predominantly populates a stable conformational state during 30–100 ns (Figure 7D).

#### 2.8.5. Supporting and Negative Systems

For comparison, the SRC–Sesamolin complex displayed similarly stable protein dynamics and compact binding geometry over 30–100 ns and is provided as supporting evidence in the Supplementary Materials (Figure S2; Table S5). In contrast, TP53–Sesamolin showed intermittent separation events (e.g., a non-negligible fraction of frames with minimum heavy-atom distance > 10 Å and a maximum separation event) and is therefore reported as negative/inconclusive without downstream mechanistic or energetic interpretation (Figure S3; Table S5).

## 3. Discussion

This study addresses critical gaps in the current understanding of *Cynomorium songaricum* Rupr. (CS), including its incomplete chemical characterization, unclear anti-breast cancer mechanisms, and unexplored capacity to modulate the tumor immune microenvironment (TIME). By integrating UHPLC-Q-Exactive Orbitrap MS/MS, network pharmacology, molecular docking, and MD simulations, we have systematically resolved these ambiguities, establishing a clear material basis and mechanistic framework to support the therapeutic application of CS in breast cancer.

A key finding of this work is the identification of a TIME-modulating mechanism driven by multi-component synergy in CS, linking our bioinformatic predictions to established immune escape pathways in breast cancer [16,17,18,19,20,21,22,23,24,25,26,27,28]. Comprehensive profiling identified 1100 constituents, including 84 drug-like compounds such as paeoniflorin and liquiritigenin (known for their immunomodulatory properties), and revealed 776 intersection genes that link CS metabolites to breast cancer pathogenesis and immune regulation. Functional enrichment analysis showed that these targets converge on immune-related signaling cascades, particularly NF-κB and TGF-β pathways known to promote immune evasion by recruiting immunosuppressive cells and impairing effector T-cell function [30,31]. These findings support a plausible synergistic hypothesis in which CS drug-like compounds (e.g., Tomentogenin and Sesamolin) may modulate NF-κB-associated signaling, while flavonoids such as liquiritigenin could influence TGF-β-related processes, and phenolics such as Andropanolide may contribute to TP53-associated responses. Collectively, these predicted interactions suggest a plausible hypothesis that CS constituents may cooperatively modulate the TIME from an immune-evasive toward a more immunostimulatory state, which warrants further experimental validation. Molecular docking indicated high-affinity interactions between prioritized candidate compounds (e.g., Tomentogenin and Sesamolin) and core targets, supporting the biological plausibility of our in silico findings [32,33]. To reduce over-interpretation from static docking, we further performed explicit-solvent molecular dynamics (MD) simulations as an orthogonal validation step. Given the multi-target scope of this study and the large number of component–target pairs, we adopted a focused reporting strategy: the main text presents a complete MD evidence chain for a representative complex (SRC–Tomentogenin) (Figure 6 and Figure 7), while additional systems are provided as supporting/negative results in the Supplementary Materials (Figures S1–S4; Tables S5–S10). The SRC–Tomentogenin trajectory exhibited a well-defined stable plateau during the equilibrated window, with persistent pocket occupancy, consistent interaction signatures, favorable MM/(P)BSA binding energetics, and a concentrated low-energy basin on the PCA-based free energy landscape (Figure 6 and Figure 7). Therefore, the following mechanistic discussion prioritizes SRC–Tomentogenin as the most stable and interpretable representative case under dynamic conditions, while maintaining the network-level perspective of multi-component, multi-target regulation. These results are consistent with prior reports of the immunomodulatory properties of CS and the role of TIME dysregulation in breast cancer [34], thereby reinforcing the credibility of the proposed “multi-component, multi-target, multi-pathway” mechanism.

**Figure 8:**
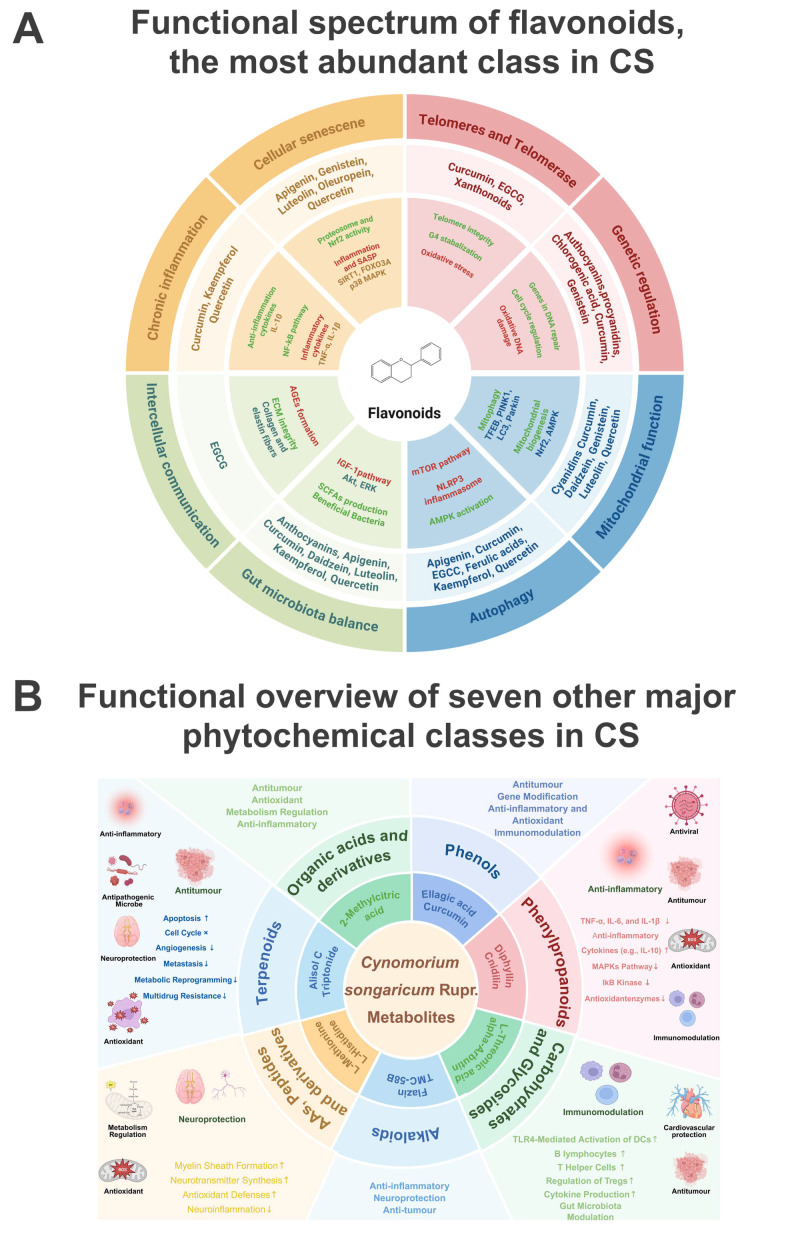
Functional landscape of major phytochemical classes in CS. (**A**) Functional spectrum of flavonoids, the predominant phytochemical in CS, summarizing their reported bioactivities, relevant immune- and cancer-related pathways, and representative compounds. (**B**) Functional overview of seven other major phytochemical classes, including Amino Acids, Peptides and Derivatives; Phenylpropanoids; Phenols; Alkaloids; Carbohydrates and Glycosides; Terpenoids; and Organic Acids and Derivatives, highlighting their primary bioactivities, key immune- and cancer-related pathways, and representative constituents. Arrow symbols (↑/↓) indicate an increase/decrease in expression, translation, or functional level. This figure was created with BioRender.com ((**A**) Mao, Z. (2026) https://BioRender.com/pucp7l0; (**B**) z, Z. (2026) https://BioRender.com/zgl2hnq).

In addition to the network- and pathway-level findings, we further summarized the functional spectrum of the major phytochemical classes identified in CS (Figure 8A). Flavonoids, the most abundant class, have been reported to exert antioxidant, anti-inflammatory, immunomodulatory and anticancer effects, frequently acting on signaling pathways such as NF-κB, PI3K/AKT and TGF-β in tumor and immune cells [35,36,37,38]. In parallel, Amino Acids, Peptides and derivatives, Phenylpropanoids, Phenols, Alkaloids, Carbohydrates and Glycosides, Terpenoids, and Organic acids and derivatives collectively contribute additional activities, including modulation of cytokine production, protection against oxidative stress, metabolic regulation, neuroprotection and cardiovascular protection [39,40,41].

Notably, many of these reported activities converge on the immune- and cancer-related processes highlighted by our network pharmacology analysis, including regulation of inflammatory signaling, cell survival pathways and components of the TIME. Thus, the functional landscape depicted in Figure 8B supports a multi-component, functionally complementary pharmacological profile of CS and provides a biological context for the multi-target, immune-related mechanism that we propose for its anti-breast cancer effects.

This study marks a paradigm shift from earlier CS research, which has largely focused on isolated components (e.g., polysaccharides) or general in vitro cytotoxicity [42], without systematically exploring its full chemical diversity or potential for TIME modulation. In contrast, our work embraces the holistic philosophy of traditional Chinese medicine by emphasizing the synergistic interactions among diverse metabolites in CS, rather than isolating single compounds. This approach not only elucidates the anti-breast cancer mechanism of CS but also demonstrates how integrating modern analytical techniques with network pharmacology can unlock the therapeutic potential of multi-component medicinal plants—moving beyond the “single-component, single-target” framework that has limited progress in traditional medicine research.

While our findings provide valuable insights, certain inherent limitations point to critical future research directions. First, although we identified potential bioactive compounds and their targets, further in vitro validation is necessary to confirm the bioactivity of individual drug-like constituents. In addition, although we added 100 ns all-atom MD simulations to strengthen pose stability assessment beyond static docking, these simulations were conducted as single-trajectory runs per system and therefore do not fully capture replica-to-replica variability. Longer simulations and/or multiple independent replicas would further strengthen statistical confidence, and MM/(P)BSA should be interpreted comparatively rather than as an absolute binding affinity. Second, the proposed “multi-component, multi-pathway” regulatory model requires more in-depth investigation to clarify the nature and dynamics of synergistic interactions among CS components. Third, in vivo validation using breast cancer animal models will be essential to verify whether CS can effectively suppress tumor growth and remodel the TIME in a physiologically relevant context. These limitations reflect common challenges in translating computational predictions into clinical applications and do not diminish the core contributions of this study.

A primary limitation of this study is the pan-breast cancer analytical approach, which does not account for the significant heterogeneity of the TIME across molecular subtypes. These subtype-specific TIME characteristics may differentially influence the therapeutic efficacy and target prioritization of CS constituents. Future studies integrating transcriptomic data from The Cancer Genome Atlas (TCGA) for subtype-specific network pharmacology analysis, coupled with experimental validation in patient-derived xenograft (PDX) models or organoids, would provide more precise mechanistic insights for personalized immunotherapy development.

In summary, this study fills key knowledge gaps in CS research by clarifying its chemical composition, identifying immune-focused anti-breast cancer mechanisms, and validating component–target interactions. Beyond its specific implications for CS, the integrated analytical framework presented here offers a replicable paradigm for decoding the immune-related efficacy of other understudied medicinal plants—bridging ethnopharmacological knowledge with contemporary molecular biology to accelerate the modernization of traditional medicine. This integrated approach is also consistent with recent network pharmacology studies that incorporate complementary validation and omics-based evidence to improve robustness and interpretability in complex herbal systems [43]. Looking forward, we plan to validate key compounds in vitro and in vivo, optimize CS-based formulations (e.g., extracts or nanoformulations) to improve bioavailability, and advance toward clinical trials for breast cancer patients with TIME-related therapeutic resistance. Through these efforts, this work not only supports the development of CS as a multi-targeted, low-toxicity anti-cancer agent but also contributes to expanding treatment options for patients with unmet clinical needs—fulfilling the study’s original goal of addressing the persistent therapeutic challenges in breast cancer.

## 4. Materials and Methods

### 4.1. Chemical Composition Analysis of Cynomorium songaricum

#### 4.1.1. Instruments and Materials

CS was obtained from Jiuquan Jinsuoyang Biotechnology Co., Ltd., Jiuquan, China and stored at −80 °C at the Medical Frontier Innovation Research Center. The following equipment were used: F-060SD ultrasonic cleaner (Shenzhen Fuyang Technology Co., Ltd., Shenzhen, China), TYXH-I Vortex Oscillator (Shanghai Hannuo Instrument Co., Ltd., Shanghai, China), TGL-16MS High-speed benchtop refrigerated centrifuge (Shanghai Luxiangyi Centrifuge Instrument Co., Ltd., Shanghai, China). Liquid chromatography–mass spectrometry (LC–MS) analysis was performed on a Waters ACQUITY UPLC system (Waters Technologies Shanghai Co., Ltd., Shanghai, China) equipped with an ACQUITY UPLC I-Class HF and an ACQUITY UPLC HSS T3 column (100 mm × 2.1 mm, 1.8 μm). Chromatographic-grade methanol, acetonitrile, and formic acid were obtained from Thermo Fisher Scientific, Inc. (Waltham, MA, USA). High-resolution mass spectrometry was performed using a Thermo Q Exactive Orbitrap instrument (Thermo Fisher Scientific, Inc.).

#### 4.1.2. Preparation of Sample Solution

CS samples were pulverized into a fine powder using liquid nitrogen. Exactly 100 mg of the powder was weighed into a 1.5 mL centrifuge tube, and 1 mL of aqueous solution containing 4 μg/mL mixed internal standard was added. The mixture was vortexed for 60 s, pre-cooled in a −40 °C for 2 min, and then homogenized in a grinder at 60 Hz for 2 min. After treatment in an ice-water bath, the sample was extracted via ultrasonication for 60 min. The extract was centrifuged at 14,000 r/min for 10 min at 4 °C. Subsequently, 200 μL of the supernatant was transferred into an LC-MS compatible vial with an insert, which was immediately sealed and stored for further analysis.

#### 4.1.3. UHPLC-Q-Exactive Orbitrap MS/MS Analysis

The UHPLC-Q-Exactive Orbitrap MS/MS platform has been successfully applied in the comprehensive metabolite profiling of medicinal plants, including a recent study on Lanzhou lily [44], validating the reliability of this analytical approach. Chromatographic separation was performed on an ACQUITY UPLC I-Class HF system coupled to a Q-Exactive high-resolution mass spectrometer.

Chromatographic conditions included an ACQUITY UPLC HSS T3 column (100 mm × 2.1 mm, 1.8 μm). The mobile phase consisted of 0.1% FA in water (A) and acetonitrile (B). A constant flow rate (0.35 mL/min) was maintained. 0–2 min, 95% A, 5% B. At 4 min, 70% A, 30% B. At 8 min, 50% A, 50% B. At 10 min, 20% A, 80% B. At 14 min, 0% A, 100% B, and maintain until 15 min. At 15.1 min, 95% A, 5% B and hold until 16 min. The injection volume was 5 μL.

Mass spectrometric analysis was conducted using a HESI ion source in both positive and negative ionization modes. The scanning mode was set to Full MS/dd-MS^2^ (Top 8), with a mass range of *m*/*z* 100–1500. The capillary temperature was maintained at 320 °C. Detailed mass spectrometry parameters are provided in Table S4.

#### 4.1.4. Identification of Chemical Components in *Cynomorium songaricum*

Raw data were processed using Progenesis QI v3.0 (Nonlinear Dynamics, Newcastle, UK) for peak detection, identification, alignment, and normalization. Compound identification was based on accurate mass to charge ratio (*m*/*z*), secondary fragmentation, and isotope distribution using the LuMet-TCM (LuMing Biotechnology Co., Ltd., Shanghai, China). Compounds with a total identification score ≥ 40 were retained. After merging and deduplicating entries from both ionization modes, the relative peak area of metabolites was normalized to 100%, yielding a qualitative and quantitative data matrix. The main parameters were 5 ppm precursor tolerance and 10 ppm product tolerance.

To ensure identification accuracy, extracted ion chromatograms (EICs) and MS/MS spectra of each component were reviewed, with fragment ions annotated and putative structures assigned.

### 4.2. Network Pharmacology Study

#### 4.2.1. Prediction of Component Action Targets

Identified components were evaluated using SwissADME (https://www.swissadme.ch/index.php, accessed on 11 March 2025) [45] and the TCMSP database [46] (https://www.tcmsp-e.com/tcmsp.php, accessed on 11 March 2025). Oral bioavailability OB ≥ 30%, DL ≥ 0.18, and high gastrointestinal (GI) absorption were used as screening criteria [44,47]. Components meeting all three criteria were considered potential drug-like compounds. These compounds were submitted to the SwissTargetPrediction server (probability ≥ 0.5) to predict protein targets [48]. The resulting targets were restricted to Homo sapiens and standardized using the STRING database (https://string-db.org/, accessed on 30 July 2025).

#### 4.2.2. Collection of Target Genes

To investigate the immune-mediated anti-breast cancer mechanisms of CS, related target genes were collected using the keywords “Breast Cancer” and “Immune System” from the GeneCards (https://www.genecards.org/, accessed on 10 March 2025), OMIM (https://www.omim.org/, accessed on 10 March 2025), and DisGeNET databases (https://www.disgenet.org/home/, accessed on 10 March 2025). To prioritize clinically relevant targets, we applied a relevance score threshold of ≥7.0 for GeneCards entries (scale 0–10) and retained only those with DisGeNET Disease Specificity Index ≥ 0.3. All available targets associated with these keywords were retrieved without additional filtering. Duplicates were removed, and the intersection of breast cancer and immune system-related genes was compiled in Excel. To obtain a comprehensive target dataset, database searches utilized generic terms “Breast Cancer” rather than subtype-specific keywords. This strategy ensures coverage of common oncogenic pathways but may mask subtype-specific immune regulatory mechanisms, representing a deliberate scope limitation of this exploratory study.

#### 4.2.3. Intersection of TCM Component Targets and Disease Targets

The jvenn online tool (https://www.bioinformatics.com.cn/static/others/jvenn/index.html, accessed on 30 July 2025) [49] was used to intersect target genes of CS chemical components, breast cancer-related genes, and immune system-related genes. A Venn diagram was generated to identify potential targets of CS in breast cancer pathways.

#### 4.2.4. Construction of Protein-Protein Interaction (PPI) Network

Intersecting targets from Section 4.2.3 were submitted to the STRING database (https://string-db.org/, accessed on 30 July 2025) with the species restricted to Homo sapiens and a high confidence score (≥0.900). The resulting PPI network was imported into Cytoscape 3.10.1 for visualization and analyzed using the CentiScaPe 2.2 plugin.

#### 4.2.5. GO Functional and KEGG Pathway Enrichment Analysis

Intersecting targets were uploaded to the Metascape database [50] for functional enrichment analysis. GO terms—including biological process (BP), molecular function (MF), and cellular component (CC)—and KEGG pathways were analyzed via the Bioinformatics online platform (http://www.bioinformatics.com.cn/, accessed on 31 July 2025).

Terms with *p* ≤ 0.05 were considered significant, and the top 10 entries based on enrichment percentage were selected.

#### 4.2.6. Molecular Docking Evaluation

Molecular docking simulations were conducted to predict binding affinities between prioritized CS compounds and core immune-oncology targets. The top 20 core targets were selected based on integrated centrality metrics calculated using CentiScaPe 2.2. Protein crystal structures were retrieved from the RCSB Protein Data Bank (PDB, https://www.rcsb.org/, accessed on 12 May 2025). Selection prioritized human protein complexes with native ligands where available: AKT1 (PDB: 3CQW), STAT3 (PDB: 6NJS), TP53 (PDB: 2IG0), and PIK3CA (PDB: 4JPS). Structure preparation involved removing heteroatoms (water, ions, co-crystallized ligands) in PyMOL 3.1, adding polar hydrogens via AutoDockTools 1.5.6, and converting to PDBQT format. Ligand preparation involved 3D structure generation from PubChem SDFs using OpenBabel 2.4.1.

#### 4.2.7. Molecular Dynamics Simulations

Molecular dynamics simulations: All-atom molecular dynamics (MD) simulations were carried out using GROMACS 2023.2 under periodic boundary conditions. The protein was described with the AMBER99SB-ILDN force field. Ligand topologies were generated using ACPYPE with GAFF parameters. Each complex was solvated in an explicit TIP3P water model using a dodecahedral periodic box, neutralized with counterions, and supplemented with NaCl to reach an ionic strength of 0.15 M.

Energy minimization was performed using the steepest-descent algorithm until the maximum force was below 1000 kJ·mol^−1^·nm^−1^. The systems were then equilibrated in two restrained stages: 100 ps NVT at 300 K, followed by 100 ps NPT at 1 bar. Production MD simulations were conducted for 100 ns with a 2 fs time step. Long-range electrostatics were treated using the particle mesh Ewald (PME) method, and all bonds involving hydrogen atoms were constrained using LINCS. Trajectories were saved every 10 ps (5000 steps).

Thermodynamic stability was monitored by time series of temperature, pressure, and density (reported in Supplementary Figure S1 and summarized in Table S6).

Trajectory preprocessing and structural analyses: Trajectory preprocessing and analyses were performed after removing periodic boundary artifacts and fitting structures to the protein backbone. System stability was assessed using the RMSD of the protein backbone and ligand RMSD (ligand aligned to the protein), while global compactness was monitored by the radius of gyration (Rg). Residue-level flexibility was quantified using per-residue Cα RMSF.

Protein–ligand association was evaluated using the protein–ligand center-of-mass (COM) distance and the minimum heavy-atom distance between protein and ligand. Protein–ligand contacts were computed as the fraction of analyzed frames in which any ligand heavy atom was within 4.0 Å of a residue heavy atom. Hydrogen bonds were identified using a geometric criterion of donor–acceptor distance ≤ 3.5 Å and donor–H–acceptor angle ≥ 150°, and occupancies were reported as the fraction of frames satisfying these criteria.

To reduce the influence of early equilibration, the first 10 ns were treated as an equilibration phase (shown as shading in QC plots). For quantitative statistics, we focused on the stable plateau window (30–100 ns) unless otherwise stated. For contact and hydrogen-bond occupancy analyses, frames were sampled with a fixed stride (stride = 50; *n* = 141 frames for the 30–100 ns window, as indicated in the figure captions).

MM/(P)BSA binding free energy calculations: Binding free energies were estimated using gmx_MMPBSA on snapshots extracted from the equilibrated trajectory window (30–100 ns) with a fixed sampling stride. Both MM/GBSA and MM/PBSA models were reported. Energy components (van der Waals, electrostatics, polar solvation, nonpolar solvation/surface term) and the total binding free energy were summarized. Uncertainties for MM/(P)BSA components were estimated using block-based standard error of the mean (block-SEM), as implemented in gmx_MMPBSA (reported in Table S10 and visualized in Figure S4C).

Principal component analysis and free energy landscape: Essential dynamics were characterized using principal component analysis (PCA) on Cα atomic coordinates from the equilibrated trajectory segment (30–100 ns). The free energy landscape (FEL) was projected onto the first two principal components (PC1–PC2) using *G* = −*k_B_T* ln*P*, where *P* is the normalized probability density. PCA projections and variance diagnostics (scree plot and cumulative explained variance) are reported in Supplementary Figure S4D–F, and the PC1–PC2 FEL of the representative system is shown in Figure 7D.

## 5. Conclusions

This study presents a comprehensive computational analysis that predicts that *Cynomorium songaricum* (CS) may modulate breast cancer immunity through a multi-component, multi-target network involving 84 drug-like compounds and 776 putative targets. Our in silico results suggest that CS constituents could potentially influence NF-κB and TGF-β signaling pathways, which are known to regulate TIME immunosuppression. Molecular docking supports the feasibility of direct interactions between prioritized compounds (Tomentogenin, Sesamolin) and core targets (AKT1, STAT3, TP53), but these predictions require experimental confirmation. To strengthen the plausibility of docking-derived hypotheses under explicit-solvent dynamics, we further performed 100 ns all-atom MD simulations and focused the main-text mechanistic validation on the representative SRC–Tomentogenin system. MD analyses supported a stable bound ensemble during the equilibrated window, with persistent pocket occupancy, consistent interaction signatures, favorable MM/(P)BSA binding energetics, and a concentrated low-energy basin on the PCA-based free energy landscape. Supporting and negative/inconclusive MD outcomes for additional systems were documented in the Supplementary Materials.

In summary, this work establishes a data-driven, experimentally falsifiable framework for investigating CS as a candidate immunomodulatory agent, while highlighting the necessity of integrating computational predictions with mechanistic and translational studies. Collectively, the integrated pipeline—from MS-based chemical profiling to network-level target inference, docking prioritization, and MD-based validation—provides a coherent rationale to prioritize Tomentogenin for follow-up experimental evaluation as a potential SRC-binding ligand within the proposed multi-target framework.

## Data Availability

The data and code used for the analysis in this article are available on GitHub (https://github.com/Tanlicie/Cynomorium). Data is contained within the article or Supplementary Materials.

## Supplementary Materials

The following supporting information can be downloaded at https://www.mdpi.com/article/10.3390/ph19020314/s1, Table S1: Identification of the chemical constituents in the CS using HPLC-Q Exactive-Orbitrap-MS/MS; Table S2: List of the potential drug-like compounds of CS; Table S3: Mean binding energies of CS drug-like compounds against selected breast-cancer-related proteins; Table S4: List of Mass Spectrometry Parameter Information; Table S5: MD stability summary across complexes; Table S6: Quality control (QC) summary statistics for MD production runs; Table S7: Top flexible residues from Cα RMSF analysis; Table S8: Top protein–ligand contact residues and occupancies; Table S9: Hydrogen-bond statistics and key H-bonding residues; Table S10: MM/(P)BSA binding free energy decomposition with block-SEM; Figure S1: Quality-control (QC) profiles for MD simulations of the SRC–Tomentogenin and SRC–Sesamolin complexes; Figure S2: Structural stability and interaction profile of the SRC–Sesamolin complex during MD (30–100 ns); Figure S3: TP53–Sesamolin shows insufficient MD stability (negative/inconclusive); Figure S4: Additional interaction and PCA diagnostics for SRC–Tomentogenin (30–100 ns).

## Abbreviations

The following abbreviations are used in this manuscript:

CS	*Cynomorium songaricum* Rupr.
DL	Drug-Likeness
GI	Gastrointestinal
GO	Gene Ontology
KEGG	Kyoto Encyclopedia of Genes and Genomes
OMIM	Online Mendelian Inheritance in Man
OB	Oral Bioavailability
PPI	Protein-Protein Interaction
RCSB PDB	RCSB Protein Data Bank
TCM	Traditional Chinese Medicine
TCMSP	Traditional Chinese Medicine Systems Pharmacology Database and Analysis Platform
TIME	Tumor Immune Microenvironment
TAMs	Tumor-Associated Macrophages
UHPLC-Q-Exactive Orbitrap MS/MS	Ultra-High-Performance Liquid Chromatography-Q Exactive Orbitrap Mass Spectrometry
