# Supplementary material for: Systematic Investigation of Tumor Immune Microenvironment Modulation by Cynomorium songaricum Against Breast Cancer Through Integrated Chemomics, Network Pharmacology and Molecular Docking
Source: Pharmaceuticals (Basel). 2026 Feb 13;19(2):314. doi: 10.3390/ph19020314 (PMC12944387; doi:10.3390/ph19020314)
Supplement: Supplementary file 1 [file pharmaceuticals-19-00314-s001.zip › pharmaceuticals-4057910-supplementary.pdf]

Table S1. Identification of the chemical constituents in the CS using HPLC-Q Exactive-Orbitrap-MS/MS.

This table summarizes the 1100 chemical constituents identified from CS, with each entry (No.) corresponding to a unique metabolite.

No.	Metabolites	Retention time (min)	Formula	m/z	Fragment Ions	Adducts	Ion mode	Categories
**M0001**	Glycolaldehyde dimer	0.866033	$C_4H_8O_4$	101.0243486	60.0044, 62.6985, 71.0138, 72.9811, 72.9931, 73.0295, 83.0086, 83.0138, 99.0088, 101.0243	M-H_2O-H	NEG	Others
**M0002**	Arabinitol	0.904983	$C_5H_{12}O_5$	197.0662478	155.0353, 163.2045, 179.0450, 183.4835, 196.8951, 196.9765, 197.0106, 197.0198, 197.0466, 197.0553	M-H, M+FA-H	NEG	Carbohydrates and Glycosides
**M0003**	Glucose	0.904983	$C_6H_{12}O_6$	179.0559363	161.0090, 161.0448, 163.0396, 178.8144, 178.9775, 178.9944, 179.0005, 179.0196, 179.0356, 179.0550	M-H, M+FA-H	NEG	Carbohydrates and Glycosides
**M0004**	Turanose	0.904983	$C_{12}H_{22}O_{11}$	387.1139237	258.9656, 263.0759, 281.0877, 287.3069, 290.0882, 341.1084, 386.8083, 387.0155, 387.1081, 387.1134	M+FA-H	NEG	Carbohydrates and Glycosides
**M0005**	L-Threonic acid	0.92405	$C_4H_8O_5$	135.0297095	89.0244, 93.7290, 100.4806, 116.9310, 117.0190, 118.7666, 120.1973, 121.5769, 134.8943, 135.0298	M-H	NEG	Carbohydrates and Glycosides
**M0006**	Maltopentaose	0.941217	$C_{30}H_{52}O_{26}$	827.2677459	161.0446, 179.0560, 191.0177, 215.9798, 323.0243, 341.1085, 485.1516, 503.1606, 665.2191, 827.2686	M-H, M+FA-H	NEG	Carbohydrates and Glycosides
**M0007**	Cytarabine	1.011067	$C_9H_{13}N_3O_5$	244.092133	209.0556, 209.0910, 215.9953, 226.0682, 226.1191, 226.1436, 227.1129, 233.0654, 243.9907, 244.0877	M+H, M+NH_4	POS	Carbohydrates and Glycosides
**M0008**	Melezitose	1.0832	$C_{18}H_{32}O_{16}$	527.1568925	393.0334, 407.1075, 413.0606, 422.0707, 430.0597, 437.1193, 466.1624, 467.1265, 479.0474, 527.1580	M+NH_4, M+Na	POS	Carbohydrates and Glycosides

**M0009**	1,1,1,1-Kestohexaose	1.153033	$C_{36}H_{62}O_{31}$	1035.32431	72.9703, 98.6079, 108.4044, 118.6611, 128.0347, 179.0547, 341.1069, 989.3132, 1035.2976	M+FA-H	NEG	Carbohydrates and Glycosides
**M0010**	1-beta-D-Arabinofuranosyluracil	1.252033	$C_9H_{12}N_2O_6$	243.0618399	199.1201, 200.0563, 208.1974, 208.2422, 208.7025, 209.3418, 225.0047, 225.0514, 243.0139, 243.0621	M-H, M+FA-H	NEG	Carbohydrates and Glycosides
**M0011**	Gallic acid	1.82345	$C_7H_6O_5$	169.0141282	127.0686, 127.6842, 134.5400, 147.1715, 155.2370, 166.6191, 168.0097, 168.8352, 168.8877, 169.0140	M-H, $_2$M-H	NEG	Phenols
**M0012**	Costunolide	10.44332	$C_{15}H_{20}O_2$	233.1532548	177.0908, 182.1090, 187.1478, 187.1681, 189.1640, 191.1063, 197.1320, 205.1586, 215.1427, 233.1532	M+H	POS	Terpenes
**M0013**	Peucedanocoumarin II	10.505	$C_{21}H_{22}O_7$	409.1247775	212.8672, 227.0695, 245.0803, 287.0902, 295.1633, 307.2804, 309.0719, 375.4015, 408.3693, 409.1244	M+NH_4, M+Na	POS	Phenylpropanoids
**M0014**	Oleanolic Acid	12.70553	$C_{30}H_{48}O_3$	457.3665013	320.8658, 373.0096, 411.3617, 439.3539, 446.1442, 457.2109, 457.2771, 457.2830, 457.3634, 457.3714	M+H	POS	Terpenes
**M0015**	Alanyleucine	2.92825	$C_9H_{18}N_2O_3$	201.1244967	172.8848, 174.7531, 183.0658, 200.8538, 200.8802, 200.9839, 201.0214, 201.0403, 201.0759, 201.1246	M-H	NEG	Amino Acids, Peptides and derivatives
**M0016**	Vanillic acid 4-beta-D-glucopyranoside	3.053017	$C_{14}H_{18}O_9$	329.0876244	209.0457, 221.0439, 239.0551, 260.9994, 269.0663, 285.0963, 293.1227, 312.1559, 328.9905, 329.0876	M-H, M+FA-H, $_2$M-H	NEG	Carbohydrates and Glycosides
**M0017**	Methyl deacetylasperulosidate	3.617483	$C_{17}H_{24}O_{11}$	449.1297859	223.0605, 240.8642, 241.0715, 266.1798, 305.1530, 332.1187, 371.1008, 376.2263, 403.1242, 449.1277	M+FA-H	NEG	Terpenes
**M0018**	3-O-Methylgallic acid	3.825367	$C_8H_8O_5$	183.0297886	138.9070, 139.0031, 139.0400, 139.0763, 149.0985, 165.0560, 168.0062, 171.2217, 183.0066, 183.0295	M-H	NEG	Phenols

**M0019**	Phlorigidoside C	3.845733	$C_{17}H_{24}O_{11}$	449.1298546	245.0816, 246.0856, 278.8473, 289.0715, 290.0748, 331.0811, 371.0985, 403.1242, 405.2129, 449.1298	M-H, M+FA-H	NEG	Terpenes
**M0020**	4-O-beta-Glucopyranosyl-cis-coumaric acid	3.866017	$C_{15}H_{18}O_8$	371.0980727	274.0744, 286.0454, 325.0925, 327.1067, 329.5043, 338.2959, 369.5916, 371.0550, 371.0971, 371.1021	M-H_2O-H, M+FA-H	NEG	Carbohydrates and Glycosides
**M0021**	Salidroside	3.9067	$C_{14}H_{20}O_7$	345.1187895	267.0979, 299.0136, 299.0352, 299.1131, 309.1556, 327.1670, 328.1506, 344.9678, 345.0789, 345.1181	M+FA-H	NEG	Carbohydrates and Glycosides
**M0022**	Atractyloside A	3.949733	$C_{21}H_{36}O_{10}$	493.2288903	305.1904, 368.0920, 373.0987, 378.2022, 406.1368, 442.1165, 447.1506, 447.2221, 493.1490, 493.2273	M+FA-H	NEG	Terpenes
**M0023**	Cichoriin	3.97005	$C_{15}H_{16}O_9$	339.071761	219.0302, 232.4141, 233.2951, 251.0938, 277.0720, 295.0432, 335.0554, 338.1171, 338.1626, 339.0719	M-H	NEG	Phenylpropanoids
**M0024**	Sesamoside	3.97005	$C_{17}H_{24}O_{12}$	419.1191233	215.9903, 221.0447, 239.0556, 257.0654, 305.1492, 343.1045, 357.1150, 401.1068, 415.2069, 419.1187	M-H, $_2$M-H	NEG	Terpenes
**M0025**	4-Aminocinnamic Acid	3.978417	$C_9H_9NO_2$	146.0600489	116.5138, 118.0652, 119.0435, 119.0495, 122.5978, 125.9441, 126.0439, 128.0495, 141.7158, 146.0599	M+H-H_2O	POS	Phenylpropanoids
**M0026**	Paeoniflorin sulfite	4.030417	$C_{23}H_{28}O_{13}S$	543.1174102	423.0759, 442.2468, 497.1092, 499.3251, 526.6864, 537.3448, 537.3566, 539.9216, 542.3787, 543.1171	M-H	NEG	Carbohydrates and Glycosides
**M0027**	gamma-Glu-Phe	4.139867	$C_{14}H_{18}N_2O_5$	295.1283852	232.0959, 241.0378, 249.1235, 249.1591, 277.1171, 277.1463, 278.1022, 278.1399, 295.0458, 295.1272	M+H	POS	Amino Acids, Peptides and derivatives
**M0028**	6-Hydroxykaempferol 3,6-diglucoside	4.139867	$C_{27}H_{30}O_{17}$	627.1544152	345.0579, 370.8834, 465.1018, 478.2158, 578.2653, 626.5491, 626.7907, 626.8068, 626.9745, 627.1527	M+H, M+Na	POS	Flavonoids

**M0029**	Catechin	4.201967	$C_{15}H_{14}O_6$	291.0857163	245.1277, 246.1227, 249.0755, 273.0736, 273.0803, 273.1227, 274.1082, 274.1156, 291.0503, 291.0876	M+H	POS	Flavonoids
**M0030**	Brevifolincarboxylic acid	4.25365	$C_{13}H_8O_8$	291.01456	247.2662, 247.3997, 248.8199, 273.0049, 273.0399, 288.7475, 290.0750, 290.1092, 290.1711, 291.0148	M-H	NEG	Phenylpropanoids
**M0031**	Kaempferol 3,7-Di-o-glucoside	4.274067	$C_{27}H_{30}O_{16}$	609.1464165	447.0928, 454.5800, 463.0916, 476.0949, 489.1057, 563.1816, 563.1982, 563.2794, 608.6400, 609.1451	M-H	NEG	Flavonoids
**M0032**	ClemastaninB	4.274067	$C_{32}H_{44}O_{16}$	729.2618885	400.3272, 417.4537, 509.7632, 521.2024, 558.8425, 637.2021, 641.9246, 683.2554, 729.1411, 729.1639	M+FA-H	NEG	Phenylpropanoids
**M0033**	Methyl 5-hydroxypyridine-2-carboxylate	4.284317	$C_7H_7NO_3$	154.0498497	136.1199, 137.0600, 137.0955, 137.1150, 139.9337, 140.0138, 140.0340, 140.1401, 154.0212, 154.0498	M+H, M+Na	POS	Alkaloids
**M0034**	Asperuloside	4.2945	$C_{18}H_{22}O_{11}$	459.1143767	337.0339, 353.0901, 357.0798, 378.0768, 385.0525, 397.0545, 413.0462, 413.1087, 458.2584, 459.1122	M+FA-H	NEG	Terpenes
**M0035**	Isorhamnetin 3,7-O-diglucoside	4.324567	$C_{28}H_{32}O_{17}$	641.1701697	347.0750, 359.0757, 461.1054, 465.1033, 473.1936, 479.1172, 509.1275, 640.8171, 640.8266, 641.1723	M+H, M+Na	POS	Flavonoids
**M0036**	Cis-Ferulic acid 4-O-beta-D-glucopyranoside	4.35425	$C_{16}H_{20}O_9$	355.102971	267.0640, 280.0797, 287.0656, 295.0823, 309.0980, 311.0560, 312.1091, 355.0446, 355.0968, 355.1030	M-H	NEG	Carbohydrates and Glycosides
**M0037**	Vicenin 2	4.3662	$C_{27}H_{30}O_{15}$	595.1646978	499.1228, 505.1137, 511.1227, 523.1225, 529.1339, 541.1332, 559.1439, 577.1542, 594.8013, 595.1646	M+H	POS	Flavonoids
**M0038**	Epicatechin	4.478283	$C_{15}H_{14}O_6$	289.0716544	227.0701, 245.0113, 245.0815, 245.1769, 247.0620, 271.0597, 271.1548, 289.0009, 289.0349, 289.0714	M-H	NEG	Flavonoids

**M0039**	Roseoside	4.498667	$C_{19}H_{30}O_8$	431.1919641	277.1445, 309.0977, 367.1758, 385.1873, 387.0612, 387.1162, 431.1007, 431.1083, 431.1332, 431.1937	M+FA-H	NEG	Terpenes
**M0040**	Quercetin 3-gentiobioside	4.55095	$C_{27}H_{30}O_{17}$	627.1545718	345.0584, 369.0597, 447.0913, 465.1017, 626.3828, 626.8054, 626.8163, 626.8286, 627.1578, 627.1694	M+H, M+Na	POS	Flavonoids
**M0041**	Vicenin 3	4.57055	$C_{26}H_{28}O_{14}$	565.1542875	469.1125, 475.1132, 481.1115, 493.1123, 499.1225, 511.1228, 529.1334, 547.1439, 564.7907, 565.1516	M+H	POS	Flavonoids
**M0042**	Albiflorin	4.6104	$C_{23}H_{28}O_{11}$	481.1694115	301.1063, 303.0496, 304.0532, 305.0647, 305.2572, 315.1216, 319.0447, 319.1168, 463.0851, 481.1686	M+H	POS	Terpenes
**M0043**	3-Feruloylquinic acid	4.6621	$C_{17}H_{20}O_9$	367.1030191	242.9895, 246.0114, 247.0617, 287.0564, 287.4121, 323.0764, 336.1659, 366.1857, 367.0087, 367.1031	M-H	NEG	Phenylpropanoids
**M0044**	Daidzin	4.6688	$C_{21}H_{20}O_9$	417.116908	286.1747, 300.1912, 302.0724, 302.1982, 324.1724, 354.1821, 374.7075, 388.2151, 400.1885, 417.1177	M+H	POS	Flavonoids
**M0045**	Paeoniflorin	4.6818	$C_{23}H_{28}O_{11}$	525.1610828	431.1343, 449.1447, 449.2082, 457.1712, 479.0400, 479.0749, 479.1548, 525.0315, 525.1544, 525.1620	M+FA-H, $_2$M-H, M-H	NEG	Terpenes
**M0046**	2-(3,4-dihydroxyphenyl)-7-(beta-D-glucopyranosyloxy)-8-hydroxy-4H-1-benzopyran-4-one	4.68905	$C_{21}H_{20}O_{11}$	449.1069062	355.1064, 383.0748, 391.1935, 391.2079, 395.0782, 413.0840, 431.0938, 431.1904, 449.1070, 449.1196	M+H	POS	Flavonoids
**M0047**	Fraxetin	4.701583	$C_{10}H_8O_5$	207.0297096	164.0717, 166.9929, 175.3791, 179.0340, 182.8791, 190.7921, 192.0063, 206.9201, 206.9954, 207.0296	M-H	NEG	Phenylpropanoids
**M0048**	Isoviolanthin	4.701583	$C_{27}H_{30}O_{14}$	577.156077	397.0956, 413.0859, 425.0880, 439.1040, 457.1130, 473.1078, 487.1247, 503.1202, 576.2726, 577.1556	M-H	NEG	Flavonoids

**M0049**	Quercetin 3-sambubioside	4.727383	$C_{26}H_{28}O_{16}$	597.1440424	531.7977, 539.2693, 549.0521, 553.2928, 563.7887, 591.6082, 596.3309, 596.5695, 596.8204, 597.1548	M+H	POS	Flavonoids
**M0050**	Quercetin-3-O-D-glucosyl]-(1-2)-L-rhamnoside	4.7616	$C_{27}H_{30}O_{16}$	609.1462617	447.1011, 447.1116, 563.0794, 563.1773, 563.2766, 602.3082, 608.2672, 608.2836, 608.2955, 609.1454	M-H	NEG	Flavonoids
**M0051**	Rutin	4.766817	$C_{27}H_{30}O_{16}$	611.1595515	449.1066, 465.1018, 465.1815, 500.1460, 610.3297, 610.8110, 610.8290, 611.1687, 611.1778, 611.1894	M+H, M+Na	POS	Flavonoids
**M0052**	Apigenin 5-O-glucoside	4.843383	$C_{21}H_{20}O_{10}$	431.0979807	323.0561, 335.0555, 341.0663, 367.1754, 371.0786, 385.1834, 385.1888, 413.0905, 413.1819, 431.0970	M-H	NEG	Flavonoids
**M0053**	Liquiritin	4.902817	$C_{21}H_{22}O_9$	436.1589974	274.0782, 408.0006, 408.6402, 418.1531, 418.1592, 419.1315, 436.0921, 436.1479, 436.1569, 436.1624	M+Na, M+K, $M+NH_4$	POS	Flavonoids
**M0054**	Luteolin 7-O-glucuronide	4.902817	$C_{21}H_{18}O_{12}$	463.0861271	287.5316, 288.2752, 288.3061, 289.2815, 289.3139, 301.0698, 305.2538, 326.6401, 448.5847, 463.0858	M+H	POS	Flavonoids
**M0055**	Dactylorhin A	4.9237	$C_{40}H_{56}O_{22}$	933.3256717	629.8809, 631.1691, 692.8309, 707.2565, 710.2444, 714.8078, 887.3185, 932.9285, 933.2015, 933.2318	M-H, M+FA-H	NEG	Carbohydrates and Glycosides
**M0056**	Astilbin	4.960817	$C_{21}H_{22}O_{11}$	433.1119592	320.9175, 323.0895, 329.1011, 337.0684, 367.0822, 379.0811, 397.0922, 415.1005, 415.1948, 433.1116	$M+H-H_2O$	POS	Flavonoids
**M0057**	Isovanillin	4.98065	$C_8H_8O_3$	153.0544767	130.0161, 134.0598, 135.0436, 135.0803, 135.1168, 136.0752, 140.0340, 143.0337, 153.0180, 153.0543	M+H	POS	Phenols
**M0058**	Neoliquiritin	5.0009	$C_{21}H_{22}O_9$	419.1326228	305.2050, 315.0840, 330.1088, 369.1333, 383.1519, 401.1249, 401.1582, 401.1811, 419.1226, 419.1293	M+H	POS	Flavonoids

**M0059**	Trifolin	5.020233	$C_{21}H_{20}O_{11}$	449.1070301	303.0097, 303.0485, 317.0651, 359.1483, 391.1929, 413.1250, 431.0954, 431.1955, 448.7341, 449.1090	M+H	POS	Flavonoids
**M0060**	Isoferulic acid	5.084733	$C_{10}H_{10}O_4$	193.0504785	152.9968, 158.6838, 161.0464, 165.0196, 165.0556, 172.9337, 173.0056, 178.0270, 192.9979, 193.0505	M-H	NEG	Phenylpropanoids
**M0061**	Syringaldehyde	5.09965	$C_9H_{10}O_4$	183.0649867	147.1171, 154.9901, 155.0701, 159.9691, 165.0548, 165.0909, 165.1274, 166.0981, 182.9847, 183.0651	M+H	POS	Phenols
**M0062**	Acanthoside B	5.105167	$C_{28}H_{36}O_{13}$	579.208356	443.0925, 459.1263, 471.0086, 489.1423, 533.1683, 533.2585, 535.1096, 579.1480, 579.1715, 579.2014	M-H, M+FA-H	NEG	Phenylpropanoids
**M0063**	Azaleatin	5.160017	$C_{16}H_{12}O_7$	317.0646374	286.0444, 287.1990, 288.0609, 299.0551, 299.0925, 299.2001, 302.0412, 316.0533, 316.9949, 317.0647	M+H	POS	Flavonoids
**M0064**	Isorhamnetin-3-O-glucoside	5.160017	$C_{22}H_{22}O_{12}$	479.1176933	305.2019, 317.0647, 318.0304, 318.1125, 332.1357, 333.0583, 340.2497, 347.0760, 350.1408, 479.0771	M+H	POS	Flavonoids
**M0065**	Isochlorogenic acid C	5.186017	$C_{25}H_{24}O_{12}$	515.118374	353.0872, 417.1180, 447.0929, 449.1467, 457.1691, 469.0736, 479.1551, 514.2696, 514.2794, 515.1217	M-H	NEG	Phenylpropanoids
**M0066**	Apigenin-7-glucuronide	5.201533	$C_{21}H_{18}O_{11}$	447.0911029	309.0237, 313.0692, 327.0366, 355.0841, 361.0263, 412.1667, 429.0821, 430.2353, 446.6710, 447.0908	M+H	POS	Flavonoids
**M0067**	Choerospondin	5.228367	$C_{21}H_{22}O_{10}$	433.1141006	299.9910, 300.0266, 301.0336, 305.1481, 313.0681, 387.2022, 389.1255, 432.1923, 432.2607, 433.1143	M-H	NEG	Flavonoids
**M0068**	DiosMetin 7-O-beta-D-Glucuronide	5.2486	$C_{22}H_{20}O_{12}$	475.0878764	311.0555, 313.0696, 317.0257, 395.0411, 431.0973, 474.1573, 474.1645, 474.2051, 474.2241, 475.0820	M-H	NEG	Flavonoids

**M0069**	Iristectorin B	5.260967	$C_{23}H_{24}O_{12}$	493.1327236	331.0803, 332.5886, 350.2024, 365.0744, 373.0542, 409.7239, 425.0963, 475.1228, 475.2774, 493.1341	M+H	POS	Flavonoids
**M0070**	Quercetin-3-O-[2-O-(6-O-p-hydroxyl-E-coumaroyl)-D-glucosyl]-(1-2)-L-rhamnoside	5.308883	$C_{36}H_{36}O_{18}$	755.1836025	341.8848, 457.1748, 515.1121, 531.5920, 577.2524, 593.1492, 609.1460, 688.9510, 754.2968, 755.1820	M-H	NEG	Flavonoids
**M0071**	Isoliquiritin apioside	5.361633	$C_{26}H_{30}O_{13}$	551.1746015	257.0801, 258.1144, 277.0921, 299.0903, 371.1823, 389.1212, 419.1324, 419.1993, 551.1639, 551.1718	M+H	POS	Flavonoids
**M0072**	Emodin-1-O-beta-D-glucopyranoside	5.410867	$C_{21}H_{20}O_{10}$	431.0980385	283.0614, 284.0325, 285.0392, 293.0446, 311.0552, 318.9502, 362.9409, 363.0154, 385.1861, 431.0979	M-H	NEG	Quinones
**M0073**	Ginsenoside Rg1	5.430967	$C_{42}H_{72}O_{14}$	845.4915316	637.4327, 637.5614, 679.4460, 799.4855, 806.7783, 843.4766, 845.2151, 845.2304, 845.2822, 845.4911	M+FA-H	NEG	Terpenes
**M0074**	Isoliquiritin	5.460883	$C_{21}H_{22}O_9$	419.13254	258.1145, 269.0805, 287.0536, 330.1081, 383.1097, 401.1172, 401.1585, 419.1244, 419.1310, 419.1389	M+H-H_2O, M+H	POS	Flavonoids
**M0075**	Luteolin-3-O-beta-D-glucuronide	5.4808	$C_{21}H_{18}O_{12}$	463.0859812	288.6735, 301.0699, 317.1949, 338.2550, 357.1143, 379.1592, 428.1249, 460.5921, 462.2661, 463.0811	M+H	POS	Flavonoids
**M0076**	Militarine	5.530817	$C_{34}H_{46}O_{17}$	771.2726722	457.7638, 461.6967, 538.6449, 573.1114, 585.4307, 588.7778, 638.5861, 725.2664, 771.2219, 771.2778	M-H, M+FA-H	NEG	Carbohydrates and Glycosides
**M0077**	Baicalin	5.540483	$C_{21}H_{18}O_{11}$	447.0910178	335.0938, 335.1532, 361.0296, 429.0820, 429.2324, 430.2316, 446.2561, 446.2643, 446.3802, 447.0912	M+H	POS	Flavonoids
**M0078**	Lactiflorin	5.551367	$C_{23}H_{26}O_{10}$	507.1504588	461.2014, 461.2089, 461.2653, 463.0934, 485.0070, 506.2391, 506.2620, 506.2701, 507.1445, 507.1524	M+FA-H	NEG	Terpenes

**M0079**	Ononin	5.631933	$C_{22}H_{22}O_9$	475.1242975	305.1893, 311.0564, 312.0643, 313.0719, 317.0299, 377.6456, 417.1233, 429.1185, 474.2299, 475.1223	M+FA-H	NEG	Flavonoids
**M0080**	Ponicidin	5.639267	$C_{20}H_{26}O_6$	345.1681251	271.1684, 281.1527, 299.0558, 299.1633, 309.1476, 317.1741, 327.0885, 327.1592, 345.0643, 345.1695	M+H-H_2O	POS	Terpenes
**M0081**	N-Feruloyltyramine	5.924167	$C_{18}H_{19}NO_4$	314.1379183	279.1584, 283.0958, 286.0715, 291.0228, 297.0373, 297.1208, 297.1529, 299.2168, 305.2488, 314.1380	M+H	POS	Phenylpropanoids
**M0082**	4'-Demethylepipodophyllotoxin	5.967	$C_{21}H_{20}O_8$	401.1218419	293.0753, 315.0813, 319.0965, 330.1086, 331.1700, 341.1004, 347.0847, 351.1210, 383.1070, 401.1167	M+H	POS	Phenylpropanoids
**M0083**	Liquiritigenin	6.0222	$C_{15}H_{12}O_4$	255.0662563	214.9930, 226.4116, 237.1129, 237.1499, 243.1648, 253.2045, 254.0327, 254.8074, 254.8240, 255.0658	M-H, $_2$M-H	NEG	Flavonoids
**M0084**	Oroxylin A-7-O-glucuronide	6.0222	$C_{22}H_{20}O_{11}$	459.0933487	266.0269, 266.0580, 268.0375, 278.7280, 283.0610, 295.0600, 343.2130, 351.5491, 413.2169, 459.0919	M-H	NEG	Flavonoids
**M0085**	Chrysin-7-O-glucuronide	6.068133	$C_{21}H_{18}O_{10}$	431.0966218	208.4745, 208.9528, 210.9485, 255.0645, 268.0700, 269.0798, 269.1156, 287.0506, 320.9181, 354.3539	M+H	POS	Flavonoids
**M0086**	Nepetin	6.206317	$C_{16}H_{12}O_7$	315.0508303	300.0272, 300.3446, 302.4261, 305.1866, 312.4998, 314.2044, 314.6895, 314.7385, 314.9828, 315.0508	M-H	NEG	Flavonoids
**M0087**	Wogonoside	6.251067	$C_{22}H_{20}O_{11}$	461.1066796	286.2848, 299.0276, 299.0886, 305.2468, 348.4344, 353.5821, 398.2102, 443.0875, 444.2118, 461.1058	M+H	POS	Flavonoids
**M0088**	Syringaresinol	6.2713	$C_{22}H_{26}O_8$	401.1583921	353.1013, 356.1259, 360.0016, 368.1234, 369.1307, 371.1471, 373.1611, 383.1482, 385.1904, 401.1573	M+H-H_2O, M+H	POS	Phenylpropanoids

**M0089**	Bupleuroside XIII	6.287967	$C_{42}H_{70}O_{14}$	843.475925	353.7657, 354.9701, 561.9724, 625.3747, 635.4166, 797.4692, 843.3551, 843.3696, 843.4454, 843.4675	M+FA-H	NEG	Terpenes
**M0090**	Indigotin	6.348833	$C_{16}H_{10}N_2O_2$	307.0722322	263.8412, 266.9870, 279.0659, 286.9928, 289.0514, 289.1798, 305.1510, 306.2065, 307.0208, 307.0698	M+FA-H	NEG	Alkaloids
**M0091**	Flazin	6.353367	$C_{17}H_{12}N_2O_4$	309.0862923	217.1591, 235.0850, 255.1742, 263.0809, 263.2006, 273.1844, 281.0914, 291.1572, 291.1914, 309.0865	M+H, M+Na	POS	Alkaloids
**M0092**	(+)-Balanophonin	6.475883	$C_{20}H_{20}O_6$	357.1321714	321.1113, 321.1566, 325.1051, 327.1214, 339.1219, 339.1735, 341.1808, 357.0559, 357.0912, 357.1327	M+H	POS	Phenylpropanoids
**M0093**	Pinoresinol	6.496233	$C_{20}H_{22}O_6$	341.1374871	295.0969, 296.1048, 308.1036, 309.1105, 311.1282, 313.1419, 323.0893, 323.1268, 323.1852, 341.1405	M+H-H_2O	POS	Phenylpropanoids
**M0094**	Hedysarimcoumestan B	6.558333	$C_{16}H_{10}O_6$	297.0403555	253.1446, 254.0222, 269.0714, 271.0600, 276.9879, 279.1236, 279.1584, 282.0168, 296.6166, 297.0395	M-H	NEG	Flavonoids
**M0095**	Benzoylpaeoniflorin	6.558333	$C_{30}H_{32}O_{12}$	629.1880511	537.2523, 553.0710, 553.1736, 553.2753, 565.1700, 583.1808, 583.2624, 583.2898, 629.1769, 629.1887	M+FA-H	NEG	Terpenes
**M0096**	Saikosaponin S	6.64195	$C_{48}H_{78}O_{18}$	987.5189793	72.3526, 89.0243, 101.0241, 113.0240, 115.3437, 145.1139, 452.2203, 688.8043, 941.5112, 987.5281	M+FA-H	NEG	Terpenes
**M0097**	Benzoylalbiflorin	6.67985	$C_{30}H_{32}O_{12}$	585.1954951	301.0704, 301.1066, 301.1476, 305.2584, 319.1169, 319.1623, 371.1099, 430.2631, 445.1038, 585.1885	M+H	POS	Carbohydrates and Glycosides
**M0098**	Cinnamic acid	6.761283	$C_9H_8O_2$	131.0491097	107.4763, 108.6928, 110.0239, 116.4813, 117.1088, 118.5156, 118.7423, 121.6054, 129.0077, 131.0492	M+H-H_2O	POS	Phenylpropanoids

**M0099**	Naringenin chalcone	6.782733	$C_{15}H_{12}O_5$	273.0749722	225.6989, 227.1783, 231.0644, 255.0658, 255.1736, 255.2067, 263.6432, 264.1470, 272.0688, 273.0751	M+H	POS	Flavonoids
**M0100**	Hispidulin	6.848517	$C_{16}H_{12}O_6$	299.0557444	284.0322, 284.3258, 284.5018, 285.2539, 286.2443, 296.6966, 298.7594, 298.9922, 299.0172, 299.0556	M-H, $_{2}$M-H	NEG	Flavonoids
**M0101**	Physcion 1-O-beta-D-glucoside	6.890517	$C_{22}H_{22}O_{10}$	491.1194756	410.1921, 423.2753, 445.1127, 446.2264, 485.3254, 490.2804, 491.0095, 491.0159, 491.0407, 491.0926	M-H, M+FA-H	NEG	Quinones
**M0102**	Iristectorigenin A	7.003883	$C_{17}H_{14}O_7$	331.0803159	311.6145, 313.0286, 313.1415, 313.1792, 313.2353, 315.0108, 315.0489, 316.0567, 320.6190, 331.0803	M+H	POS	Flavonoids
**M0103**	Isorhamnetin	7.1601	$C_{16}H_{12}O_7$	315.0508857	270.3465, 271.0228, 272.0309, 274.9926, 278.4527, 283.0243, 300.0272, 314.2032, 315.0129, 315.0508	M-H	NEG	Flavonoids
**M0104**	Ginsenoside Rh1	7.352883	$C_{36}H_{62}O_9$	621.4348679	219.1749, 219.2105, 221.1894, 231.1741, 245.2255, 249.1859, 405.3505, 423.3611, 441.3725, 621.4364	M+H-H_2O	POS	Terpenes
**M0105**	Saikosaponin C	7.346533	$C_{48}H_{78}O_{17}$	971.5232974	366.0407, 392.4619, 410.2631, 462.4070, 617.4064, 763.4644, 779.4617, 903.3410, 925.5167, 971.5214	M+FA-H	NEG	Terpenes
**M0106**	Licorice saponin G2	7.392683	$C_{42}H_{62}O_{17}$	839.4045452	423.3264, 433.3126, 439.3206, 451.3197, 469.3307, 487.3409, 627.3492, 645.3636, 663.3732, 839.4080	M+H, M+Na, M+K	POS	Terpenes
**M0107**	Saikosaponin F	7.46935	$C_{48}H_{80}O_{17}$	973.5379155	379.0009, 454.7976, 457.3714, 765.4793, 781.4821, 888.2036, 926.5210, 927.5322, 972.9922, 973.5391	M+FA-H	NEG	Terpenes
**M0108**	Mogroside I E1	7.510117	$C_{36}H_{62}O_9$	683.4381124	503.5789, 505.6856, 527.1445, 590.9783, 615.1969, 637.2047, 637.4203, 637.4319, 638.1903, 683.4396	M+FA-H	NEG	Terpenes

**M0109**	Isoliquiritigenin	7.515167	$C_{15}H_{12}O_4$	257.0802676	237.0687, 239.0694, 239.1792, 240.0750, 240.1942, 242.0568, 246.0721, 256.2649, 256.2970, 257.0800	M+H	POS	Flavonoids
**M0110**	Saikosaponin H	7.6346	$C_{48}H_{78}O_{17}$	971.5232839	704.2693, 763.4627, 765.4807, 779.4585, 781.4791, 925.5166, 926.5230, 927.5303, 971.3116, 971.5223	M+FA-H	NEG	Terpenes
**M0111**	Batatasin III	7.662133	$C_{15}H_{16}O_3$	245.1167976	208.3680, 211.3324, 213.0903, 223.0044, 224.1287, 227.1074, 227.1827, 245.0584, 245.0641, 245.1167	M+H	POS	Phenylpropanoids
**M0112**	Alizarin 1-methyl ether	7.78705	$C_{15}H_{10}O_4$	255.0646285	231.6022, 232.9299, 237.0554, 237.1463, 237.1853, 239.0331, 240.0410, 240.0703, 254.8121, 255.0645	M+H	POS	Quinones
**M0113**	Ginsenoside Rd	7.8213	$C_{48}H_{82}O_{18}$	991.5492419	553.2101, 596.2229, 757.0824, 783.4866, 790.2735, 865.3585, 923.3271, 945.5435, 960.5717, 991.5507	M+FA-H	NEG	Terpenes
**M0114**	Glycyrrhizic acid	7.8683	$C_{42}H_{62}O_{16}$	823.4101875	357.2396, 369.2427, 383.2570, 389.3193, 407.3311, 435.3242, 453.3351, 471.3461, 647.3785, 823.4202	M+H, M+Na, M+K, $M+NH_4$	POS	Terpenes
**M0115**	Dihydroactinidiolide	7.988867	$C_{11}H_{16}O_2$	181.1220346	139.0751, 140.9508, 145.1006, 153.1272, 157.9735, 163.0754, 163.1115, 163.1475, 181.0486, 181.1219	M+H	POS	Terpenes
**M0116**	Hydnocarpin	8.093783	$C_{25}H_{20}O_9$	463.1038724	283.0245, 287.0544, 305.1464, 305.1866, 317.1016, 395.2034, 433.0905, 463.0045, 463.0364, 463.1041	M-H	NEG	Flavonoids
**M0117**	Glicoricone	8.214383	$C_{21}H_{20}O_6$	369.1322338	285.0745, 295.0614, 298.0468, 301.0694, 313.0698, 313.1149, 329.3421, 333.2083, 351.1196, 369.1313	M+H, M+Na	POS	Flavonoids
**M0118**	Aloe emodin	8.240317	$C_{15}H_{10}O_5$	269.0454969	254.0231, 257.0444, 267.0320, 267.9355, 268.0375, 268.1998, 268.8003, 268.9842, 269.0092, 269.0454	M-H	NEG	Quinones

**M0119**	Buddlejasaponin IVb	8.260817	$C_{48}H_{78}O_{18}$	987.5187388	766.1032, 779.4564, 815.3508, 851.3525, 941.3087, 941.5116, 945.2810, 969.5936, 987.4985, 987.5215	M+FA-H	NEG	Terpenes
**M0120**	Licorice-saponin H2	8.336617	$C_{42}H_{62}O_{16}$	845.391674	511.3323, 529.3386, 534.3557, 535.3340, 623.9980, 669.3604, 669.4819, 669.4972, 804.6132, 845.3922	M+Na, M+K, M+NH_4	POS	Terpenes
**M0121**	Eupatorin	8.34545	$C_{18}H_{16}O_7$	343.0820967	325.2028, 327.0488, 328.0127, 328.0580, 342.1954, 342.9807, 342.9973, 343.0075, 343.0481, 343.0818	M-H	NEG	Flavonoids
**M0122**	Diphyllin	8.386533	$C_{21}H_{16}O_7$	379.0822363	333.2001, 334.0849, 338.9869, 352.0583, 357.9581, 358.9905, 359.0031, 366.0741, 378.9992, 379.0816	M-H	NEG	Phenylpropanoids
**M0123**	Tridecanedioic acid	8.511483	$C_{13}H_{24}O_4$	243.1601132	215.0352, 222.9796, 223.0007, 223.0206, 225.0181, 225.1494, 242.2011, 242.9882, 243.0293, 243.1596	M-H	NEG	Others
**M0124**	Wogonin	8.70835	$C_{16}H_{12}O_5$	285.0749975	210.0980, 224.8198, 238.0975, 240.2318, 249.1850, 252.0442, 253.0842, 270.0515, 275.6778, 285.0749	M+H	POS	Flavonoids
**M0125**	3-Oxocholic acid	8.8513	$C_{24}H_{38}O_5$	424.304663	301.2162, 317.2257, 335.1628, 335.2350, 353.2468, 370.5544, 371.2571, 389.2697, 424.2355, 424.3055	M+NH_4	POS	Steroids
**M0126**	Saikosaponin G	8.991467	$C_{42}H_{68}O_{13}$	803.4541518	552.9879, 641.1174, 641.4036, 711.5046, 773.3954, 773.4140, 773.4503, 785.5853, 803.2862, 803.4545	M+H, M+NH_4, M+Na, M+H-H_2O	POS	Terpenes
**M0127**	Oroxylin A	9.033267	$C_{16}H_{12}O_5$	285.0750591	244.6372, 249.1844, 253.0853, 253.6412, 265.1520, 267.0639, 270.0517, 274.1541, 284.2939, 285.0753	M+H	POS	Flavonoids
**M0128**	Neobavaisoflavone	9.134483	$C_{20}H_{18}O_4$	367.1184637	346.9913, 346.9968, 351.0872, 352.0952, 366.2333, 366.9897, 366.9985, 367.0049, 367.0321, 367.1182	M+FA-H	NEG	Flavonoids

**M0129**	Ginsenoside F4	9.1985	$C_{42}H_{70}O_{12}$	784.519433	405.3511, 415.0316, 423.3614, 425.3716, 441.3727, 530.7635, 551.4084, 569.4230, 587.4280, 720.1465	M+NH$_4$, M+Na	POS	Terpenes
**M0130**	Prosaikogenin D	9.32245	$C_{36}H_{58}O_8$	663.4120632	264.0984, 270.9503, 434.2676, 486.1341, 559.3199, 595.2713, 617.4085, 663.0306, 663.0520, 663.4131	M+FA-H	NEG	Terpenes
**M0131**	Atractylenolide III	9.4076	$C_{15}H_{20}O_3$	231.1375956	187.1118, 187.1485, 189.0908, 189.1275, 195.1161, 203.1071, 203.1428, 213.1272, 231.0839, 231.1378	M+H-H$_2$O, M+H	POS	Terpenes
**M0132**	5-Desmethylsinensetin	9.48945	$C_{19}H_{18}O_7$	359.1115472	329.0635, 341.2081, 341.2816, 342.1837, 343.0799, 343.1036, 344.0871, 358.2777, 358.2834, 359.1116	M+H, M+Na	POS	Flavonoids
**M0133**	6''-O-Acetylsaikosaponin A	9.509867	$C_{44}H_{70}O_{14}$	867.4759744	661.4103, 761.2962, 761.4489, 779.2957, 779.4583, 810.7820, 821.4699, 832.3881, 832.4124, 867.4747	M-H, M+FA-H	NEG	Terpenes
**M0134**	Ginsenoside Rk3	9.53265	$C_{36}H_{60}O_8$	665.4277102	448.0459, 516.2517, 520.9790, 576.7100, 619.4222, 645.0338, 665.0261, 665.0422, 665.3124, 665.4274	M+FA-H	NEG	Terpenes
**M0135**	Saikosaponin E	9.573767	$C_{42}H_{68}O_{12}$	809.4706355	259.5773, 268.9281, 290.4859, 305.1870, 613.5776, 694.1586, 701.6146, 731.8188, 763.4653, 809.4701	M+FA-H	NEG	Terpenes
**M0136**	Licoisoflavone A	9.6347	$C_{20}H_{18}O_6$	355.1167337	299.0548, 299.0883, 309.1124, 319.2414, 327.1220, 337.1060, 337.2512, 354.6587, 355.0714, 355.1166	M+H	POS	Flavonoids
**M0137**	Licoricone	9.717517	$C_{22}H_{22}O_6$	381.1341891	340.9949, 351.0868, 360.9914, 360.9969, 364.8168, 365.1028, 366.1111, 380.9938, 381.0065, 381.1339	M-H	NEG	Flavonoids
**M0138**	Caulophyllogenin	9.819283	$C_{30}H_{48}O_5$	471.3458066	425.3404, 434.8266, 435.3239, 453.2789, 453.2958, 453.3314, 453.3420, 471.1039, 471.2879, 471.3399	M+H-H$_2$O, M+H	POS	Terpenes

**M0139**	Gypenoside LXXV	9.822533	$C_{42}H_{72}O_{13}$	829.4968325	537.3491, 550.4901, 564.1989, 621.4390, 773.5213, 783.4904, 793.2899, 793.6583, 829.0542, 829.5007	M-H, M+FA-H	NEG	Terpenes
**M0140**	Galactose 1-phosphate	0.804017	$C_6H_{13}O_9P$	259.0220251	205.0136, 212.4623, 223.0025, 229.7240, 230.8706, 237.8618, 241.0119, 244.1517, 258.8468, 259.0175	M-H	NEG	Carbohydrates and Glycosides
**M0141**	N6,N6,N6-Trimethyl-L-lysine	0.81535	$C_9H_{20}N_2O_2$	189.1594846	161.0294, 163.0388, 169.0525, 170.0292, 171.1235, 172.1077, 185.0295, 189.0234, 189.1344, 189.1593	M+H	POS	Amino Acids, Peptides and derivatives
**M0142**	L-Histidine	0.8337	$C_6H_9N_3O_2$	154.0620181	108.0567, 109.0406, 110.0722, 112.3894, 118.0411, 126.9035, 136.0513, 137.0354, 153.9986, 154.0621	M-H	NEG	Amino Acids, Peptides and derivatives
**M0143**	L-Arginine	0.851167	$C_6H_{14}N_4O_2$	173.1041914	130.9954, 131.0825, 131.1756, 131.4648, 144.9809, 156.0777, 172.8303, 172.8810, 172.9751, 173.1042	M-H	NEG	Amino Acids, Peptides and derivatives
**M0144**	Tagatose	0.92405	$C_6H_{12}O_6$	161.0453445	143.0336, 159.0298, 160.8414, 160.8923, 160.9354, 160.9490, 160.9757, 161.0089, 161.0232, 161.0450	M-H_2O-H	NEG	Carbohydrates and Glycosides
**M0145**	Gluconic acid	0.92405	$C_6H_{12}O_7$	195.0505795	152.0351, 159.0286, 160.0332, 165.0540, 177.0170, 177.0403, 178.0436, 194.9011, 195.0297, 195.0497	M-H	NEG	Carbohydrates and Glycosides
**M0146**	Homo-L-arginine	0.94285	$C_7H_{16}N_4O_2$	189.1342062	147.0136, 158.0921, 159.0134, 161.0293, 169.0525, 171.1242, 172.1081, 172.1329, 185.0286, 189.1345	M+H	POS	Amino Acids, Peptides and derivatives
**M0147**	Malic acid	0.957183	$C_4H_6O_5$	133.0140665	115.0479, 119.5722, 121.5856, 122.8690, 128.7300, 129.2692, 129.3541, 132.8676, 133.0034, 133.0141	M-H_2O-H, M-H	NEG	Organic acids and derivatives
**M0148**	Glucosamine	0.962317	$C_6H_{13}NO_5$	162.0757822	120.0651, 123.0299, 126.0552, 127.0390, 128.0225, 133.0277, 144.0654, 145.0487, 149.0115, 162.0759	M+H-H_2O	POS	Carbohydrates and Glycosides

**M0149**	Citric acid	0.994317	$C_6H_8O_7$	191.0195301	133.8689, 135.6821, 140.8574, 145.5006, 146.9384, 147.0301, 154.9984, 173.0090, 187.4984, 191.0197	M-H_2O-H, M-H	NEG	Organic acids and derivatives
**M0150**	L-Methionine	1.1949	$C_5H_{11}NO_2S$	150.0580772	123.0805, 126.0546, 128.0195, 132.0637, 132.0810, 133.0317, 135.0678, 146.0299, 150.0268, 150.0562	M+H	POS	Amino Acids, Peptides and derivatives
**M0151**	Cyclic AMP	1.191833	$C_{10}H_{12}N_5O_6P$	328.0447114	156.9462, 157.2582, 181.2719, 188.2451, 191.0197, 192.0238, 267.1108, 307.2228, 327.9990, 328.0448	M-H	NEG	Nucleotides and derivatives
**M0152**	3-Hydroxypicolinic acid	1.272283	$C_6H_5NO_3$	138.0195624	113.1785, 114.3696, 116.3962, 120.9859, 127.7408, 132.6692, 135.0832, 137.8915, 138.0100, 138.0195	M-H	NEG	Pyridines and derivatives
**M0153**	N-Acetyl-L-glutamic acid	1.292683	$C_7H_{11}NO_5$	188.0566763	144.0665, 144.1027, 146.0456, 159.8930, 161.8685, 170.0457, 170.0813, 188.0271, 188.0345, 188.0562	M-H	NEG	Amino Acids, Peptides and derivatives
**M0154**	2-Methylcitric acid	1.3112	$C_7H_{10}O_7$	187.0249592	144.1031, 146.0453, 158.8931, 169.0138, 169.0508, 170.0461, 186.8549, 186.9288, 187.0066, 187.0246	M-H_2O-H	NEG	Organic acids and derivatives
**M0155**	Serylvaline	1.3056	$C_8H_{16}N_2O_4$	205.1178628	160.0605, 169.0490, 174.0170, 187.0596, 187.1075, 188.0905, 205.0483, 205.0670, 205.0937, 205.1178	M+H	POS	Amino Acids, Peptides and derivatives
**M0156**	Valylalanine	1.380333	$C_8H_{16}N_2O_3$	189.1229701	171.0293, 171.0473, 171.1122, 172.0602, 172.0956, 189.0076, 189.0527, 189.0702, 189.0914, 189.1249	M+H	POS	Amino Acids, Peptides and derivatives
**M0157**	Adenosine	1.380333	$C_{10}H_{13}N_5O_4$	268.103264	191.0809, 197.0549, 209.0918, 211.1550, 223.1071, 232.7988, 234.0871, 250.1104, 251.1464, 268.1029	M+H	POS	Nucleotides and derivatives
**M0158**	Lysylleucine	1.45695	$C_{12}H_{25}N_3O_3$	260.1961463	242.0630, 242.1125, 242.1407, 242.1859, 243.1345, 243.1701, 260.0314, 260.1270, 260.1599, 260.1966	M+H	POS	Amino Acids, Peptides and derivatives

**M0159**	Inosine	1.495383	$C_{10}H_{12}N_4O_5$	267.0731209	221.0659, 223.0239, 223.1193, 224.0561, 227.4933, 231.0504, 248.9343, 266.9801, 267.0160, 267.0732	M-H	NEG	Nucleotides and derivatives
**M0160**	Guanosine	1.495383	$C_{10}H_{13}N_5O_5$	282.084125	212.2592, 215.9837, 221.0096, 222.0884, 238.0749, 238.1186, 264.9984, 279.9172, 281.8116, 282.0837	M-H, M+FA-H	NEG	Nucleotides and derivatives
**M0161**	N-(1-Deoxy-1-fructosyl)leucine	1.536067	$C_{12}H_{23}NO_7$	292.1400607	210.7080, 214.1073, 228.9460, 230.9601, 232.1177, 244.1165, 246.0983, 274.1294, 292.0852, 292.1378	M-H	NEG	Amino Acids, Peptides and derivatives
**M0162**	4-Acetamidobutanoic acid	1.638683	$C_6H_{11}NO_3$	144.0666115	93.3805, 94.4161, 95.7764, 100.0763, 101.1469, 102.0556, 105.9404, 119.2571, 144.0448, 144.0658	M-H	NEG	Amino Acids, Peptides and derivatives
**M0163**	Isoleucyl-Glutamate	1.635933	$C_{11}H_{20}N_2O_5$	261.1437356	229.0991, 237.9565, 243.1351, 244.0814, 244.1174, 244.1746, 256.1750, 261.0051, 261.0921, 261.1441	M+H	POS	Amino Acids, Peptides and derivatives
**M0164**	4-Pyridoxic acid	1.6589	$C_8H_9NO_4$	182.0458495	138.0558, 139.0405, 143.1609, 152.0346, 154.0990, 164.0347, 165.0188, 182.0088, 182.0193, 182.0456	M-H	NEG	Pyridines and derivatives
**M0165**	Pelargonic acid	10.42392	$C_9H_{18}O_2$	315.2538293	266.9855, 274.9930, 279.2330, 294.9804, 297.2433, 313.2383, 313.2609, 314.9832, 315.1952, 315.2536	$_2$M-H	NEG	Fatty Acyls
**M0166**	Oleamide	12.70553	$C_{18}H_{35}NO$	282.2784129	191.1788, 198.1856, 208.6484, 208.6701, 210.9444, 212.2007, 247.2419, 265.2519, 282.2253, 282.2788	M+H	POS	Fatty Acyls
**M0167**	13-Docosenamide	14.84265	$C_{22}H_{43}NO$	338.3406581	226.2187, 240.2299, 254.2470, 268.2615, 296.2930, 303.3040, 304.3091, 321.3138, 322.3203, 338.3408	M+H	POS	Fatty Acyls
**M0168**	2'-O-Methyladenosine	1.999783	$C_{11}H_{15}N_5O_4$	282.1189795	221.1389, 223.1080, 240.9960, 248.1020, 259.0032, 264.0862, 264.1316, 265.1289, 277.0125, 282.1194	M+H	POS	Nucleotides and derivatives

**M0169**	L-Phenylalanine	2.274467	$C_9H_{11}NO_2$	164.0716837	138.2311, 143.9694, 144.1213, 147.0323, 147.0449, 160.2835, 161.8731, 162.4389, 164.0348, 164.0712	M-H	NEG	Amino Acids, Peptides and derivatives
**M0170**	N-(1-Deoxy-1-fructosyl)phenylalanine	2.3276	$C_{15}H_{21}NO_7$	328.1380416	250.9989, 252.9956, 264.1219, 282.1309, 292.1169, 292.1560, 310.1276, 328.0496, 328.1022, 328.1403	M+H-H_2O, M+H	POS	Amino Acids, Peptides and derivatives
**M0171**	Asparaginylisoleucine	2.428317	$C_{10}H_{19}N_3O_4$	246.1442769	222.5384, 224.2122, 228.1337, 229.1178, 230.1627, 246.0359, 246.0734, 246.1119, 246.1205, 246.1433	M+H	POS	Amino Acids, Peptides and derivatives
**M0172**	Alanylisoleucine	2.489817	$C_9H_{18}N_2O_3$	203.1387373	175.1468, 177.0071, 179.9906, 182.0518, 185.0436, 185.1282, 203.0523, 203.0798, 203.1179, 203.1389	M+H, M+Na	POS	Amino Acids, Peptides and derivatives
**M0173**	Glycylleucine	2.61515	$C_8H_{16}N_2O_3$	189.1231258	143.1177, 144.0806, 149.5007, 158.4376, 161.0600, 171.1129, 189.0539, 189.0746, 189.1029, 189.1242	M+H	POS	Amino Acids, Peptides and derivatives
**M0174**	Ser-Leu	2.61515	$C_9H_{18}N_2O_4$	219.13361	201.0667, 201.1230, 203.1427, 205.1585, 219.0190, 219.0469, 219.0665, 219.0734, 219.1118, 219.1338	M+H	POS	Amino Acids, Peptides and derivatives
**M0175**	Asparaginyl-Leucine	2.675833	$C_{10}H_{19}N_3O_4$	246.1442916	211.1317, 212.0911, 212.1154, 220.4158, 228.1335, 229.1178, 229.1546, 246.0730, 246.1180, 246.1457	M+H	POS	Amino Acids, Peptides and derivatives
**M0176**	Aspartyl-Isoleucine	2.71865	$C_{10}H_{18}N_2O_5$	247.1282549	224.9270, 229.0660, 229.1181, 230.0813, 230.1209, 247.0135, 247.0606, 247.0773, 247.1049, 247.1289	M+H	POS	Amino Acids, Peptides and derivatives
**M0177**	Glutamylisoleucine	2.71865	$C_{11}H_{20}N_2O_5$	261.1438429	218.9063, 225.1231, 237.9094, 243.1332, 244.1390, 260.8589, 261.0363, 261.0421, 261.0916, 261.1295	M+H	POS	Amino Acids, Peptides and derivatives
**M0178**	N-Acetylproline	2.75945	$C_7H_{11}NO_3$	156.0665811	114.0470, 114.0559, 114.0651, 116.3714, 116.5003, 129.4285, 138.0197, 154.7509, 155.8654, 156.0664	M-H	NEG	Amino Acids, Peptides and derivatives

**M0179**	3-(3,4-Dihydroxyphenyl)lactic acid	2.75945	$C_9H_{10}O_5$	243.0509189	198.1227, 200.0560, 209.4190, 210.1954, 225.0388, 227.1042, 229.1694, 240.5631, 242.8721, 243.0506	M-H, M+FA-H	NEG	Organic acids and derivatives
**M0180**	Pantothenic acid	2.782417	$C_9H_{17}NO_5$	218.1033902	208.2087, 208.8684, 209.0394, 209.8896, 209.8947, 215.9840, 217.8530, 217.9841, 218.0475, 218.1031	M-H	NEG	Amino Acids, Peptides and derivatives
**M0181**	Glycyl-Isoleucine	2.945933	$C_8H_{16}N_2O_3$	189.1231122	165.9606, 171.0647, 171.1122, 174.8918, 189.0545, 189.0659, 189.0726, 189.0923, 189.0992, 189.1250	M+H	POS	Amino Acids, Peptides and derivatives
**M0182**	Threonylisoleucine	2.9859	$C_{10}H_{20}N_2O_4$	233.1491525	216.0292, 217.1058, 232.9273, 233.0225, 233.0399, 233.0625, 233.0902, 233.1194, 233.1270, 233.1489	M+H	POS	Amino Acids, Peptides and derivatives
**M0183**	Succinyladenosine	3.086783	$C_{14}H_{17}N_5O_8$	384.1137607	305.2072, 306.9877, 309.0040, 318.9892, 322.5718, 325.0054, 336.9996, 367.0081, 384.0140, 384.1136	M+H	POS	Nucleotides and derivatives
**M0184**	Aspartyl-Leucine	3.108183	$C_{10}H_{18}N_2O_5$	247.1283415	224.9268, 229.1175, 230.0808, 237.0274, 239.6304, 247.0579, 247.0676, 247.0816, 247.1077, 247.1299	M+H	POS	Amino Acids, Peptides and derivatives
**M0185**	Glutamylleucine	3.189183	$C_{11}H_{20}N_2O_5$	261.1438295	237.9084, 237.9836, 238.9728, 243.1333, 257.0329, 261.0022, 261.0359, 261.0836, 261.0889, 261.1291	M+H, M+Na, M+H-H_2O	POS	Amino Acids, Peptides and derivatives
**M0186**	N2,N2-Dimethylguanosine	3.189183	$C_{12}H_{17}N_5O_5$	312.1291612	242.8180, 246.1345, 252.9877, 266.1497, 269.5718, 276.0850, 284.0893, 294.0960, 312.0731, 312.1119	M+H	POS	Nucleotides and derivatives
**M0187**	Protocatechuic acid	3.262267	$C_7H_6O_4$	153.0193809	128.1350, 132.9919, 135.0087, 135.0448, 136.9878, 137.9953, 151.0033, 152.8946, 152.9795, 153.0211	M-H, $_2$M-H	NEG	Organic acids and derivatives
**M0188**	Serylphenylalanine	3.639333	$C_{12}H_{16}N_2O_4$	253.1178985	206.0810, 207.1124, 209.1640, 218.0923, 225.1336, 235.1095, 236.0975, 236.1032, 253.0700, 253.1211	M+H	POS	Amino Acids, Peptides and derivatives

**M0189**	Asparaginyl-Phenylalanine	3.699883	$C_{13}H_{17}N_3O_4$	280.1285774	244.1074, 245.0916, 246.0753, 246.1054, 247.0807, 262.0685, 262.1174, 263.1019, 280.0779, 280.1239	M+H	POS	Amino Acids, Peptides and derivatives
**M0190**	Glycyl-Phenylalanine	3.759667	$C_{11}H_{14}N_2O_3$	223.1074196	205.1187, 206.0813, 206.1544, 209.2554, 211.0725, 220.0765, 222.5479, 223.0231, 223.0564, 223.1097	M+H	POS	Amino Acids, Peptides and derivatives
**M0191**	Isoleucyl-Valine	3.7624	$C_{11}H_{22}N_2O_3$	229.1556517	185.1657, 209.7247, 209.7437, 209.9830, 211.1087, 211.1588, 229.0161, 229.0722, 229.1206, 229.1561	M-H	NEG	Amino Acids, Peptides and derivatives
**M0192**	N-Acetylvaline	3.785	$C_7H_{13}NO_3$	158.0821594	124.6156, 130.9831, 132.3844, 132.4121, 134.4144, 139.5722, 141.4557, 145.2462, 158.0669, 158.0820	M-H	NEG	Amino Acids, Peptides and derivatives
**M0193**	Alanylphenylalanine	3.8386	$C_{12}H_{16}N_2O_3$	237.1229608	203.0815, 219.0642, 219.0827, 219.1127, 220.0962, 220.1074, 237.0164, 237.0397, 237.0706, 237.1247	M+H	POS	Amino Acids, Peptides and derivatives
**M0194**	5'-Methylthioadenosine	3.85855	$C_{11}H_{15}N_5O_3S$	298.0963255	222.0766, 239.1028, 252.0906, 262.1061, 264.0862, 280.0827, 280.1175, 280.1528, 282.1081, 298.0962	M+H	POS	Nucleotides and derivatives
**M0195**	3,4-Dihydroxybenzaldehyde	4.078317	$C_7H_6O_3$	139.0389948	55.0548, 56.9653, 65.0391, 70.0011, 93.0338, 93.0703, 94.0649, 97.0652, 111.0445, 139.0389	M+H	POS	Phenols
**M0196**	Valylphenylalanine	4.30435	$C_{14}H_{20}N_2O_3$	265.1542576	245.0395, 247.0592, 247.1049, 247.1118, 247.1408, 248.0580, 248.0911, 265.0685, 265.1054, 265.1544	M+H	POS	Amino Acids, Peptides and derivatives
**M0197**	Phthalic acid	4.314383	$C_8H_6O_4$	165.0192143	137.0238, 137.0599, 141.1668, 141.8927, 147.0297, 147.0450, 149.0243, 150.0325, 164.8971, 165.0193	M-H	NEG	Organic acids and derivatives
**M0198**	Pimelic acid	4.35425	$C_7H_{12}O_4$	159.0661695	101.0245, 102.9887, 111.0088, 115.0763, 129.0188, 130.9835, 141.0556, 158.8929, 159.0305, 159.0660	M-H, $_2$M-H	NEG	Fatty Acyls

**M0199**	N-Acetylleucine	4.601033	$C_8H_{15}NO_3$	172.0978613	118.2344, 128.0795, 128.1080, 130.0872, 134.4382, 150.4888, 152.9957, 154.9981, 172.0407, 172.0976	M-H	NEG	Amino Acids, Peptides and derivatives
**M0200**	3-Hydroxybenzaldehyde	4.68905	$C_7H_6O_2$	123.04416	96.0450, 96.0526, 99.9257, 100.0240, 105.0448, 105.0704, 108.0572, 111.3781, 112.0394, 123.0441	M+H	POS	Phenols
**M0201**	cis-p-Coumaric acid	4.902817	$C_9H_8O_3$	147.0439155	94.2325, 105.0708, 108.2535, 112.8392, 114.4379, 119.0492, 119.0858, 129.8410, 138.6543, 147.0437	M+H-H_2O, M+H	POS	Organic acids and derivatives
**M0202**	Suberic acid	4.904733	$C_8H_{14}O_4$	173.0817777	146.9045, 152.9967, 153.1740, 154.9468, 154.9987, 155.0711, 172.0422, 172.9525, 173.0090, 173.0816	M-H	NEG	Fatty Acyls
**M0203**	N-Acetyltryptophan	5.024633	$C_{13}H_{14}N_2O_3$	245.0930098	209.2133, 209.2334, 217.0131, 217.0519, 224.9978, 227.0923, 227.1280, 245.0081, 245.0451, 245.0927	M-H	NEG	Amino Acids, Peptides and derivatives
**M0204**	4-formyl Indole	5.460883	C_9H_7NO	146.0598675	109.5397, 113.2847, 118.0652, 118.0756, 119.0492, 119.0858, 126.7408, 135.3059, 140.3132, 146.0597	M+H	POS	Indoles and derivatives
**M0205**	Salicylic acid	5.631933	$C_7H_6O_3$	137.0243536	113.1988, 124.7474, 125.1356, 132.4124, 134.7157, 136.4133, 136.8628, 136.8910, 137.0123, 137.0242	M-H	NEG	Organic acids and derivatives
**M0206**	Traumatic acid	7.221417	$C_{12}H_{20}O_4$	227.1288679	209.1159, 210.5166, 210.5353, 224.8087, 226.9940, 227.0146, 227.0350, 227.0714, 227.1005, 227.1286	M-H	NEG	Fatty Acyls
**M0207**	Dodecanedioic acid	7.6346	$C_{12}H_{22}O_4$	229.1444301	211.1112, 211.1338, 211.1813, 212.4403, 212.7369, 217.9321, 228.9895, 229.0072, 229.0126, 229.1441	M-H	NEG	Fatty Acyls
**M0208**	Phytosphingosine	9.738317	$C_{18}H_{39}NO_3$	318.2994341	297.1926, 300.2534, 300.2889, 301.2135, 305.2557, 318.1730, 318.1801, 318.1984, 318.2125, 318.2994	M+H	POS	Sphingolipids

**M0209**	Dencichine	0.82105	$C_5H_8N_2O_5$	175.0358836		M-H	NEG	Amino Acids, Peptides and derivatives
**M0210**	Galacturonic acid	0.82105	$C_6H_{10}O_7$	387.0778912		$_2$M-H	NEG	Carbohydrates and Glycosides
**M0211**	Manosamine	0.831683	$C_6H_{13}NO_5$	218.0422532		M+K	POS	Carbohydrates and Glycosides
**M0212**	Spectinomycin	0.831683	$C_{14}H_{24}N_2O_7$	333.16444		M+H	POS	Carbohydrates and Glycosides
**M0213**	Allitol	0.869833	$C_6H_{14}O_6$	221.0415747		M+K	POS	Carbohydrates and Glycosides
**M0214**	Azetidine-2-carboxylic acid	0.885033	$C_4H_7NO_2$	146.0457134		M+FA-H	NEG	Amino Acids, Peptides and derivatives
**M0215**	D-altrofurano-heptulose-3	0.904983	$C_7H_{14}O_7$	191.0559503	172.9886, 173.0090, 173.1039, 178.0556, 182.9359, 186.7006, 191.0195, 191.0543, 209.0300, 209.0634	M-H_2O-H, M+FA-H, M-H	NEG	Carbohydrates and Glycosides
**M0216**	Trigonelline	0.905317	$C_7H_7NO_2$	176.0103321		M+K	POS	Alkaloids
**M0217**	3-Pyridineacetic acid	0.924767	$C_7H_7NO_2$	160.0366721		M+Na	POS	Carboxylic acid and derivatives
**M0218**	Stachydrine	0.924767	$C_7H_{13}NO_2$	182.0574328		M+K	POS	Alkaloids
**M0219**	Pinitol	0.92405	$C_7H_{14}O_6$	239.077005	221.0458, 221.0668, 223.0286, 238.8684, 238.8922, 238.9268, 238.9625, 239.0201, 239.0579, 239.0774	M+FA-H	NEG	Carbohydrates and Glycosides
**M0220**	Cyclic N-Acetyl-D-mannosamine	0.924767	$C_8H_{15}NO_6$	260.0523487	200.5463, 201.0064, 209.0495, 216.1154, 219.0159, 235.0656, 236.0674, 237.0278, 242.1027, 260.0524	M+K	POS	Carbohydrates and Glycosides
**M0221**	Lactobionic acid	0.92405	$C_{12}H_{22}O_{12}$	403.1087868	129.0193, 131.0353, 141.0197, 149.0457, 159.0300, 161.0459, 161.5260, 165.0399, 229.4701, 357.1037	M-H, M+FA-H	NEG	Carbohydrates and Glycosides
**M0222**	Methyl beta-D-Galactopyranoside	0.941217	$C_7H_{14}O_6$	193.0713546		M-H	NEG	Carbohydrates and Glycosides
**M0223**	Aceglutamide	0.962317	$C_7H_{12}N_2O_4$	171.076113		M+H-H_2O	POS	Amino Acids, Peptides and derivatives

**M0224**	Fosfomycin	0.957183	$C_3H_7O_4P$	183.0064869		M+FA-H	NEG	Others
**M0225**	delta-Valerobetaine	1.011067	$C_8H_{17}NO_2$	160.1328465		M+H	POS	Others
**M0226**	Erythorbic acid	1.068383	$C_6H_8O_6$	221.0300032		M+FA-H	NEG	Carbohydrates and Glycosides
**M0227**	Guanine	1.0832	$C_5H_5N_5O$	152.0564198	128.9857, 129.0205, 134.0458, 134.0591, 134.0963, 135.0300, 146.9963, 147.0137, 151.5872, 152.0564	M+H	POS	Alkaloids
**M0228**	3-Hydroxy-2-methylpyridine	1.1949	C_6H_7NO	110.0602974	96.0096, 96.5310, 97.0097, 98.5123, 100.0246, 105.0034, 105.5367, 107.4762, 108.0385, 110.0602	M+H	POS	Alkaloids
**M0229**	Cytisinicline	1.251017	$C_{11}H_{14}N_2O$	191.1174541		M+H	POS	Alkaloids
**M0230**	N-Hydroxypipecolic acid	1.3112	$C_6H_{11}NO_3$	144.0664481		M-H	NEG	Carboxylic acid and derivatives
**M0231**	Deacetylasperulosidic Acid	1.351833	$C_{16}H_{22}O_{11}$	389.108543	183.0660, 191.0175, 191.0351, 209.0230, 209.0454, 210.0883, 227.0559, 331.0669, 343.1134, 389.1084	M-H, $_2$M-H	NEG	Terpenes
**M0232**	alpha-Arbutin	1.515783	$C_{12}H_{16}O_7$	317.0876081	262.9602, 271.0823, 274.0354, 282.0839, 299.0422, 299.0726, 299.0837, 316.4123, 317.0508, 317.0954	M+FA-H	NEG	Carbohydrates and Glycosides
**M0233**	3-Hydroxyphenylalanine	1.555033	$C_9H_{11}NO_3$	180.0662528		M-H	NEG	Amino Acids, Peptides and derivatives
**M0234**	Leu-ala	1.635933	$C_9H_{18}N_2O_3$	203.138629	181.0443, 185.0444, 185.0802, 186.0760, 192.5520, 203.0524, 203.0736, 203.0809, 203.1151, 203.1388	M+H	POS	Amino Acids, Peptides and derivatives
**M0235**	4-Hydroxymandelic acid	1.678983	$C_8H_8O_4$	167.03507	123.0559, 124.0154, 125.0248, 138.9287, 139.0394, 141.5750, 152.0110, 166.9998, 167.0200, 167.0343	M-H	NEG	Phenols
**M0236**	Ethylidene-glucose	1.678983	$C_8H_{14}O_6$	205.0716951		M-H	NEG	Carbohydrates and Glycosides
**M0237**	Glycyl-L-tyrosine	1.678983	$C_{11}H_{14}N_2O_4$	219.0774452		M-H_2O-H	NEG	Amino Acids, Peptides and derivatives

**M0238**	Lithospermoside	1.711767	$C_{14}H_{19}NO_8$	347.1439269		M+NH4	POS	Carbohydrates and Glycosides
**M0239**	Gastrodin	1.82345	$C_{13}H_{18}O_7$	331.1030935	271.0122, 271.0457, 271.0822, 285.0659, 285.0974, 303.8450, 305.1517, 313.0557, 331.0663, 331.1010	M+FA-H	NEG	Carbohydrates and Glycosides
**M0240**	Methyl citrate	1.86425	$C_7H_{10}O_7$	205.035187		M-H	NEG	Others
**M0241**	Hydrastinine	1.878767	$C_{11}H_{13}NO_3$	225.1228349		M+NH4	POS	Alkaloids
**M0242**	Methyl (-)-shikimate	1.93765	$C_8H_{12}O_5$	206.1029013		M+NH4	POS	Carboxylic acid and derivatives
**M0243**	2′-O-Methyluridine	1.944283	$C_{10}H_{14}N_2O_6$	257.0776279	214.0721, 225.0393, 228.9588, 232.9024, 239.0598, 256.8474, 256.9138, 256.9897, 257.0279, 257.0701	M-H	NEG	Others
**M0244**	Griffonilide	1.985133	$C_8H_8O_4$	213.0405378		M+FA-H	NEG	Others
**M0245**	3-Galloylquinic acid	1.985133	$C_{14}H_{16}O_{10}$	343.0667057	278.5075, 281.1237, 296.9569, 296.9924, 299.0763, 305.1897, 314.9056, 342.8859, 343.0621, 343.0672	M-H	NEG	Phenols
**M0246**	3'-Methoxyflavonol	10.00652	$C_{16}H_{12}O_4$	269.0802677		M+H	POS	Flavonoids
**M0247**	Sterigmatocystin	10.00998	$C_{18}H_{12}O_6$	305.0453815		M-H2O-H	NEG	Others
**M0248**	Licoflavonol	10.11442	$C_{20}H_{18}O_6$	353.1028701	285.2047, 292.9836, 295.1390, 297.1110, 304.9826, 305.1452, 312.9913, 332.9797, 352.9806, 353.1022	M-H	NEG	Flavonoids
**M0249**	Aristolone	10.23412	$C_{15}H_{22}O$	201.1635395		M+H-H2O	POS	Terpenes
**M0250**	Sesamolin	10.2545	$C_{20}H_{18}O_7$	353.1013951	297.1109, 299.0543, 307.0955, 311.0540, 311.0910, 325.0692, 325.1084, 328.0193, 335.0907, 353.1004	M+H-H2O	POS	Phenylpropanoids
**M0251**	20(R)-Protopanaxatriol	10.2545	$C_{30}H_{52}O_4$	459.3823545		M+H-H2O	POS	Terpenes
**M0252**	Paulownin	10.25807	$C_{20}H_{18}O_7$	351.0871687	323.0907, 330.9830, 330.9905, 330.9980, 333.2400, 336.0626, 336.1023, 350.9836, 350.9926, 351.0865	M-H2O-H	NEG	Phenylpropanoids
**M0253**	Cnidilin	10.25807	$C_{17}H_{16}O_5$	599.1907534		2M-H	NEG	Phenylpropanoids
**M0254**	Quillaic acid	10.27852	$C_{30}H_{46}O_5$	485.3275363	305.1458, 340.9841, 404.0987, 424.9870, 444.9989, 464.9962, 484.9898, 484.9995, 485.2216, 485.3231	M-H	NEG	Terpenes

**M0255**	Gypenoside XIII	10.29898	$C_{41}H_{70}O_{12}$	799.4871932		M+FA-H	NEG	Terpenes
**M0256**	Capsiate	10.31728	$C_{18}H_{26}O_4$	329.1718113		M+Na	POS	Alkaloids
**M0257**	Dihydroguaiaretic acid	10.38258	$C_{20}H_{26}O_4$	329.1761541	249.2204, 264.9890, 268.9825, 285.1525, 285.1853, 288.9897, 305.1991, 308.9962, 328.9933, 329.1748	M-H	NEG	Phenylpropanoids
**M0258**	Atractylenolide II	10.56952	$C_{15}H_{20}O_2$	277.1443957	212.9681, 212.9943, 229.8329, 233.1548, 234.1860, 236.9968, 256.9821, 263.2189, 276.9871, 277.1441	M+FA-H	NEG	Terpenes
**M0259**	Indole-3-methanamine	10.56952	$C_9H_{10}N_2$	291.1602645		$_2$M-H	NEG	Alkaloids
**M0260**	Kuwanon E	10.63405	$C_{25}H_{28}O_6$	423.1815792	362.9889, 382.9845, 382.9936, 396.3571, 402.9845, 402.9899, 410.1391, 422.9915, 422.9981, 423.1787	M-H	NEG	Flavonoids
**M0261**	Sanggenol A	10.6518	$C_{25}H_{28}O_6$	425.1951127		M+H	POS	Flavonoids
**M0262**	Schisantherin C	10.71495	$C_{28}H_{34}O_9$	553.1834496		M+K	POS	Phenylpropanoids
**M0263**	Triphenyl phosphate	10.73567	$C_{18}H_{15}O_4P$	327.0771805		M+H	POS	Others
**M0264**	Lupenone	10.73567	$C_{30}H_{48}O$	407.3662528	269.2260, 271.2420, 283.2418, 297.2575, 339.1890, 349.1819, 351.3033, 365.3184, 407.1870, 407.3658	M+H-H_2O, M+H	POS	Terpenes
**M0265**	Ginsenoside Rk1	10.73567	$C_{42}H_{70}O_{12}$	784.5196778	271.2408, 325.1122, 351.3042, 369.3148, 407.3666, 425.3767, 443.3885, 587.4257, 605.4399, 784.5117	M+H, M+Na, M+NH_4, M+H-H_2O	POS	Terpenes
**M0266**	3-Oxo-4,6-choladien-24-oic acid	10.75613	$C_{24}H_{34}O_3$	371.256958		M+H	POS	Steroids
**M0267**	Alisol A	10.76092	$C_{30}H_{50}O_5$	535.3640827		M+FA-H	NEG	Terpenes
**M0268**	Dahurinol	10.79755	$C_{30}H_{48}O_5$	511.3385209		M+Na	POS	Terpenes
**M0269**	Broussoflavonol F	10.8458	$C_{25}H_{26}O_6$	421.1653307	332.6465, 350.3569, 352.0932, 353.1823, 365.1039, 394.7092, 400.9887, 420.9891, 420.9975, 421.1646	M-H	NEG	Flavonoids
**M0270**	Chrysophanol	10.86648	$C_{15}H_{10}O_4$	253.0505437	212.2706, 212.7080, 212.9964, 215.9837, 225.0548, 232.9824, 238.0266, 245.6346, 252.9892, 253.0504	M-H	NEG	Quinones
**M0271**	Dehydrotumulosic acid	10.92918	$C_{31}H_{48}O_4$	483.3481684		M-H	NEG	Terpenes

**M0272**	Kauniolide	11.00687	$C_{15}H_{18}O_2$	231.1375745	197.9585, 203.1066, 208.3818, 208.4019, 213.1267, 213.8339, 216.0329, 217.0404, 231.0836, 231.1381	M+H	POS	Terpenes
**M0273**	Polyporenic acid C	11.27603	$C_{31}H_{46}O_4$	481.3322966		M-H	NEG	Terpenes
**M0274**	18alpha-Glycyrrhetinic acid	11.46983	$C_{30}H_{46}O_4$	471.3456872	301.4680, 305.2318, 317.2106, 319.0410, 387.7792, 453.2993, 453.3313, 471.1035, 471.1765, 471.3484	M+H	POS	Terpenes
**M0275**	Orthosphenic acid	11.90167	$C_{30}H_{48}O_5$	487.3431143		M-H	NEG	Terpenes
**M0276**	Epibetulinic acid	12.25808	$C_{30}H_{48}O_3$	439.3560199		M+H-H_2O	POS	Terpenes
**M0277**	3-Epiursolic Acid	12.25847	$C_{30}H_{48}O_3$	455.3528609	167.6036, 204.6655, 208.1875, 215.9810, 305.1467, 391.2375, 409.2451, 455.0077, 455.0165, 455.3532	M-H	NEG	Terpenes
**M0278**	Pachymic acid	12.43727	$C_{33}H_{52}O_5$	527.3739419	316.2731, 342.2742, 367.1029, 391.2226, 492.8984, 501.4677, 506.9956, 527.0093, 527.2884, 527.3733	M-H	NEG	Terpenes
**M0279**	Bombykol	12.47075	$C_{16}H_{30}O$	256.2629074	172.3057, 209.2101, 210.7174, 211.5011, 214.0310, 224.8893, 239.1491, 244.0429, 256.1197, 256.2632	M+NH_4	POS	Others
**M0280**	Dibutyl sebacate	12.85307	$C_{18}H_{34}O_4$	337.233868		M+Na	POS	Others
**M0281**	Ganoderol B	13.64923	$C_{30}H_{48}O_2$	485.3636059		M+FA-H	NEG	Terpenes
**M0282**	Paullinic acid	14.50285	$C_{20}H_{38}O_2$	309.2796346		M-H	NEG	Others
**M0283**	N2-Methylguanosine	2.08225	$C_{11}H_{15}N_5O_5$	298.1137386	262.0712, 262.1172, 274.9840, 275.0331, 280.0794, 280.0914, 280.1279, 292.9945, 298.0017, 298.0894	M+H	POS	Others
**M0284**	Triglochinic acid	2.1077	$C_7H_8O_6$	187.0249356		M-H	NEG	Carboxylic acid and derivatives
**M0285**	Melittoside	2.233617	$C_{21}H_{32}O_{15}$	569.1725236		M+FA-H	NEG	Terpenes
**M0286**	Kainic acid	2.387967	$C_{10}H_{15}NO_4$	196.0966144	178.0486, 178.0857, 178.1224, 179.0695, 180.1008, 190.9611, 191.0205, 195.9673, 196.0626, 196.0931	M+H-H_2O	POS	Alkaloids
**M0287**	1-Methyl-6-oxo-1,6-dihydropyridine-3-carboxylic acid	2.53275	$C_7H_7NO_3$	154.04983		M+H	POS	Alkaloids

**M0288**	Phenyl acetate	2.552117	$C_8H_8O_2$	137.0597093	120.7391, 121.1032, 122.0363, 126.9495, 131.9749, 135.2554, 135.7480, 137.0253, 137.0463, 137.0597	M+H	POS	Others
**M0289**	2-Methylcyclopentane-1,3-dione	2.613833	$C_6H_8O_2$	111.0452348		M-H	NEG	Others
**M0290**	3,4,5-Trihydroxybenzaldehyde	2.655617	$C_7H_6O_4$	155.0338007		M+H	POS	Phenols
**M0291**	Norbergenin	2.75945	$C_{13}H_{14}O_9$	359.0625768		M+FA-H	NEG	Phenylpropanoids
**M0292**	Demethylcantharidate	3.046483	$C_8H_{10}O_5$	169.0493598	141.0907, 141.1021, 146.0297, 146.9614, 151.0388, 151.0746, 151.0863, 151.1115, 152.0706, 169.0491	M+H-H_2O	POS	Others
**M0293**	Geniposidic acid	3.200883	$C_{16}H_{22}O_{10}$	373.1137568	229.7428, 233.0457, 236.9720, 304.9169, 304.9601, 305.0688, 343.1009, 355.1047, 372.9539, 373.1151	M-H	NEG	Terpenes
**M0294**	1-Caffeoylquinic acid	3.221383	$C_{16}H_{18}O_9$	353.0876664		M-H	NEG	Phenylpropanoids
**M0295**	8-Aminooctanoic acid	3.536917	$C_8H_{17}NO_2$	160.1329592		M+H	POS	Amino Acids, Peptides and derivatives
**M0296**	Hydroxytyrosol	3.574367	$C_8H_{10}O_3$	153.0556333		M-H	NEG	Phenols
**M0297**	Isovaleramide	3.659467	$C_5H_{11}NO$	102.0917505		M+H	POS	Alkaloids
**M0298**	Caftaric acid	3.678517	$C_{13}H_{12}O_9$	311.0407349		M-H	NEG	Phenylpropanoids
**M0299**	(2RS)-Lotaustralin	3.7624	$C_{11}H_{19}NO_6$	306.1192124		M+FA-H	NEG	Alkaloids
**M0300**	Levoleucovorin	3.759667	$C_{20}H_{23}N_7O_7$	474.1721079		M+H	POS	Alkaloids
**M0301**	8-Epiloganic acid	3.805317	$C_{16}H_{24}O_{10}$	375.129775		M-H	NEG	Terpenes
**M0302**	Picein	3.845733	$C_{14}H_{18}O_7$	343.1032265		M+FA-H	NEG	Carbohydrates and Glycosides
**M0303**	Forsythoside E	3.845733	$C_{20}H_{30}O_{12}$	461.1662103	309.1208, 315.1076, 341.0145, 393.0806, 394.9033, 443.0613, 443.1775, 461.0711, 461.1610, 461.1714	M-H	NEG	Carbohydrates and Glycosides
**M0304**	N-acetyldopamine	3.9067	$C_{10}H_{13}NO_3$	194.0822222		M-H	NEG	Alkaloids
**M0305**	Bergenin	3.949733	$C_{14}H_{16}O_9$	373.0774023		M+FA-H	NEG	Phenylpropanoids
**M0306**	Parishin E	3.949733	$C_{19}H_{24}O_{13}$	459.1140451		M-H	NEG	Alkaloids
**M0307**	Cistanoside F	3.949733	$C_{21}H_{28}O_{13}$	487.1454246	345.1515, 401.1090, 419.1207, 419.1866, 441.0970, 451.1245,	M-H	NEG	Carbohydrates and Glycosides

					469.1284, 478.8114, 487.1395, 487.1516			
**M0308**	Febrifugine	3.978417	$C_{16}H_{19}N_3O_3$	319.1776396		M+NH4	POS	Alkaloids
**M0309**	Daphnin	3.978417	$C_{15}H_{16}O_9$	341.0860634		M+H	POS	Phenylpropanoids
**M0310**	Asperulosidic Acid	4.030417	$C_{18}H_{24}O_{12}$	431.1190842	225.1130, 251.0561, 251.0913, 269.0666, 329.0862, 371.1002, 385.1127, 431.0954, 431.1097, 431.1189	M-H, $_2$M-H	NEG	Terpenes
**M0311**	Skimmin	4.050783	$C_{15}H_{16}O_8$	369.0820594		M+FA-H	NEG	Phenylpropanoids
**M0312**	Jasminoside B	4.171183	$C_{16}H_{26}O_8$	391.1608662	345.1552, 347.1239, 373.0547, 373.1208, 373.1268, 391.0045, 391.0157, 391.0570, 391.1149, 391.1624	M-H, M+FA-H	NEG	Terpenes
**M0313**	2,2-Dimethylsuccinic acid	4.191417	$C_6H_{10}O_4$	191.0560706		M+FA-H	NEG	Carboxylic acid and derivatives
**M0314**	Harmalol	4.191417	$C_{12}H_{12}N_2O$	245.0931969		M+FA-H	NEG	Alkaloids
**M0315**	Sweroside	4.191417	$C_{16}H_{22}O_9$	357.1188069	207.7690, 207.7742, 211.0336, 242.1246, 245.0809, 289.0716, 311.0537, 313.0728, 339.0703, 357.0821	M-H, M+FA-H	NEG	Terpenes
**M0316**	Androsin	4.191417	$C_{15}H_{20}O_8$	373.1138933		M+FA-H	NEG	Carbohydrates and Glycosides
**M0317**	Sibiricose A5	4.191417	$C_{22}H_{30}O_{14}$	517.1559618		M-H	NEG	Carbohydrates and Glycosides
**M0318**	Vicenin-1	4.191417	$C_{26}H_{28}O_{14}$	563.1405251	356.5123, 378.1069, 442.7998, 495.0836, 495.1511, 517.1382, 517.1546, 525.1547, 563.1359, 563.1506	M-H	NEG	Flavonoids
**M0319**	Iriflophenone 3-C-glucoside	4.212933	$C_{19}H_{20}O_{10}$	453.103899		M+FA-H	NEG	Phenols
**M0320**	Regaloside C	4.212933	$C_{18}H_{24}O_{11}$	461.1297509		M+FA-H	NEG	Carbohydrates and Glycosides
**M0321**	Scopolin	4.233283	$C_{16}H_{18}O_9$	399.0932521		M-H$_2$O-H, M+FA-H	NEG	Phenylpropanoids
**M0322**	Afzelechin	4.24175	$C_{15}H_{14}O_5$	257.080506		M+H-H$_2$O	POS	Phenols
**M0323**	5-Feruloylquinic acid	4.25365	$C_{17}H_{20}O_9$	413.1085929		M+FA-H	NEG	Phenylpropanoids
**M0324**	Leucocyanidin	4.261817	$C_{15}H_{14}O_7$	289.0700063		M+H-H$_2$O	POS	Flavonoids
**M0325**	Hydroumbellic acid	4.274067	$C_9H_{10}O_4$	181.0507109		M-H	NEG	Phenylpropanoids

**M0326**	Procyanidin B2	4.314383	$C_{30}H_{26}O_{12}$	577.1360098		M-H	NEG	Flavonoids
**M0327**	Sinapine	4.344133	$C_{16}H_{24}NO_5+$	333.1548012		M+Na	POS	Alkaloids
**M0328**	Fraxin	4.344133	$C_{16}H_{18}O_{10}$	393.0784131	294.1443, 300.1084, 361.1231, 375.0670, 375.1361, 375.1464, 375.2007, 376.1851, 384.2149, 393.0758	M+Na, M+K, M+NH_4	POS	Phenylpropanoids
**M0329**	3-O-Caffeoylquinic acid methyl ester	4.38625	$C_{17}H_{20}O_9$	391.1000768		M+Na	POS	Phenylpropanoids
**M0330**	3',4'-Dihydroxyacetophenone	4.397	$C_8H_8O_3$	151.0399755	123.0450, 124.0166, 130.9930, 131.0352, 133.0508, 136.0162, 149.0455, 150.1919, 150.8557, 151.0399	M-H	NEG	Phenols
**M0331**	Leu-Leu	4.397	$C_{12}H_{24}N_2O_3$	243.1712317	214.7639, 225.0761, 225.1129, 242.9957, 243.0301, 243.0382, 243.0627, 243.0877, 243.1239, 243.1709	M-H	NEG	Amino Acids, Peptides and derivatives
**M0332**	Isomangiferin	4.397	$C_{19}H_{18}O_{11}$	467.0827434		M+FA-H	NEG	Phenylpropanoids
**M0333**	5-O-Caffeoylshikimic acid	4.4266	$C_{16}H_{16}O_8$	319.0807023		M+H-H_2O	POS	Phenylpropanoids
**M0334**	Esculetin	4.437767	$C_9H_6O_4$	177.0192745	132.9721, 133.0293, 133.0410, 133.0656, 134.0613, 135.0083, 149.0241, 162.0320, 176.8424, 177.0191	M-H	NEG	Phenylpropanoids
**M0335**	Regaloside A	4.437767	$C_{18}H_{24}O_{10}$	445.1349204		M+FA-H	NEG	Carbohydrates and Glycosides
**M0336**	Pinoresinol Diglucoside	4.437767	$C_{32}H_{42}O_{16}$	727.2458083		M+FA-H	NEG	Phenylpropanoids
**M0337**	Megastigm-7-ene-3,5,6,9-tetraol	4.45805	$C_{13}H_{24}O_4$	289.1656055		M+FA-H	NEG	Terpenes
**M0338**	2''-O-beta-L-galactopyranosylorientin	4.45805	$C_{27}H_{30}O_{16}$	591.1358204		M-H_2O-H	NEG	Flavonoids
**M0339**	Anisomycin	4.48885	$C_{14}H_{19}NO_4$	266.1382659	222.1488, 223.1199, 224.1273, 230.1171, 231.1364, 248.0951, 248.1279, 249.0767, 249.1504, 266.1378	M+H	POS	Others
**M0340**	Lycobetaine	4.48885	$C_{16}H_{12}NO_3+$	289.0701622		M+Na	POS	Alkaloids
**M0341**	Gaultherin	4.498667	$C_{19}H_{26}O_{12}$	491.1402276	332.1445, 337.5875, 351.1282, 411.3707, 413.1056, 421.3071, 445.1272, 445.1865, 472.6514, 491.1250	M+FA-H	NEG	Carbohydrates and Glycosides

**M0342**	Cynarin	4.498667	$C_{25}H_{24}O_{12}$	515.1198188		M-H	NEG	Phenylpropanoids
**M0343**	Isovanillic acid	4.5404	$C_8H_8O_4$	335.0772239		$_2$M-H	NEG	Phenols
**M0344**	Gentiopicroside	4.5404	$C_{16}H_{20}O_9$	401.1087076		M+FA-H	NEG	Terpenes
**M0345**	Flavanomarein	4.5404	$C_{21}H_{22}O_{11}$	449.1092535		M-H	NEG	Flavonoids
**M0346**	Prunasin	4.560383	$C_{14}H_{17}NO_6$	340.1034705		M+FA-H	NEG	Alkaloids
**M0347**	Isocoreopsin	4.560383	$C_{21}H_{22}O_{10}$	433.1135923		M-H	NEG	Flavonoids
**M0348**	5-Hydroxyferulic acid	4.57055	$C_{10}H_{10}O_5$	193.049344		M+H-H_2O	POS	Phenylpropanoids
**M0349**	2,5-Dihydroxybenzaldehyde	4.580733	$C_7H_6O_3$	275.0559738		$_2$M-H	NEG	Others
**M0350**	4-Feruloylquinic acid	4.580733	$C_{17}H_{20}O_9$	413.1084001		M+FA-H	NEG	Phenylpropanoids
**M0351**	Manghaslin	4.580733	$C_{33}H_{40}O_{20}$	755.2044825	539.7066, 593.1578, 608.1400, 609.1451, 641.2109, 709.0803, 709.1979, 754.3134, 754.3456, 755.2040	M-H	NEG	Flavonoids
**M0352**	Eleutheroside E	4.580733	$C_{34}H_{46}O_{18}$	787.2684806		M+FA-H	NEG	Phenylpropanoids
**M0353**	Quercetagitrin	4.590733	$C_{21}H_{20}O_{13}$	481.0976305		M+H	POS	Flavonoids
**M0354**	Orientin	4.621433	$C_{21}H_{20}O_{11}$	429.0823936		M-H_2O-H	NEG	Flavonoids
**M0355**	Luteolin 5-O-glucoside	4.629633	$C_{21}H_{20}O_{11}$	431.0962718		M+H-H_2O	POS	Flavonoids
**M0356**	Miquelianin	4.64985	$C_{21}H_{18}O_{13}$	479.0811601		M+H	POS	Flavonoids
**M0357**	Chrysin 6-C-arabinoside 8-C-glucoside	4.6621	$C_{26}H_{28}O_{13}$	547.1449805		M-H	NEG	Flavonoids
**M0358**	Shancigusin I	4.6621	$C_{28}H_{34}O_{14}$	639.1928589		M+FA-H	NEG	Phenylpropanoids
**M0359**	Leucic acid	4.6818	$C_6H_{12}O_3$	131.0712382		M-H	NEG	Carboxylic acid and derivatives
**M0360**	11-O-Galloylbergenin	4.6818	$C_{21}H_{20}O_{13}$	479.0829503	317.0563, 327.1087, 357.1179, 431.1304, 431.1365, 433.2415, 435.1299, 449.1451, 461.0696, 479.0783	M-H	NEG	Phenylpropanoids
**M0361**	Rutarin	4.6818	$C_{20}H_{24}O_{10}$	847.2677325		$_2$M-H	NEG	Phenylpropanoids
**M0362**	Taxifolin 3'-O-glucoside	4.701583	$C_{21}H_{22}O_{12}$	465.1040216		M-H	NEG	Flavonoids
**M0363**	Apigenin-6-C-beta-D-xylopyranosyl-8-C-alpha-L-arabinopyranoside	4.701583	$C_{25}H_{26}O_{13}$	533.1296855		M-H	NEG	Flavonoids
**M0364**	Lyoniresinol 9'-O-glucoside	4.701583	$C_{28}H_{38}O_{13}$	627.2292469	386.1381, 389.1656, 404.1456, 419.1719, 450.1114, 451.1140, 491.0994, 581.2237, 584.1005, 627.1455	M-H, M+FA-H	NEG	Phenylpropanoids

**M0365**	Tropine	4.727383	$C_8H_{15}NO$	142.1225836		M+H	POS	Alkaloids
**M0366**	Clovamide	4.7616	$C_{18}H_{17}NO_7$	717.1950449		$_2$M-H	NEG	Phenylpropanoids
**M0367**	Picrocrocin	4.766817	$C_{16}H_{26}O_7$	353.1563233		M+Na	POS	Terpenes
**M0368**	Chrysin 6-C-glucoside 8-C-arabinoside	4.8229	$C_{26}H_{28}O_{13}$	547.1455116		M-H	NEG	Flavonoids
**M0369**	Liquiritin apioside	4.8229	$C_{26}H_{30}O_{13}$	549.1610884	399.1088, 406.4602, 417.1172, 429.1107, 503.2502, 517.1393, 543.2939, 543.3060, 545.9246, 549.1610	M-H	NEG	Flavonoids
**M0370**	6"-O-Apiosyl-5-O-Methylvisammioside	4.8229	$C_{27}H_{36}O_{14}$	565.1926055		M-H_2O-H	NEG	Carbohydrates and Glycosides
**M0371**	Jionoside B1	4.8229	$C_{37}H_{50}O_{20}$	813.2830586		M-H	NEG	Carbohydrates and Glycosides
**M0372**	Protosappanin A	4.825417	$C_{15}H_{12}O_5$	273.0750602	208.2618, 223.0450, 225.0542, 230.0787, 231.0642, 237.1956, 245.0981, 252.0945, 255.0659, 273.0749	M+H	POS	Phenols
**M0373**	6-Hydroxycoumarin	4.864317	$C_9H_6O_3$	161.0243828		M-H	NEG	Phenylpropanoids
**M0374**	Cynaroside	4.86395	$C_{21}H_{20}O_{11}$	431.0962256		M+H-H_2O	POS	Flavonoids
**M0375**	Coniferyl alcohol	4.883433	$C_{10}H_{12}O_3$	198.112247		M+NH_4	POS	Alkaloids
**M0376**	Isookanin	4.884717	$C_{15}H_{12}O_6$	287.0552463		M-H	NEG	Flavonoids
**M0377**	Isoquercetin	4.883433	$C_{21}H_{20}O_{12}$	465.1024351	335.0929, 352.0781, 355.1720, 441.2292, 452.1698, 469.2174, 486.2785, 486.2880, 486.5795, 487.0844	M+H, M+Na	POS	Phenols
**M0378**	Epiafzelechin	4.902817	$C_{15}H_{14}O_5$	257.0802705	229.0864, 239.0697, 239.1264, 239.1629, 239.2113, 242.0315, 242.0569, 256.0499, 256.8209, 257.0799	M+H-H_2O, M+NH_4	POS	Phenols
**M0379**	Isolindleyin	4.9237	$C_{23}H_{26}O_{11}$	477.1400107		M-H	NEG	Carbohydrates and Glycosides
**M0380**	4-Hydroxyacetophenone	4.944233	$C_8H_8O_2$	135.0451008	106.8650, 108.0214, 109.9219, 124.0158, 126.1284, 133.0558, 134.8652, 134.8829, 134.8945, 135.0450	M-H	NEG	Others
**M0381**	m-Anisaldehyde	4.942183	$C_8H_8O_2$	137.059627	122.0360, 122.0718, 124.3732, 126.6536, 127.8911, 133.6779, 133.8390, 137.0233, 137.0467, 137.0594	M+H	POS	Carboxylic acid and derivatives

**M0382**	Armillarisin A	4.944233	$C_{12}H_{10}O_5$	279.0507505	235.1340, 240.0388, 244.0603, 245.9858, 261.0018, 262.0713, 278.1039, 278.9835, 279.0151, 279.0508	M-H, M+FA-H	NEG	Phenylpropanoids
**M0383**	Ellagic acid	4.944233	$C_{14}H_6O_8$	300.9987701		M-H	NEG	Phenols
**M0384**	Robinetin	4.944233	$C_{15}H_{10}O_7$	301.0346898		M-H	NEG	Flavonoids
**M0385**	3,4-Dicaffeoylquinic acid	4.944233	$C_{25}H_{24}O_{12}$	561.1248016		M+FA-H	NEG	Phenylpropanoids
**M0386**	4-Hydroxybenzyl cyanide	4.960817	C_8H_7NO	134.0600112		M+H	POS	Alkaloids
**M0387**	Eriodictyol	4.960817	$C_{15}H_{12}O_6$	271.0593667	241.0489, 242.0564, 243.0661, 248.0429, 250.5443, 253.0493, 255.0646, 259.5523, 270.2789, 271.0598	M+H-H_2O	POS	Flavonoids
**M0388**	Agarotetrol	4.960817	$C_{17}H_{18}O_6$	319.1169546	273.1180, 283.0601, 301.0349, 301.0696, 301.1112, 301.1485, 302.1160, 302.1538, 319.0424, 319.0793	M+H	POS	Others
**M0389**	7-[(beta-D-Glucopyranosyl)oxy]-3',4',5,8-tetrahydroxyflavone	4.963617	$C_{21}H_{20}O_{12}$	463.0880882	287.0552, 300.0272, 301.0347, 301.0710, 317.0512, 331.1762, 375.1295, 417.1160, 445.0737, 463.0879	M-H	NEG	Flavonoids
**M0390**	Isoacteoside	4.963617	$C_{29}H_{36}O_{15}$	623.1981843	441.1681, 443.1745, 447.0908, 447.1007, 461.0735, 461.1669, 463.0869, 577.2892, 605.1172, 623.1993	M-H	NEG	Carbohydrates and Glycosides
**M0391**	Indole-3-carbinol	4.98065	C_9H_9NO	148.0755869		M+H	POS	Alkaloids
**M0392**	Laricitrin 3-O-glucoside	4.984017	$C_{22}H_{22}O_{13}$	493.0996864	330.0370, 331.0470, 349.0599, 371.0620, 431.0983, 433.0845, 457.1696, 492.1906, 492.1988, 493.0992	M-H	NEG	Flavonoids
**M0393**	Galloylpaeoniflorin	4.98065	$C_{30}H_{32}O_{15}$	650.2067944	319.1168, 331.0805, 333.0796, 349.0914, 374.2092, 475.1236, 493.1342, 511.1394, 649.7921, 650.2259	M+NH_4, M+Na	POS	Terpenes
**M0394**	5-Acetylsalicylic acid	5.00415	$C_9H_8O_4$	179.0349469		M-H	NEG	Phenols
**M0395**	Swertiaside	5.0009	$C_{23}H_{28}O_{12}$	479.1530553		M+H-H_2O	POS	Terpenes
**M0396**	Quercetin 3-O-malonylglucoside	5.00415	$C_{24}H_{22}O_{15}$	531.0759295		M-H_2O-H	NEG	Flavonoids

**M0397**	Isorhoifolin	5.00415	$C_{27}H_{30}O_{14}$	577.1551648		M-H	NEG	Flavonoids
**M0398**	Camellianin A	5.00415	$C_{29}H_{32}O_{15}$	619.1680876		M-H	NEG	Flavonoids
**M0399**	3-Hydroxy-4-methoxyacetophenone	5.024633	$C_9H_{10}O_3$	147.0451115		M-H_2O-H	NEG	Phenols
**M0400**	Osmundacetone	5.024633	$C_{10}H_{10}O_3$	177.0556348	173.8523, 175.9788, 176.0467, 176.2850, 176.4613, 176.8163, 176.8368, 176.8429, 177.0191, 177.0555	M-H	NEG	Phenylpropanoids
**M0401**	Nodakenin	5.024633	$C_{20}H_{24}O_9$	389.1241022	181.0509, 193.0505, 195.0657, 195.0827, 227.0712, 341.1042, 343.1157, 345.0605, 389.0776, 389.1335	M-H_2O-H	NEG	Phenylpropanoids
**M0402**	Tosufloxacin	5.024633	$C_{19}H_{15}F_3N_4O_3$	449.108607		M+FA-H	NEG	Others
**M0403**	Sophorabioside	5.024633	$C_{27}H_{30}O_{14}$	623.1618921		M+FA-H	NEG	Flavonoids
**M0404**	Narcissin	5.020233	$C_{28}H_{32}O_{16}$	625.1755083		M+H	POS	Flavonoids
**M0405**	Curculigoside B	5.0456	$C_{21}H_{24}O_{11}$	451.123951		M-H	NEG	Carbohydrates and Glycosides
**M0406**	Sinapinic acid	5.065517	$C_{11}H_{12}O_5$	223.0611717	207.9668, 208.0105, 208.0374, 209.4979, 211.0450, 221.5426, 222.8448, 223.0226, 223.0329, 223.0611	M-H	NEG	Phenylpropanoids
**M0407**	Cimifugin	5.07945	$C_{16}H_{18}O_6$	307.1168063	266.0396, 274.0827, 284.1802, 284.6819, 289.1070, 289.1422, 289.1773, 292.0927, 305.7750, 307.1165	M+H, M+Na	POS	Others
**M0408**	Pinoresinol 4-O-beta-D-glucopyranoside	5.07945	$C_{26}H_{32}O_{11}$	543.1823059		M+NH_4, M+Na	POS	Phenylpropanoids
**M0409**	Fusaric acid	5.09965	$C_{10}H_{13}NO_2$	180.1017013	162.0627, 162.0910, 163.0390, 163.0748, 163.1107, 164.0705, 165.0914, 180.0110, 180.0669, 180.1012	M+H-H_2O, M+H	POS	Alkaloids
**M0410**	Antiarol	5.09965	$C_9H_{12}O_4$	185.0806688		M+H	POS	Others
**M0411**	Umbelliferone	5.105167	$C_9H_6O_3$	161.0244209		M-H	NEG	Phenylpropanoids
**M0412**	Sinapyl alcohol	5.105167	$C_{11}H_{14}O_4$	191.0713703		M-H_2O-H	NEG	
**M0413**	Avicularin	5.105167	$C_{20}H_{18}O_{11}$	433.0776491		M-H_2O-H, M-H	NEG	Flavonoids
**M0414**	3'-Methoxyapiin	5.105167	$C_{27}H_{30}O_{15}$	575.1413421		M-H_2O-H	NEG	Flavonoids
**M0415**	Diosmin	5.105167	$C_{28}H_{32}O_{15}$	653.1733038		M+FA-H	NEG	Flavonoids
**M0416**	Guaifenesin	5.120067	$C_{10}H_{14}O_4$	199.0962719		M+H	POS	Others

**M0417**	Quercitrin	5.120067	$C_{21}H_{20}O_{11}$	471.0890221		M+Na	POS	Flavonoids
**M0418**	2,6-Dimethoxybenzoic acid	5.125517	$C_9H_{10}O_4$	181.0506211		M-H	NEG	Carboxylic acid and derivatives
**M0419**	Scopoletin	5.125517	$C_{10}H_8O_4$	191.0349256		M-H	NEG	Phenylpropanoids
**M0420**	Picrotin	5.160017	$C_{15}H_{18}O_7$	293.1011928	247.0962, 252.0732, 261.0341, 261.0709, 275.0905, 275.1284, 275.1618, 275.1992, 289.0699, 293.1002	M+H-H_2O, M+Na, M+NH_4	POS	Terpenes
**M0421**	Hesperidin	5.160017	$C_{28}H_{34}O_{15}$	611.1948336		M+H	POS	Flavonoids
**M0422**	Apocynin	5.179483	$C_9H_{10}O_3$	167.0700792	139.0754, 139.1112, 143.9967, 149.0232, 149.0598, 149.0959, 149.1321, 167.0132, 167.0341, 167.0700	M+H, M+Na, M+H-H_2O	POS	Phenols
**M0423**	Peucedanol	5.179483	$C_{14}H_{16}O_5$	247.095617		M+H-H_2O	POS	Phenylpropanoids
**M0424**	Brassicin	5.179483	$C_{22}H_{22}O_{12}$	501.0992884		M+Na	POS	Flavonoids
**M0425**	(2R)-2-[1-[[6-O-(2-Carboxyacetyl)-beta-D-glucopyranosyl]oxy]-1-methylethyl]-2,3-dihydro-7H-furo[3,2-G][1]benzopyran-7-one	5.186017	$C_{23}H_{26}O_{12}$	475.1242727		M-H_2O-H	NEG	Carbohydrates and Glycosides
**M0426**	Fraxidin	5.201533	$C_{11}H_{10}O_5$	245.0414685		M+Na	POS	Phenylpropanoids
**M0427**	Rhodojaponin V	5.201533	$C_{22}H_{34}O_7$	393.2256091		M+H-H_2O	POS	Terpenes
**M0428**	Prunin	5.201533	$C_{21}H_{22}O_{10}$	435.1280184		M+H	POS	Flavonoids
**M0429**	Astragalin	5.208017	$C_{21}H_{20}O_{11}$	447.0934262	299.0178, 300.0268, 301.0359, 314.0426, 327.0495, 367.0463, 401.1816, 401.2220, 446.2093, 447.0925	M-H	NEG	Flavonoids
**M0430**	Isolariciresinol	5.221633	$C_{20}H_{24}O_6$	361.1630255		M+H	POS	Phenylpropanoids
**M0431**	Notoginsenoside R1	5.228367	$C_{47}H_{80}O_{18}$	977.5337307		M+FA-H	NEG	Terpenes
**M0432**	6-Hydroxyluteolin	5.2486	$C_{15}H_{10}O_7$	301.0350667	257.0439, 257.0815, 259.0589, 273.0417, 283.0226, 283.0593, 286.0474, 300.1510, 300.9981, 301.0338	M-H	NEG	Flavonoids
**M0433**	Viscidulin I	5.260967	$C_{15}H_{10}O_7$	303.0490553	257.0792, 261.0757, 271.0966, 275.0536, 285.0388, 285.0747, 285.1152, 285.1844, 288.0537, 303.0494	M+H	POS	Flavonoids
**M0434**	Phlorizin	5.260967	$C_{21}H_{24}O_{10}$	459.1274387		M+Na	POS	Flavonoids

**M0435**	Piperonylic acid	5.2885	$C_8H_6O_4$	211.0247149		M+FA-H	NEG	Carboxylic acid and derivatives
**M0436**	scutellarin Methylester	5.300933	$C_{22}H_{20}O_{12}$	499.0835859		M+Na	POS	Flavonoids
**M0437**	Cinnamamide	5.320433	C_9H_9NO	148.075515		M+H	POS	Phenylpropanoids
**M0438**	Epitheaflagallin 3-O-gallate	5.329283	$C_{27}H_{20}O_{13}$	597.0912557		M+FA-H	NEG	Flavonoids
**M0439**	4,5-Di-O-caffeoylquinic acid methyl ester	5.349733	$C_{26}H_{26}O_{12}$	529.1351093		M-H	NEG	Phenylpropanoids
**M0440**	Aurantio-obtusin beta-D-glucoside	5.370933	$C_{23}H_{24}O_{12}$	473.1086449		M-H_2O-H	NEG	Phenylpropanoids
**M0441**	Viniferin	5.370933	$C_{28}H_{22}O_6$	499.1391805		M+FA-H	NEG	Phenylpropanoids
**M0442**	Chrysin 7-O-beta-gentiobioside	5.370933	$C_{27}H_{30}O_{14}$	577.1553727		M-H, M+FA-H	NEG	Flavonoids
**M0443**	Neohesperidin	5.370933	$C_{28}H_{34}O_{15}$	591.1717744		M-H_2O-H	NEG	Flavonoids
**M0444**	5,7-Dihydroxychromone	5.3905	$C_9H_6O_4$	177.01927		M-H	NEG	Flavonoids
**M0445**	6,7,4'-Trihydroxyisoflavone	5.3905	$C_{15}H_{10}O_5$	269.04529	240.0430, 240.0590, 241.0510, 248.9997, 251.0349, 267.0311, 268.0370, 268.0675, 268.7909, 269.0453	M-H	NEG	Flavonoids
**M0446**	Butin	5.3905	$C_{15}H_{12}O_5$	271.060824		M-H	NEG	Flavonoids
**M0447**	Naringin Dihydrochalcone	5.3905	$C_{27}H_{34}O_{14}$	581.1877376		M-H	NEG	Flavonoids
**M0448**	Rutaretin	5.410867	$C_{14}H_{14}O_5$	243.066092		M-H_2O-H	NEG	Phenylpropanoids
**M0449**	Ginsenoside Re	5.410867	$C_{48}H_{82}O_{18}$	991.5493215	799.4895, 886.4816, 892.6535, 926.8350, 936.0342, 945.1898, 945.5438, 952.9395, 955.1905, 991.5518	M-H, M+FA-H	NEG	Terpenes
**M0450**	Kushenol O	5.420683	$C_{27}H_{30}O_{13}$	563.1743664	264.7698, 269.0802, 285.2827, 291.1720, 466.1953, 480.1862, 480.6822, 489.1960, 562.7323, 563.1700	M+H	POS	Flavonoids
**M0451**	Esculentoside H	5.430967	$C_{48}H_{76}O_{21}$	969.472761		M-H_2O-H	NEG	Terpenes
**M0452**	(19R)-13-Deoxy-19-hydroxyenmein	5.451283	$C_{20}H_{26}O_6$	361.1654819		M-H	NEG	Others
**M0453**	Swertianolin	5.451283	$C_{20}H_{20}O_{11}$	435.0932356		M-H	NEG	Phenylpropanoids
**M0454**	Picroside I	5.460883	$C_{24}H_{28}O_{11}$	515.1529132		M+Na	POS	Terpenes
**M0455**	Tinnevellin glucoside	5.470983	$C_{20}H_{24}O_9$	389.1238007		M-H_2O-H	NEG	Quinones
**M0456**	Silychristin	5.530817	$C_{25}H_{22}O_{10}$	527.1187782		M+FA-H	NEG	Flavonoids

**M0457**	Oroxin A	5.530817	$C_{21}H_{20}O_{10}$	863.2038527		$_2$M-H	NEG	Flavonoids
**M0458**	Secoisolariciresinol	5.551367	$C_{20}H_{26}O_6$	407.1711931		M+FA-H	NEG	Phenylpropanoids
**M0459**	Sinapaldehyde	5.560217	$C_{11}H_{12}O_4$	209.0805469	173.0963, 173.1323, 177.0543, 181.0858, 191.0704, 191.1049, 191.1423, 194.0206, 209.0443, 209.0796	M+H	POS	Phenylpropanoids
**M0460**	Coniferaldehyde	5.571717	$C_{10}H_{10}O_3$	177.0556109		M-H	NEG	Phenylpropanoids
**M0461**	5-Hydroxy-1-tetralone	5.580267	$C_{10}H_{10}O_2$	163.0751146	135.0803, 135.1171, 137.0345, 139.5387, 145.0646, 145.0999, 155.0600, 163.0389, 163.0514, 163.0748	M+H-H_2O, M+H	POS	Phenols
**M0462**	Niga-ichigoside F1	5.598967	$C_{36}H_{58}O_{11}$	689.3861371	122.1555, 151.0385, 185.0426, 208.0676, 208.0903, 347.0898, 472.8977, 527.3334, 527.4082, 689.3878	M+NH_4, M+Na	POS	Terpenes
**M0463**	Resveratrol	5.631933	$C_{14}H_{12}O_3$	273.0767116		M+FA-H	NEG	Phenylpropanoids
**M0464**	Amarogentin	5.631933	$C_{29}H_{30}O_{13}$	567.1510351		M-H_2O-H	NEG	Terpenes
**M0465**	Mudanpiosidec	5.631933	$C_{30}H_{32}O_{13}$	599.1768429		M-H	NEG	Terpenes
**M0466**	Herbacetin	5.672733	$C_{15}H_{10}O_7$	283.024756		M-H_2O-H	NEG	Flavonoids
**M0467**	Methyl caffeic acid	5.69305	$C_{10}H_{10}O_4$	193.0505116		M-H	NEG	Phenylpropanoids
**M0468**	Sulfuretin	5.69305	$C_{15}H_{10}O_5$	269.0448615	267.0313, 267.9286, 268.0377, 268.0681, 268.1548, 268.5929, 268.6034, 268.7911, 268.9909, 269.0451	M-H	NEG	Flavonoids
**M0469**	4-Ethylresorcinol	5.713133	$C_8H_{10}O_2$	183.066202		M+FA-H	NEG	Phenols
**M0470**	Scutellarein	5.713133	$C_{15}H_{10}O_6$	285.040255	241.1445, 243.1598, 253.0512, 257.0453, 267.0290, 267.1216, 270.0529, 282.8474, 284.0327, 285.0410	M-H	NEG	Flavonoids
**M0471**	Sappanone A	5.720333	$C_{16}H_{12}O_5$	285.0750596		M+H, M+Na, M+H-H_2O	POS	Flavonoids
**M0472**	Dihydrobaicalin	5.753383	$C_{21}H_{20}O_{11}$	447.0937924		M-H	NEG	Flavonoids
**M0473**	Benzoyloxypaeoniflorin	5.753383	$C_{30}H_{32}O_{13}$	599.1784299		M-H	NEG	Terpenes
**M0474**	Lariciresinol	5.760633	$C_{20}H_{24}O_6$	383.1460288		M+Na	POS	Phenylpropanoids
**M0475**	Rhodojaponin II	5.77375	$C_{22}H_{34}O_7$	409.2230632		M-H	NEG	Terpenes

**M0476**	2-Butenoic acid, 2-methyl-, (3aS,4S,5S,6E,10Z,11aR)-6-formyl-2,3,3a,4,5,8,9,11a-octahydro-5-hydroxy-10-(hydroxymethyl)-3-methylene-2-oxocyclodeca[b]furan-4-yl ester, (2E)-	5.7832	$C_{20}H_{24}O_7$	399.1415483		M+Na	POS	Others
**M0477**	Isomartynoside	5.794	$C_{31}H_{40}O_{15}$	651.2303525	492.5898, 505.1701, 515.2860, 562.7590, 583.2170, 607.1373, 607.1489, 646.9003, 650.3934, 651.2269	M-H	NEG	Carbohydrates and Glycosides
**M0478**	3,4-Dimethoxycinnamic acid	5.802717	$C_{11}H_{12}O_4$	191.0701058	167.9627, 168.0677, 173.0594, 173.0956, 173.1325, 176.0473, 177.0721, 190.9798, 191.0385, 191.0698	M+H-H_2O	POS	Phenylpropanoids
**M0479**	A-D-Glucopyranoside	5.802717	$C_{21}H_{36}O_{10}$	471.2192663	227.1736, 246.0488, 295.0569, 295.0763, 335.0938, 394.0007, 442.9922, 453.3392, 471.0946, 471.2158	M+NH_4, M+Na	POS	Carbohydrates and Glycosides
**M0480**	Linarin	5.814383	$C_{28}H_{32}O_{14}$	637.1782766		M+FA-H	NEG	Flavonoids
**M0481**	Royal Jelly acid	5.8347	$C_{10}H_{18}O_3$	185.1183904		M-H	NEG	Carboxylic acid and derivatives
**M0482**	Licochalcone B	5.8769	$C_{16}H_{14}O_5$	285.076604		M-H	NEG	Flavonoids
**M0483**	Phaeocaulisin E	5.8769	$C_{15}H_{24}O_3$	297.1705684		M+FA-H	NEG	Terpenes
**M0484**	Tricin	5.883717	$C_{17}H_{14}O_7$	331.0804158		M+H	POS	Flavonoids
**M0485**	Leocarpinolide F	5.8769	$C_{20}H_{24}O_7$	357.1343788		M-H_2O-H	NEG	Terpenes
**M0486**	Daidzein	5.924167	$C_{15}H_{10}O_4$	255.0647045	219.2113, 225.0547, 227.0705, 235.1000, 237.0549, 237.1099, 237.1469, 237.1843, 254.0533, 255.0646	M+H	POS	Flavonoids
**M0487**	Noreugenin	5.938317	$C_{10}H_8O_4$	191.0350421	154.2076, 154.9985, 163.0403, 163.9517, 170.9876, 171.0027, 173.0090, 176.0112, 176.6774, 191.0353	M-H	NEG	Phenols
**M0488**	Aleuritic acid	5.961133	$C_{16}H_{32}O_5$	303.2175801		M-H	NEG	Carboxylic acid and derivatives

**M0489**	Oridonin	6.001833	$C_{20}H_{28}O_6$	345.1700745		M-H_2O-H	NEG	Terpenes
**M0490**	Rhapontigenin	6.0277	$C_{15}H_{14}O_4$	281.0776605		M+Na	POS	Phenylpropanoids
**M0491**	Steppogenin	6.042667	$C_{15}H_{12}O_6$	287.0558736		M-H	NEG	Flavonoids
**M0492**	Dihydrotamarixetin	6.042667	$C_{16}H_{14}O_7$	317.066353	248.9045, 248.9600, 256.0371, 258.0534, 273.0742, 284.0322, 299.0569, 302.0428, 316.6315, 317.0666	M-H	NEG	Flavonoids
**M0493**	Betulinaldehyde	6.047933	$C_{30}H_{48}O_2$	441.3717178		M+H	POS	Terpenes
**M0494**	N-trans-sinapoyltyramine	6.063067	$C_{19}H_{21}NO_5$	342.1343291		M-H	NEG	Phenylpropanoids
**M0495**	(+)-Nortrachelogenin	6.063067	$C_{20}H_{22}O_7$	373.1290836		M-H	NEG	Phenylpropanoids
**M0496**	Desoxyrhaponticin	6.083417	$C_{21}H_{24}O_8$	449.1448552		M+FA-H	NEG	Phenylpropanoids
**M0497**	Linaroside	6.103717	$C_{23}H_{24}O_{11}$	475.1230556		M-H	NEG	Flavonoids
**M0498**	7,8-Dimethoxycoumarin	6.109567	$C_{11}H_{10}O_4$	189.0544169		M+H-H_2O	POS	Phenylpropanoids
**M0499**	Quercetin 5,3'-dimethyl ether	6.109567	$C_{17}H_{14}O_7$	331.0804368		M+H-H_2O, M+H	POS	Flavonoids
**M0500**	Torachrysone-8-O-beta-D-glucoside	6.109567	$C_{20}H_{24}O_9$	431.1303441	320.9226, 322.5393, 337.4943, 368.4220, 388.1132, 389.1191, 413.1207, 414.8296, 416.1107, 431.1323	M+H, M+K, M+Na, M+H-H_2O	POS	Quinones
**M0501**	Nodakenetin	6.1297	$C_{14}H_{14}O_4$	247.0960538	208.5387, 208.5591, 210.0508, 211.1126, 214.0619, 215.0675, 219.1384, 229.0853, 229.1204, 247.0960	M+H-H_2O, M+H	POS	Phenylpropanoids
**M0502**	2'-Aminoacetophenone	6.145033	C_8H_9NO	180.0664421		M+FA-H	NEG	Alkaloids
**M0503**	Morin	6.165517	$C_{15}H_{10}O_7$	301.0350969	273.0400, 280.9841, 283.0258, 283.1911, 285.0409, 286.0479, 299.0195, 300.1822, 300.9981, 301.0349	M-H	NEG	Flavonoids
**M0504**	Quercetin	6.170067	$C_{15}H_{10}O_7$	303.0491723	275.0541, 275.1349, 285.0389, 285.0739, 285.1487, 285.1824, 291.0985, 294.1380, 301.7380, 303.0493	M+H	POS	Flavonoids
**M0505**	Afzelin	6.165517	$C_{21}H_{20}O_{10}$	431.0979864	311.0557, 348.9552, 363.1813, 427.0168, 427.0236, 427.1730, 430.1995, 430.2796, 430.9889, 431.0978	M-H, $_2$M-H	NEG	Flavonoids
**M0506**	3,4,5-Trimethoxycinnamic acid	6.210617	$C_{12}H_{14}O_5$	221.0805434		M+H-H_2O	POS	Phenylpropanoids

**M0507**	2'-O-Methylisoliquiritigenin	6.210617	$C_{16}H_{14}O_4$	271.0959436		M+H	POS	Flavonoids
**M0508**	Celosin L	6.206317	$C_{47}H_{74}O_{20}$	939.4608975		M-H_2O-H	NEG	Steroids
**M0509**	Asaraldehyde	6.246033	$C_{10}H_{12}O_4$	391.1397935		$_2$M-H	NEG	Others
**M0510**	Dehydrocorydaline	6.2713	$C_{22}H_{24}NO_4$+	389.1582093		M+H, M+Na	POS	Alkaloids
**M0511**	Hispidol	6.29245	$C_{15}H_{10}O_4$	255.0646141	209.1164, 209.2377, 209.2571, 219.2121, 227.0693, 232.0661, 237.0541, 237.1097, 237.1458, 255.0642	M+H	POS	Flavonoids
**M0512**	Viridicatol	6.3331	$C_{15}H_{11}NO_3$	254.0806577		M+H	POS	Alkaloids
**M0513**	3-epi-Padmatin	6.3331	$C_{16}H_{14}O_7$	301.0699444		M+H-H_2O	POS	Flavonoids
**M0514**	Moluccanin	6.3331	$C_{20}H_{18}O_8$	387.1064954		M+H, M+Na	POS	Phenylpropanoids
**M0515**	Epoxymicheliolide	6.353367	$C_{15}H_{20}O_4$	287.1247394		M+Na	POS	Terpenes
**M0516**	Pinocembrin-7-O-beta-D-glucopyranoside	6.348833	$C_{21}H_{22}O_9$	463.1243672		M+FA-H	NEG	Flavonoids
**M0517**	Tilianin	6.348833	$C_{22}H_{22}O_{10}$	491.1188238		M+FA-H	NEG	Flavonoids
**M0518**	10-Deacetylbaccatin III	6.348833	$C_{29}H_{36}O_{10}$	525.2150462		M-H_2O-H	NEG	Terpenes
**M0519**	Medioresil	6.37365	$C_{21}H_{24}O_7$	371.1477732		M+H-H_2O	POS	Phenylpropanoids
**M0520**	3-Methoxybenzenepropanoic acid	6.4124	$C_{10}H_{12}O_3$	359.1501035		$_2$M-H	NEG	Carboxylic acid and derivatives
**M0521**	Methyl isoeugenol	6.4329	$C_{11}H_{14}O_2$	223.0977351		M+FA-H	NEG	Phenylpropanoids
**M0522**	Andropanolide	6.434233	$C_{20}H_{30}O_5$	373.1977506		M+Na	POS	Terpenes
**M0523**	Isosakuranin	6.4329	$C_{22}H_{24}O_{10}$	493.1349372		M+FA-H	NEG	Flavonoids
**M0524**	Padmatin	6.4329	$C_{16}H_{14}O_7$	635.1410314		$_2$M-H	NEG	Flavonoids
**M0525**	Ethyl Caffeic acid	6.455633	$C_{11}H_{12}O_4$	209.0806498		M+H	POS	Phenylpropanoids
**M0526**	Andrographidine C	6.455633	$C_{23}H_{24}O_{10}$	461.1427809		M+H	POS	Flavonoids
**M0527**	Medicagenic acid-3-O-beta-D-glucuronic acid-28-O-beta-D-glucopyranoside	6.455633	$C_{42}H_{64}O_{17}$	841.4198671		M+H	POS	Terpenes
**M0528**	Pseudoaspidin	6.4738	$C_{25}H_{32}O_8$	505.2081588		M+FA-H	NEG	Phenols
**M0529**	Carvacrol	6.516567	$C_{10}H_{14}O$	133.1011749	91.0546, 95.3318, 105.0699, 107.7716, 108.9293, 113.0696, 118.0771, 131.0859, 133.0641, 133.1012	M+H-H_2O	POS	Terpenes
**M0530**	Methyl p-coumarate	6.536933	$C_{10}H_{10}O_3$	161.059572		M+H-H_2O	POS	Phenylpropanoids

**M0531**	2-Hydroxyoctanoic acid	6.599233	$C_8H_{16}O_3$	159.1026176		M-H	NEG	Carboxylic acid and derivatives
**M0532**	2-Hydroxy-3-(hydroxymethyl)anthraquinone	6.621467	$C_{15}H_{10}O_4$	253.0505643	204.9890, 209.1186, 209.1544, 223.0401, 225.0563, 232.9832, 251.0352, 252.5909, 252.9885, 253.0506	M-H	NEG	Quinones
**M0533**	Silybin B	6.63925	$C_{25}H_{22}O_{10}$	465.1171236		M+H-H_2O	POS	Flavonoids
**M0534**	4-Methoxycinnamic acid	6.662667	$C_{10}H_{10}O_3$	355.1185433		$_2$M-H	NEG	Phenylpropanoids
**M0535**	Ginsenoside Rf	6.72075	$C_{42}H_{72}O_{14}$	823.4803011	298.9829, 305.2063, 326.6171, 365.1044, 365.1591, 435.3246, 451.9413, 453.3338, 603.6137, 823.4809	M+Na	POS	Terpenes
**M0536**	2-Butenoic acid, 2-methyl-, (3aS,4R,6E,10R,11aR)-2,3,3a,4,5,8,9,10,11,11a-decahydro-6,10-bis(hydroxymethyl)-3-methylene-2-oxocyclodeca[b]furan-4-yl ester, (2Z)-	6.746917	$C_{20}H_{28}O_6$	345.1704403		M-H_2O-H	NEG	Others
**M0537**	Ginsenoside Ra2	6.767467	$C_{58}H_{98}O_{26}$	1255.631964		M+FA-H	NEG	Terpenes
**M0538**	Naringenin	6.767467	$C_{15}H_{12}O_5$	271.0609642	250.9950, 251.0149, 253.0499, 253.1442, 269.0223, 270.1791, 270.8002, 270.9818, 271.0062, 271.0612	M-H	NEG	Flavonoids
**M0539**	Echinatin	6.782733	$C_{16}H_{14}O_4$	271.0957723		M+H	POS	Flavonoids
**M0540**	Genistein	6.842083	$C_{15}H_{10}O_5$	271.0594461	240.0765, 241.0483, 253.0506, 253.0742, 253.0847, 253.1801, 253.1946, 269.1003, 270.2789, 271.0600	M+H	POS	Flavonoids
**M0541**	Ginsenoside Rb1	6.884233	$C_{54}H_{92}O_{23}$	1131.590526	372.3840, 408.3503, 560.2607, 637.8237, 753.1785, 789.4752, 797.8080, 1071.0828, 1131.1219, 1131.5912	M+H, M+NH_4, M+Na	POS	Terpenes
**M0542**	Alismoxide	6.884233	$C_{15}H_{26}O_2$	221.1898009		M+H-H_2O	POS	Terpenes
**M0543**	3-O-Beta-D-Glucopyranosylplatycodigenin	6.884233	$C_{36}H_{58}O_{12}$	705.3832315		M+Na	POS	Terpenes

**M0544**	11-Deoxymogroside IIIE	6.884233	$C_{48}H_{82}O_{18}$	929.5459272		M+H-H_2O	POS	Terpenes
**M0545**	Neoanhydropodophyllol	6.921983	$C_{22}H_{24}O_7$	383.150475	285.0781, 307.0975, 308.0934, 310.2035, 323.1277, 347.1299, 365.1402, 365.1888, 383.0694, 383.1496	M+H-H_2O	POS	Phenylpropanoids
**M0546**	Matairesinol	6.952683	$C_{20}H_{22}O_6$	357.1341292	280.1110, 288.9350, 289.1060, 299.0552, 313.1444, 324.1006, 336.9896, 342.1133, 357.0012, 357.1344	M-H	NEG	Phenylpropanoids
**M0547**	Kaempferol	6.972433	$C_{15}H_{10}O_6$	267.0298767		M-H_2O-H	NEG	Flavonoids
**M0548**	p-hydroxy-5,6-dehydrokawain	7.013533	$C_{14}H_{12}O_4$	243.0666028		M-H	NEG	Phenols
**M0549**	Oroxylin A 7-O-beta-D-glucuronide methyl ester	7.036	$C_{23}H_{22}O_{11}$	473.1085819	311.0541, 311.3713, 311.4567, 354.7549, 460.4041, 465.6165, 472.2473, 472.2591, 473.0328, 473.1086	M-H	NEG	Flavonoids
**M0550**	Notoginsenoside Fc	7.055283	$C_{58}H_{98}O_{26}$	1255.631996		M-H, M+FA-H	NEG	Terpenes
**M0551**	7-Hydroxyflavone	7.055283	$C_{15}H_{10}O_3$	237.0555169		M-H	NEG	Flavonoids
**M0552**	Brusatol	7.066533	$C_{26}H_{32}O_{11}$	543.1834576		M+Na	POS	Terpenes
**M0553**	Ftaxilide	7.075933	$C_{16}H_{15}NO_3$	268.0978377		M-H	NEG	Carboxylic acid and derivatives
**M0554**	Moracin P	7.075933	$C_{19}H_{18}O_5$	371.1137262		M+FA-H	NEG	Phenols
**M0555**	Ginsenoside Rc	7.096283	$C_{53}H_{90}O_{22}$	1123.591351	621.4392, 637.2763, 694.0309, 698.1306, 783.4887, 810.8220, 915.5291, 945.5411, 1077.5852, 1123.6053	M-H, M+FA-H	NEG	Terpenes
**M0556**	Pratol	7.13965	$C_{16}H_{12}O_4$	313.0716654		M+FA-H	NEG	Flavonoids
**M0557**	Cirsiliol	7.1601	$C_{17}H_{14}O_7$	329.0663672		M-H	NEG	Flavonoids
**M0558**	6-Methoxytricin	7.1679	$C_{18}H_{16}O_8$	361.0908722		M+H	POS	Flavonoids
**M0559**	Syringetin	7.18005	$C_{17}H_{14}O_8$	345.0615577		M-H	NEG	Flavonoids
**M0560**	7 8-dihydroxy-4-phenylcoumarin	7.210967	$C_{15}H_{10}O_4$	255.0646084		M+H	POS	Phenylpropanoids
**M0561**	Ginsenoside Rg2	7.221417	$C_{42}H_{72}O_{13}$	829.4966631	637.4327, 773.5099, 783.4904, 783.6716, 828.9122, 829.3555, 829.3741, 829.4836, 829.4972, 829.5219	M-H, M+FA-H	NEG	Terpenes
**M0562**	Ingenol	7.242017	$C_{20}H_{28}O_5$	347.1861957		M-H	NEG	Terpenes

**M0563**	20(R)-Ginsenoside Rg2	7.2514	$C_{42}H_{72}O_{13}$	807.4850866	190.5561, 199.6856, 207.9150, 207.9377, 215.8781, 349.1100, 410.6642, 481.3679, 756.3838, 807.4877	M+Na	POS	Terpenes
**M0564**	Ginsenoside Rb3	7.312217	$C_{53}H_{90}O_{22}$	1101.579957	408.1638, 419.9768, 467.2613, 699.8947, 752.1962, 789.4753, 789.6476, 799.1071, 866.3530, 1101.5822	M+Na	POS	Terpenes
**M0565**	Picfeltarraenin X	7.332433	$C_{36}H_{54}O_{11}$	645.3622891		M+H-H_2O	POS	Terpenes
**M0566**	Proscillaridin A	7.352883	$C_{30}H_{42}O_8$	513.2859917		M+H-H_2O	POS	Steroids
**M0567**	Tenuifolin	7.392683	$C_{36}H_{56}O_{12}$	663.3725045		M+H-H_2O	POS	Terpenes
**M0568**	Physcion	7.4544	$C_{16}H_{12}O_5$	285.0749567		M+H	POS	Quinones
**M0569**	Angelol A	7.4895	$C_{20}H_{24}O_7$	357.1341362		M-H_2O-H	NEG	Phenylpropanoids
**M0570**	Deoxylimonin	7.494917	$C_{26}H_{30}O_7$	472.2318821		M+NH_4	POS	Terpenes
**M0571**	Adrenosterone	7.510117	$C_{19}H_{24}O_3$	345.170429		M+FA-H	NEG	Steroids
**M0572**	(20R)-Ginsenoside Rh1	7.515167	$C_{36}H_{62}O_9$	661.4263802		M+Na	POS	Terpenes
**M0573**	BlumeatinB	7.532167	$C_{17}H_{16}O_7$	313.0716611		M-H_2O-H	NEG	Flavonoids
**M0574**	Pseudoginsenoside RT1	7.532167	$C_{47}H_{74}O_{18}$	925.4805746		M-H	NEG	Terpenes
**M0575**	Kudinoside D	7.532167	$C_{47}H_{72}O_{17}$	953.4764134		M+FA-H	NEG	Alkaloids
**M0576**	Chrysosplenol D	7.537817	$C_{18}H_{16}O_8$	361.0908119		M+H	POS	Flavonoids
**M0577**	Anhydrosecoisolariciresinol	7.57305	$C_{20}H_{24}O_5$	343.1551026		M-H	NEG	Phenylpropanoids
**M0578**	11-Beta-hydroxyandrostenedione	7.57305	$C_{19}H_{26}O_3$	347.1859978		M+FA-H	NEG	Steroids
**M0579**	3,7,4'-Trihydroxy-5-methoxy-8-prenylflavanone	7.621433	$C_{21}H_{22}O_6$	371.1480326		M+H	POS	Flavonoids
**M0580**	Benzoin	7.6984	$C_{14}H_{12}O_2$	257.0818459		M+FA-H	NEG	Others
**M0581**	Resibufagin	7.6984	$C_{24}H_{30}O_5$	795.4098501		$_2$M-H	NEG	Steroids
**M0582**	Monocaprylin	7.718983	$C_{11}H_{22}O_4$	217.1444343		M-H	NEG	Others
**M0583**	Deacetylpseudolaric acid A	7.739467	$C_{20}H_{26}O_5$	345.1706448	301.1445, 304.9853, 311.0804, 324.9922, 327.1569, 327.2169, 344.9840, 344.9977, 345.0161, 345.1694	M-H	NEG	Terpenes
**M0584**	Ganoderic acid N	7.739467	$C_{30}H_{42}O_8$	575.2838567		M+FA-H	NEG	Terpenes
**M0585**	Xanthatin	7.76425	$C_{15}H_{18}O_3$	247.1325607		M+H	POS	Terpenes
**M0586**	Pterosin B	7.759583	$C_{14}H_{18}O_2$	263.1287675		M+FA-H	NEG	Terpenes
**M0587**	Isoformononetin	7.76425	$C_{16}H_{12}O_4$	307.0358807		M+K	POS	Flavonoids

**M0588**	13-Dehydroxyindaconintine	7.76425	$C_{34}H_{47}NO_9$	596.322311		M+H-H_2O	POS	Alkaloids
**M0589**	19alpha-Hydroxyasiatic acid	7.80075	$C_{30}H_{48}O_6$	549.3440419		M+FA-H	NEG	Terpenes
**M0590**	Betulin	7.807383	$C_{30}H_{50}O_2$	443.3878516		M+H	POS	Terpenes
**M0591**	Withaferin A	7.807383	$C_{28}H_{38}O_6$	493.2556188		M+Na	POS	Steroids
**M0592**	Arjungenin	7.82765	$C_{30}H_{48}O_6$	487.3406519		M+H-H_2O	POS	Terpenes
**M0593**	Ethyl ferulic acid	7.863683	$C_{12}H_{14}O_4$	221.0818227		M-H	NEG	Phenylpropanoids
**M0594**	5,7,4'-Trihydroxy-8-Methylflavanone	7.884033	$C_{16}H_{14}O_5$	285.0765091		M-H	NEG	Flavonoids
**M0595**	Chikusetsusaponin Iva	7.884033	$C_{42}H_{66}O_{14}$	793.437788	107.5669, 108.2446, 141.8551, 173.8598, 236.5047, 352.3419, 416.7030, 421.5094, 631.3932, 793.4368	M-H	NEG	Terpenes
**M0596**	3-oxo-2-pentylcyclopentaneacetic acid	7.988867	$C_{12}H_{20}O_3$	195.1377123	159.1166, 163.0388, 172.9769, 177.0543, 177.0906, 177.1268, 177.1636, 195.0791, 195.1011, 195.1373	M+H-H_2O, M+H	POS	Others
**M0597**	Lucidenic acid B	7.987883	$C_{27}H_{38}O_7$	473.254435	209.2332, 269.0423, 274.1148, 305.1343, 353.0660, 387.2400, 405.2645, 427.2496, 433.2474, 473.2540	M-H	NEG	Terpenes
**M0598**	3-Hydroxycapric acid	8.093783	$C_{10}H_{20}O_3$	187.1338362	171.0140, 186.9292, 186.9812, 186.9992, 187.0197, 187.0413, 187.0607, 187.0975, 187.1237, 187.1337	M-H	NEG	Carboxylic acid and derivatives
**M0599**	Cirsimaritin	8.093783	$C_{17}H_{14}O_6$	313.0714494	272.9814, 272.9974, 283.0235, 292.9835, 298.0494, 305.1508, 312.1920, 312.2234, 312.9946, 313.0714	M-H	NEG	Flavonoids
**M0600**	Cimicidanol-3-O-alpha-L-arabinoside	8.115767	$C_{35}H_{52}O_9$	661.3583119		M+FA-H	NEG	Terpenes
**M0601**	Phillygenin	8.1318	$C_{21}H_{24}O_6$	355.1527913		M+H-H_2O	POS	Phenylpropanoids
**M0602**	Sophoflavescenol	8.1996	$C_{21}H_{20}O_6$	367.1184429	337.0717, 339.1228, 346.9959, 348.9954, 351.0880, 352.0955, 367.0029, 367.0091, 367.0338, 367.1184	M-H	NEG	Flavonoids
**M0603**	Gypenoside XVII	8.1996	$C_{48}H_{82}O_{18}$	991.5495824		M+FA-H	NEG	Terpenes

**M0604**	Picfeltarraenin IA	8.260817	$C_{41}H_{62}O_{13}$	807.4182338	512.8293, 537.3600, 583.3702, 627.3579, 631.3845, 639.2938, 645.3594, 719.3856, 793.4367, 807.4185	M+FA-H	NEG	Terpenes
**M0605**	Picfeltarraenin IV	8.260817	$C_{47}H_{72}O_{18}$	969.4700026	113.0239, 133.0140, 150.1191, 287.3800, 321.0822, 339.0928, 497.1136, 616.9694, 909.5021, 969.4713	M+FA-H	NEG	Terpenes
**M0606**	Dihydromethysticin	8.295867	$C_{15}H_{16}O_5$	259.0960309		M+H-H_2O	POS	Others
**M0607**	Deoxyelephantopin	8.3249	$C_{19}H_{20}O_6$	389.124243		M+FA-H	NEG	Terpenes
**M0608**	Triptonide	8.366033	$C_{20}H_{22}O_6$	357.134365	288.9357, 293.2080, 316.9818, 329.2334, 336.9918, 339.1241, 349.5273, 356.2517, 356.9969, 357.1346	M-H	NEG	Terpenes
**M0609**	Negletein	8.379133	$C_{16}H_{12}O_5$	285.0750166	253.6417, 267.0637, 270.0507, 273.0501, 273.1475, 282.0573, 284.2982, 284.5599, 285.0400, 285.0750	M+H	POS	Flavonoids
**M0610**	Soyasaponin Bb	8.386533	$C_{48}H_{78}O_{18}$	941.513526	205.0717, 206.2343, 302.0146, 315.5117, 331.2549, 478.6680, 510.7289, 772.0140, 843.3837, 941.5131	M-H	NEG	Terpenes
**M0611**	Pimpinellin	8.419967	$C_{13}H_{10}O_5$	229.0491294		M+H-H_2O	POS	Phenylpropanoids
**M0612**	Rhein	8.449933	$C_{15}H_8O_6$	283.0246965	255.0298, 255.1643, 257.0453, 262.9892, 262.9947, 265.1808, 268.0376, 282.9786, 283.0247, 283.0348	M-H, $_2$M-H	NEG	Quinones
**M0613**	Dihydrokavain	8.481283	$C_{14}H_{16}O_3$	215.1065177		M+H-H_2O	POS	Others
**M0614**	Rhamnazin	8.49105	$C_{17}H_{14}O_7$	311.0560138		M-H_2O-H	NEG	Flavonoids
**M0615**	Micheliolide	8.56415	$C_{15}H_{20}O_3$	271.1297874		M+Na	POS	Terpenes
**M0616**	TMC-58B	8.584317	$C_{25}H_{26}N_2O_3$	425.1824111	395.3290, 401.0207, 406.3541, 407.1731, 407.3306, 412.6768, 422.9948, 424.1678, 424.3645, 425.1833	M+Na, M+K, M+H, M+H-H_2O	POS	Alkaloids
**M0617**	4',5-Dihydroxyflavone	8.728817	$C_{15}H_{10}O_4$	255.0644677		M+H	POS	Flavonoids
**M0618**	Rosmanol	8.745117	$C_{20}H_{26}O_5$	345.1706956		M-H	NEG	Terpenes
**M0619**	Soysaponin II	8.745117	$C_{47}H_{76}O_{17}$	911.5014457		M-H, M+FA-H	NEG	Terpenes
**M0620**	Butylparaben	8.765783	$C_{11}H_{14}O_3$	193.0869728		M-H	NEG	Phenols
**M0621**	Ononetin	8.83105	$C_{15}H_{14}O_4$	259.0959462		M+H	POS	Phenols
**M0622**	Pinocembrin	9.00685	$C_{15}H_{12}O_4$	255.0659846		M-H	NEG	Flavonoids

**M0623**	26-Deoxyactein	9.033267	$C_{37}H_{56}O_{10}$	683.37559		M+Na	POS	Terpenes
**M0624**	Galangin	9.070933	$C_{15}H_{10}O_5$	269.0455393	228.9887, 229.0117, 240.0437, 248.9720, 248.9963, 254.0216, 268.1636, 268.7818, 268.9834, 269.0454	M-H	NEG	Flavonoids
**M0625**	Pennogenin 3-O-beta-chacotrioside	9.070933	$C_{45}H_{72}O_{17}$	865.4593128		M-H_2O-H	NEG	Steroids
**M0626**	Caffeic acid phenethyl ester	9.116583	$C_{17}H_{16}O_4$	307.093401		M+H, M+Na	POS	Phenols
**M0627**	Pinobanksin 3-acetate	9.116583	$C_{17}H_{14}O_6$	315.0854521		M+H	POS	Flavonoids
**M0628**	Lathyrol	9.155033	$C_{20}H_{30}O_4$	333.2069918	312.9876, 312.9939, 315.1496, 315.1573, 315.1960, 332.9962, 333.0018, 333.1310, 333.1661, 333.2069	M-H	NEG	Terpenes
**M0629**	[6]-Gingerol	9.175717	$C_{17}H_{26}O_4$	293.1758324	241.0886, 252.9892, 272.9961, 278.5331, 292.9802, 292.9856, 293.0223, 293.0825, 293.1382, 293.1756	M-H	NEG	Phenols
**M0630**	Glyasperin C	9.28255	$C_{21}H_{24}O_5$	357.1686738		M+H	POS	Flavonoids
**M0631**	Deoxypodophyllotoxin	9.3236	$C_{22}H_{22}O_7$	399.142689	292.1054, 299.1613, 322.3469, 341.1204, 350.1127, 363.1232, 381.1328, 381.3138, 398.6822, 399.1405	M+H, M+Na, M+NH_4	POS	Phenylpropanoids
**M0632**	Semilicoisoflavone B	9.343017	$C_{20}H_{16}O_6$	351.0871709	330.9956, 333.0778, 336.0632, 350.1989, 350.3315, 350.3675, 350.9844, 350.9953, 351.0011, 351.0868	M-H	NEG	Flavonoids
**M0633**	Uralenol	9.344083	$C_{20}H_{18}O_7$	353.1010164		M+H-H_2O	POS	Flavonoids
**M0634**	Tetrahydrocurcumin	9.344083	$C_{21}H_{24}O_6$	355.1527546		M+H-H_2O	POS	Phenols
**M0635**	Irisolidone	9.386483	$C_{17}H_{14}O_6$	313.0716482		M-H	NEG	Flavonoids
**M0636**	Alisol C	9.407033	$C_{30}H_{46}O_5$	531.3325766		M+FA-H	NEG	Terpenes
**M0637**	Polygalic acid	9.427383	$C_{29}H_{44}O_6$	533.3119259		M+FA-H	NEG	Terpenes
**M0638**	3-Oxo-7-hydroxychol-4-enoic acid	9.447883	$C_{24}H_{36}O_4$	433.2596852		M+FA-H	NEG	Steroids
**M0639**	Xanthohumol D	9.46905	$C_{21}H_{22}O_6$	371.1483365		M+H	POS	Flavonoids
**M0640**	Dihydrocurcumin	9.509867	$C_{21}H_{22}O_6$	369.1341774		M-H	NEG	Phenylpropanoids
**M0641**	Delta3,2-Hydroxylbakuchiol	9.532467	$C_{18}H_{24}O_2$	273.1843432		M+H, M+Na	POS	Phenols
**M0642**	8-Prenylnaringenin	9.553117	$C_{20}H_{20}O_5$	339.1236501		M-H	NEG	Flavonoids

**M0643**	4-Methyloctanoic acid	9.594467	$C_9H_{18}O_2$	315.253679		$_2$M-H	NEG	Carboxylic acid and derivatives
**M0644**	3'-O-Methylbatatasin III	9.6144	$C_{16}H_{18}O_3$	259.1324148		M+H	POS	Phenylpropanoids
**M0645**	Ombuin	9.61505	$C_{17}H_{14}O_7$	311.0560723		M-H_2O-H	NEG	Flavonoids
**M0646**	3alpha,12beta-Dihydroxycholanoic acid	9.63565	$C_{24}H_{40}O_4$	437.2914266		M+FA-H	NEG	Steroids
**M0647**	Prosaikogenin F	9.696717	$C_{36}H_{58}O_8$	663.4120612		M+FA-H	NEG	Terpenes
**M0648**	Licochalcone C	9.717517	$C_{21}H_{22}O_4$	337.1444742		M-H	NEG	Flavonoids
**M0649**	Zederone	9.738317	$C_{15}H_{18}O_3$	229.1219986		M+H-H_2O	POS	Terpenes
**M0650**	Licochalcone A	9.738317	$C_{21}H_{22}O_4$	339.1580592		M+H	POS	Flavonoids
**M0651**	Ethyl cinnamate	9.819283	$C_{11}H_{12}O_2$	194.1174222		M+NH_4	POS	Phenylpropanoids
**M0652**	Dihydrojasmone	9.839717	$C_{11}H_{18}O$	149.1323522		M+H-H_2O	POS	Others
**M0653**	Lupiwighteone	9.839717	$C_{20}H_{18}O_5$	339.1216442		M+H	POS	Flavonoids
**M0654**	Oleanolic acid 3-glycosides	9.843267	$C_{47}H_{76}O_{16}$	941.5103607		M+FA-H	NEG	Terpenes
**M0655**	Curcumin	9.86075	$C_{21}H_{20}O_6$	369.1323137	305.0798, 318.0867, 319.0938, 320.1036, 323.1254, 333.1112, 333.2007, 351.1215, 351.3029, 369.1307	M+H, M+K, M+Na	POS	Phenols
**M0656**	3-Hydroxydodecanoic acid	9.8866	$C_{12}H_{24}O_3$	215.165191	204.3832, 208.8812, 211.1739, 214.9925, 215.0135, 215.0341, 215.0546, 215.0916, 215.1295, 215.1646	M-H	NEG	Carboxylic acid and derivatives
**M0657**	Flavonol	9.8866	$C_{15}H_{10}O_3$	283.0610388		M+FA-H	NEG	Flavonoids
**M0658**	Neoglycyrol	9.8866	$C_{21}H_{18}O_6$	365.1028615	324.9962, 341.2393, 344.9749, 344.9811, 344.9942, 350.0750, 358.9760, 364.9825, 364.9919, 365.1022	M-H	NEG	Phenylpropanoids
**M0659**	Forskolin	9.901633	$C_{22}H_{34}O_7$	433.2211506		M+Na	POS	Terpenes
**M0660**	Qingyangshengenin A	9.9482	$C_{49}H_{72}O_{17}$	977.4744955		M+FA-H	NEG	Steroids
**M0661**	Emodin	9.968867	$C_{15}H_{10}O_5$	269.0455027	182.9872, 211.1756, 225.0557, 225.1859, 228.9893, 248.9977, 251.2001, 268.2001, 268.9865, 269.0454	M-H	NEG	Quinones
**M0662**	Mosloflavone	9.965233	$C_{17}H_{14}O_5$	299.0906502		M+H	POS	Flavonoids
**M0663**	Argininyl-fructosyl-glucose	0.795917	$C_{18}H_{34}N_4O_{12}$	499.2233269	360.1681, 362.9886, 379.0992, 405.0258, 409.0052, 419.2129, 470.9669, 481.2119, 498.9989, 499.2236	M+H	POS	

**M0664**	arginin	0.831683	$C_6H_{14}N_4O_2$	213.0744522	185.0289, 192.0547, 195.0640, 196.0478, 196.0693, 203.0399, 210.0680, 210.9685, 212.8527, 213.0743	M+K	POS
**M0665**	asparagin	0.866033	$C_4H_8N_2O_3$	131.0460589	101.0241, 105.0158, 107.3384, 113.0240, 113.0353, 114.0197, 115.0034, 129.5784, 131.0347, 131.0457	M-H_2O-H, M-H	NEG
**M0666**	Neokestose	0.904983	$C_{18}H_{32}O_{16}$	549.1668124	432.7932, 443.1368, 450.7992, 450.9069, 503.1613, 548.6800, 548.7753, 548.9030, 549.0597, 549.1655	M-H, M+FA-H	NEG
**M0667**	panose	0.905317	$C_{18}H_{32}O_{16}$	543.1308167	497.0074, 504.9468, 507.9234, 538.9625, 539.8956, 539.9611, 539.9713, 539.9852, 542.9752, 543.1312	M+K	POS
**M0668**	hexuronate	0.92405	$C_6H_{10}O_7$	193.0349941	133.0142, 147.5618, 148.9495, 149.0449, 157.0136, 160.6434, 163.0245, 175.0245, 181.8022, 193.0350	M-H	NEG
**M0669**	isolychnose	0.962317	$C_{24}H_{42}O_{21}$	705.1835137	399.0817, 463.0887, 489.1691, 525.1205, 533.1467, 533.6569, 534.1556, 543.1314, 608.1603, 705.1842	M+K	POS
**M0670**	3,4-dihydroxyoxolan-2-one	0.974217	$C_4H_6O_4$	235.0455253	161.0455, 171.0300, 189.0397, 190.9939, 191.0559, 199.0242, 207.2464, 217.0352, 234.9839, 235.0456	M-H, $_2$M-H	NEG
**M0671**	dehydroascorbates	0.994317	$C_6H_6O_6$	154.9984339	111.0800, 113.0000, 116.7260, 126.9881, 127.0396, 137.0232, 138.4286, 141.2356, 150.1105, 154.9986	M-H_2O-H	NEG
**M0672**	Daucic acid	1.030217	$C_7H_8O_7$	249.0247488	159.0296, 161.0094, 167.0346, 191.0195, 204.9813, 214.7467, 230.9601, 231.0336, 248.9707, 249.0250	M+FA-H	NEG
**M0673**	sesamose	1.0832	$C_{24}H_{42}O_{21}$	689.2092246	437.1221, 463.0764, 479.0435, 509.1469, 517.6505, 518.0795, 527.1573, 557.1149, 573.0984, 689.2083	M+Na	POS

**M0674**	(R)-Methyl 2-(pyrrolidin-2-YL)acetate	1.212567	$C_7H_{13}NO_2$	144.1017552	94.6829, 99.0440, 111.5760, 116.0697, 118.8994, 126.0787, 128.1683, 130.0972, 144.0894, 144.1016	M+H	POS
**M0675**	PCG	1.211617	$C_{10}H_{12}N_5O_7P$	344.0395365	208.2394, 208.5754, 208.7066, 208.9725, 209.0875, 209.1090, 209.8332, 246.0600, 254.0288, 344.0396	M-H	NEG
**M0676**	Dehydroshikimic acid	1.272283	$C_7H_8O_5$	217.0350763	187.1089, 198.9339, 199.0246, 199.8500, 216.8524, 216.9086, 216.9442, 216.9818, 217.0148, 217.0352	M+FA-H	NEG
**M0677**	DL-Pyroglutamic acid	1.272283	$C_5H_7NO_3$	257.077638	213.0400, 213.0883, 239.0637, 240.0501, 240.0725, 251.6296, 256.9801, 257.0303, 257.0696, 257.0753	$_2$M-H	NEG
**M0678**	5'-GMP;5'-guanosine monophosphate	1.292683	$C_{10}H_{14}N_5O_8P$	362.0504615	220.6297, 227.9734, 237.3457, 240.5313, 256.5367, 285.3956, 285.4396, 294.4678, 356.4279, 362.0504	M-H	NEG
**M0679**	Sarmentosine; Sarmentosine (glycoside)	1.3112	$C_{11}H_{17}NO_7$	320.0982504	258.0429, 264.3096, 282.0825, 284.0857, 302.0505, 302.0845, 305.1929, 320.0043, 320.0637, 320.0988	M+FA-H	NEG
**M0680**	agarobiose dimethylacetal	1.495383	$C_{14}H_{26}O_{11}$	415.1460282		M+FA-H	NEG
**M0681**	DL-Isoleucine	1.515783	$C_6H_{13}NO_2$	130.0873035		M-H	NEG
**M0682**	hexulose	1.555033	$C_6H_{12}O_6$	161.0455252	133.0503, 142.9252, 143.0347, 159.0298, 160.8413, 160.8923, 160.9219, 160.9352, 161.0244, 161.0455	M-H_2O-H	NEG
**M0683**	Acetoin glucoside	1.57535	$C_{10}H_{20}O_7$	297.118824	252.9417, 253.0343, 253.0714, 254.1142, 279.0508, 296.9646, 296.9901, 297.0240, 297.0587, 297.1179	M+FA-H	NEG
**M0684**	Glucogallin beta	1.5957	$C_{13}H_{16}O_{10}$	331.0669157	213.0016, 225.9574, 241.0353, 271.0452, 285.0989, 313.0574, 316.4334, 328.3174, 329.5426, 331.0666	M-H	NEG
**M0685**	Galactomannan	1.5957	$C_{18}H_{32}O_{16}$	503.1619134		M-H	NEG
**M0686**	radicamine a	1.8385	$C_{12}H_{17}NO_5$	273.143944		M+NH_4	POS

**M0687**	Scandoside	1.858683	$C_{16}H_{22}O_{11}$	413.1041534	284.0465, 301.1371, 306.0835, 323.1123, 351.1009, 395.0939, 412.5962, 413.0027, 413.0966, 413.1044	M+Na, M+K, M+NH$_4$, M+H-H$_2$O	POS
**M0688**	3,5-dihydroxy-6-methyl-2,3-dihydropyran-4-one	1.979633	$C_6H_8O_4$	145.0494477	127.1307, 127.8805, 129.7678, 131.0412, 131.1019, 133.0274, 134.0234, 138.8670, 142.5493, 145.0495	M+H	POS
**M0689**	5-Hydroxy-7,8,4'-trimethoxyflavone	10.00652	$C_{18}H_{16}O_6$	329.1011533	268.0725, 285.0763, 293.2111, 295.2278, 296.0672, 297.1039, 299.0556, 313.0699, 314.0777, 329.1010	M+H, M+Na	POS
**M0690**	Gingerglycolipid A	10.00998	$C_{33}H_{56}O_{14}$	721.3662032	599.0011, 675.2427, 675.3593, 676.3834, 710.6966, 714.6442, 721.0292, 721.0414, 721.0527, 721.3563	M+FA-H	NEG
**M0691**	2-(Acetoxymethyl)-3-(methoxycarbonyl)biphenylene	10.02702	$C_{17}H_{14}O_4$	283.0957749	241.8827, 247.2409, 250.0622, 253.9156, 259.8934, 265.0853, 265.1779, 265.2130, 282.2783, 283.0956	M+H, M+Na	POS
**M0692**	Isolongifolen-9-one	10.05245	$C_{15}H_{22}O$	263.1652586	208.5543, 214.9935, 215.9876, 219.1742, 222.9799, 223.0001, 242.9850, 262.9922, 263.1330, 263.1647	M+FA-H	NEG
**M0693**	(1R,3R)-3-[(E)-3-Methoxy-2-methyl-3-oxo-1-propenyl]-2,2-dimethylcyclopropanecarboxylic acid (S)-3-(2-butenyl)-2-methyl-4-oxo-2-cyclopenten-1-yl	10.21697	$C_{21}H_{28}O_5$	405.1917177	344.9900, 345.1704, 351.9873, 364.9872, 364.9922, 384.9932, 384.9983, 404.9954, 405.0046, 405.1915	M+FA-H	NEG
**M0694**	Dehydrosasurealactone	10.27852	$C_{15}H_{20}O_2$	277.1443903	228.9884, 233.1544, 233.1801, 236.9964, 239.9805, 256.9810, 266.7825, 275.5518, 276.9877, 277.1442	M+FA-H	NEG
**M0695**	hovenicacid	10.36207	$C_{30}H_{48}O_5$	533.3485421		M+FA-H	NEG
**M0696**	9,10-Dihydroxystearic acid	10.42268	$C_{18}H_{36}O_4$	299.2573135		M+H-H$_2$O	POS
**M0697**	9-acetoxythymol 3-O-tiglate	10.61337	$C_{17}H_{22}O_4$	289.1445413		M-H	NEG

**M0698**	Juniperic acid	10.65502	$C_{16}H_{32}O_3$	271.2278024	228.8535, 230.9857, 250.9906, 254.5519, 260.7305, 270.9785, 271.0026, 271.0546, 271.1165, 271.2276	M-H	NEG
**M0699**	Dibunol	10.73567	$C_{15}H_{24}O$	203.1792217	121.1013, 126.0552, 127.4084, 133.1010, 135.0020, 147.1167, 161.1324, 175.1481, 203.1452, 203.1793	M+H-H_2O	POS
**M0700**	Bonannione A	10.74012	$C_{25}H_{28}O_5$	407.1865776		M-H	NEG
**M0701**	Myricardiol	10.73567	$C_{30}H_{50}O_2$	443.3873521	403.2239, 407.3635, 425.3771, 442.7085, 443.1472, 443.1539, 443.2136, 443.2749, 443.2836, 443.3866	M+H	POS
**M0702**	(+/-)9-Hode	10.8046	$C_{18}H_{32}O_3$	295.2276455	277.2170, 277.2537, 292.9129, 293.9437, 294.9841, 294.9926, 295.0007, 295.1436, 295.1621, 295.2274	M-H	NEG
**M0703**	ilekudinol A	10.8046	$C_{29}H_{42}O_4$	499.3070227		M+FA-H	NEG
**M0704**	12,13-epoxy-octadeca-9-enoic acid	10.81845	$C_{18}H_{32}O_3$	279.2311379		M+H-H_2O	POS
**M0705**	Hexahydropseudoionone	10.8989	$C_{13}H_{26}O$	243.1964624	216.1036, 222.9995, 225.1496, 225.6430, 242.9844, 243.0887, 243.1229, 243.1299, 243.1594, 243.1965	M+FA-H	NEG
**M0706**	Triptoditerpenic acid B	10.90253	$C_{21}H_{28}O_3$	346.236479	101.5730, 138.8799, 209.8200, 244.1699, 272.2377, 287.2668, 296.1828, 300.2315, 329.2062, 346.2369	M+NH_4	POS
**M0707**	Sterol	11.07097	$C_{17}H_{28}O$	293.2122005	125.5491, 126.7246, 128.5726, 157.0121, 185.1169, 249.2242, 265.2175, 275.2009, 292.9842, 293.2119	M+FA-H	NEG
**M0708**	Ganoderiol G	11.07097	$C_{31}H_{52}O_5$	485.3636679		M-H_2O-H	NEG
**M0709**	kakispyrone	11.10155	$C_{20}H_{28}O_4$	331.1914194	291.1086, 302.1247, 302.6606, 305.1944, 327.8823, 330.9851, 330.9902, 330.9982, 331.1009, 331.1915	M-H	NEG
**M0710**	panacon	11.10155	$C_{36}H_{62}O_8$	667.4435589	387.6583, 486.5377, 491.2836, 517.2611, 550.1696, 647.0038, 667.0109, 667.0229, 667.3217, 667.4427	M+FA-H	NEG

**M0711**	2,4-diethyl-7,7-dimethylcyclohepta-1,3,5-triene	11.1537	$C_{13}H_{20}$	177.1635771		M+H	POS
**M0712**	Tridecanone	11.19607	$C_{13}H_{26}O$	243.1964049	197.1907, 202.9936, 208.2251, 208.6538, 225.1490, 242.9860, 243.0866, 243.1234, 243.1599, 243.1959	M+FA-H	NEG
**M0713**	cangoronine	11.1967	$C_{30}H_{44}O_5$	507.307136	189.5897, 205.5008, 208.0205, 208.7985, 305.2504, 346.1891, 350.9348, 353.9389, 449.2643, 507.3083	M+Na	POS
**M0714**	Dulxanthone C	11.27603	$C_{25}H_{28}O_6$	405.1705251	233.6936, 235.2282, 238.8886, 305.1478, 305.1975, 353.6528, 404.9940, 405.0086, 405.0677, 405.1706	M-H_2O-H	NEG
**M0715**	Coronarsaeure	11.38577	$C_{18}H_{32}O_3$	297.24159	216.0262, 221.1902, 233.2256, 243.2099, 251.2363, 261.2200, 279.2314, 297.0896, 297.0948, 297.2434	M+H	POS
**M0716**	Tetradecanel	11.6603	$C_{14}H_{28}O$	257.212113		M+FA-H	NEG
**M0717**	octadeca-9,12-dienol	12.0878	$C_{18}H_{34}O$	267.2676376	153.8566, 172.9771, 189.7804, 196.5214, 214.8102, 239.1471, 249.2578, 267.1229, 267.1578, 267.2679	M+H	POS
**M0718**	3-Hydroxypalmitic acid	12.15093	$C_{16}H_{32}O_3$	271.2276946	225.2211, 234.5668, 239.0325, 253.2166, 270.9819, 271.0225, 271.0560, 271.0627, 271.1941, 271.2278	M-H	NEG
**M0719**	16-Heptadecenal	12.41633	$C_{17}H_{32}O$	297.2432494		M+FA-H	NEG
**M0720**	ursolicacid lactone	12.4642	$C_{30}H_{48}O_3$	455.3529081	238.8018, 240.6469, 247.8785, 256.8445, 300.5226, 301.8125, 305.1524, 386.8952, 455.0067, 455.3533	M-H, M+FA-H	NEG
**M0721**	24,25-Dihydroxylanost-7,9(11)-dien-3-one	12.70553	$C_{30}H_{48}O_3$	479.3484461	243.3883, 245.5762, 248.3049, 282.0702, 298.9356, 333.5496, 339.1577, 423.7131, 479.2236, 479.3491	M+Na	POS
**M0722**	10,12-hexadecadienyl acetate	12.75007	$C_{18}H_{32}O_2$	279.2326613	237.1195, 238.9932, 243.7883, 251.0298, 259.0013, 278.9845, 279.0028, 279.0085, 279.1660, 279.2328	M-H	NEG

**M0723**	Neokadsuranic acid B	12.89632	$C_{30}H_{44}O_3$	453.3355818	317.1952, 322.2503, 381.2043, 398.6331, 411.3323, 435.3286, 436.3717, 453.2014, 453.2093, 453.3371	M+H	POS
**M0724**	2-Heptadecanone	13.23402	$C_{17}H_{34}O$	299.2588298		M+FA-H	NEG
**M0725**	2-Nonadecanone	13.3195	$C_{19}H_{38}O$	327.2900658		M+FA-H	NEG
**M0726**	octadec-7-enoic acid	13.50207	$C_{18}H_{34}O_2$	281.2482992	243.7900, 243.8211, 260.9956, 265.0266, 277.3134, 280.9496, 280.9783, 280.9834, 281.0049, 281.2482	M-H, M+FA-H	NEG
**M0727**	3,4-dihydroxy-3-methyloxolan-2-one	1.999783	$C_5H_8O_4$	115.0392208	90.9476, 91.0514, 92.9484, 96.2282, 97.0286, 97.0646, 104.2653, 107.9653, 113.9631, 115.0392	M+H-H_2O	POS
**M0728**	Homoarecoline	2.019517	$C_9H_{15}NO_2$	170.1173378		M+H	POS
**M0729**	3,4-Dihydroxybenzyl alcohol-4-glucoside	2.025833	$C_{13}H_{18}O_8$	347.0979189	279.1094, 288.0615, 288.0976, 301.0921, 303.0321, 346.1631, 346.8829, 346.9493, 347.0211, 347.0994	M+FA-H	NEG
**M0730**	γ-glutamyl-valine	2.042017	$C_{10}H_{18}N_2O_5$	247.1283014	229.0660, 229.1188, 230.1017, 237.0275, 237.9827, 246.6318, 247.0134, 247.0565, 247.0770, 247.1285	M+H	POS
**M0731**	4-Hydroxybenzoic acid glucoside	2.149783	$C_{13}H_{16}O_8$	345.0824606	277.1229, 287.0748, 299.0661, 299.0768, 299.1168, 305.1459, 344.9110, 345.0231, 345.0424, 345.0815	M-H, M+FA-H	NEG
**M0732**	protoanemonin hydrate glucoside	2.170183	$C_{11}H_{16}O_8$	257.0667019	225.0405, 228.9595, 239.0572, 256.9156, 256.9238, 256.9883, 256.9962, 257.0292, 257.0346, 257.0693	M-H_2O-H	NEG
**M0733**	Cardiospermin	2.227933	$C_{11}H_{17}NO_7$	298.0891148	262.0717, 262.1165, 274.9837, 280.0807, 280.1129, 280.1243, 281.1549, 292.9948, 298.0009, 298.0890	M+Na	POS
**M0734**	3-hydroxyphenylglycine	2.380833	$C_8H_9NO_3$	212.056548	182.0449, 191.3079, 195.2202, 200.6440, 207.1563, 208.7217, 209.4601, 211.9418, 212.0310, 212.0561	M+FA-H	NEG
**M0735**	securiterpenoside	2.401467	$C_{11}H_{18}O_8$	323.0982502		M+FA-H	NEG

**M0736**	aucubigenin	2.57055	$C_9H_{12}O_4$	183.0662692	139.0690, 139.0763, 158.6891, 165.0195, 183.0047, 183.0117, 183.0294, 183.0362, 183.0491, 183.0659	M-H	NEG
**M0737**	S-(-)-Carbidopa	2.61515	$C_{10}H_{14}N_2O_4$	227.1022109	204.0281, 204.5290, 206.5305, 209.0919, 209.1155, 215.5349, 219.0169, 226.9855, 227.0512, 227.1025	M+H, M+Na	POS
**M0738**	Cyclo(Pro-Ala)	2.654733	$C_8H_{12}N_2O_2$	213.0881534	168.9394, 169.0138, 169.0502, 169.0981, 177.9029, 184.9034, 211.2568, 211.2737, 213.0399, 213.0889	M+FA-H	NEG
**M0739**	Cyclo(Ala-Pro-)	2.655617	$C_8H_{12}N_2O_2$	151.086532		M+H-H_2O	POS
**M0740**	tuberostemospironine	2.903283	$C_{13}H_{19}NO_4$	254.1380285	226.1176, 230.9943, 236.0551, 236.1011, 236.1282, 249.0053, 254.0111, 254.0659, 254.1102, 254.1386	M+H	POS
**M0741**	koaburaside	2.907783	$C_{14}H_{20}O_9$	377.1088957	212.2919, 248.6124, 265.5182, 309.1174, 320.9444, 325.9135, 331.1023, 341.1081, 349.2640, 377.0869	M+FA-H	NEG
**M0742**	cornoside_qt	3.053017	$C_8H_{10}O_3$	199.0612061		M+FA-H	NEG
**M0743**	(2S)-2alpha-Hydroxy-4H-1,4-benzoxazine-3(2H)-one	3.108183	$C_8H_7NO_3$	166.0497442	140.0330, 142.9668, 143.9965, 148.0385, 148.0744, 149.0228, 149.0593, 163.0385, 165.9830, 166.0495	M+H	POS
**M0744**	N-Demethyldoryphornine	3.1779	$C_{10}H_9NO_3$	236.0564226	168.0274, 190.9271, 192.0665, 197.8264, 207.9758, 210.2079, 215.2464, 235.9699, 236.0285, 236.0562	M+FA-H	NEG
**M0745**	6β-hydroxyhuperzine a	3.209433	$C_{15}H_{18}N_2O_2$	276.1700044	248.9480, 252.9444, 253.8820, 258.1206, 258.1523, 259.1436, 276.0522, 276.0857, 276.1207, 276.1700	M+NH_4	POS
**M0746**	hexadienedial	3.262267	$C_6H_6O_2$	109.0295029	66.0349, 76.9811, 80.8584, 84.4367, 89.5696, 105.4277, 106.1561, 107.3083, 108.0213, 109.0294	M-H	NEG
**M0747**	6-O-caffeoyl-D-glucopyranose	3.303033	$C_{15}H_{18}O_9$	387.0930245		M+FA-H	NEG

**M0748**	Cornoside	3.344067	$C_{14}H_{20}O_8$	361.1138564	293.9673, 294.4705, 295.6973, 315.0080, 315.1080, 325.7513, 360.9590, 360.9651, 361.0665, 361.1104	M+FA-H	NEG
**M0749**	3-Acetylbutyric Acid	3.3876	$C_6H_{10}O_3$	175.0610642		M+FA-H	NEG
**M0750**	Vanilloloside	3.4133	$C_{14}H_{20}O_8$	339.104022	233.7710, 264.0499, 266.9829, 280.8300, 308.9106, 316.5573, 321.0150, 321.0567, 338.8720, 339.1050	M+Na	POS
**M0751**	Methyl 6-O-galloyl-b-D-glucopyranoside	3.490267	$C_{14}H_{18}O_{10}$	345.0823108	285.0591, 301.0963, 301.1511, 303.4874, 322.9855, 330.0606, 337.6395, 344.1818, 345.0370, 345.0815	M-H	NEG
**M0752**	Veratramide	3.594833	$C_9H_{11}NO_3$	180.0665194		M-H	NEG
**M0753**	Feretoside	3.597733	$C_{17}H_{24}O_{11}$	427.1199163	409.1088, 415.3027, 424.2147, 424.8662, 424.9094, 424.9228, 426.2390, 426.7300, 427.0128, 427.1203	M+Na, M+K, $M+NH_4$	POS
**M0754**	(2s,6 ζ)-3,7-dimethyloct-3(10)-ene-1,2,6,7-tetrol1-o-β-d-glucopyranoside	3.678517	$C_{16}H_{30}O_9$	411.1869739	252.6614, 270.8721, 316.0204, 343.1013, 344.7136, 365.1811, 411.0922, 411.0981, 411.1428, 411.1920	M+FA-H	NEG
**M0755**	bidensyneoside c	3.721683	$C_{16}H_{22}O_8$	387.1294012	287.1087, 299.7794, 316.5793, 341.1134, 342.1223, 343.1008, 352.1494, 369.1770, 380.9744, 387.0867	M+FA-H	NEG
**M0756**	Monomethyl glutarate	3.7624	$C_6H_{10}O_4$	191.0560843		M+FA-H	NEG
**M0757**	Chlorogensaure	3.7624	$C_{16}H_{18}O_9$	353.0876402	229.9483, 248.6253, 274.0724, 275.1354, 285.0294, 285.0608, 286.6171, 317.0786, 352.7125, 353.0875	M-H	NEG
**M0758**	opuntioside	3.805317	$C_{13}H_{18}O_9$	299.0770256	253.0947, 255.0430, 267.5327, 269.8453, 285.1772, 298.1403, 298.1756, 298.9065, 299.0393, 299.0768	$M-H_2O-H$	NEG
**M0759**	harpagide acetate	3.845733	$C_{17}H_{26}O_{10}$	389.1452207	345.1777, 389.0483, 389.0533, 389.0641, 389.0702, 389.1038, 389.1123, 389.1362, 389.1420, 389.1531	M-H	NEG
**M0760**	Grandoside	3.845733	$C_{17}H_{32}O_{11}$	457.1930016		M+FA-H	NEG

**M0761**	Catechin 7-glucoside	3.898717	$C_{21}H_{24}O_{11}$	453.1378163	435.1252, 452.4929, 452.5052, 452.5569, 452.5738, 452.7337, 452.8916, 452.9154, 453.1321, 453.1427	M+H, M+Na	POS
**M0762**	Yuheinoside	3.9067	$C_{16}H_{24}O_9$	405.1403577	337.1109, 344.9748, 356.0869, 359.1343, 360.1379, 361.1480, 365.4220, 387.0710, 405.0451, 405.1395	M+FA-H	NEG
**M0763**	(2R,3R,4R,5S,6R)-2-[(2R,3R)-2-(3,4-dihydroxyphenyl)-5,7-dihydroxy-chroman-3-yl]oxy-6-(hydroxymethyl)tetrahydropyran-3,4,5-triol	3.9067	$C_{21}H_{24}O_{11}$	497.1294726	451.1233, 451.1856, 451.2264, 461.1633, 479.1210, 496.2467, 496.9888, 497.0018, 497.1346, 497.1437	M+FA-H	NEG
**M0764**	1-O-(E)-caffeoyl-β-D-gentiobiose	3.9067	$C_{21}H_{28}O_{14}$	503.1405283	347.6986, 443.1198, 454.1028, 457.0457, 467.1181, 479.9305, 489.0343, 502.2520, 503.0589, 503.1414	M-H	NEG
**M0765**	Trimethylcitrate	3.949733	$C_9H_{14}O_7$	215.0560392		M-H_2O-H	NEG
**M0766**	quercetin-3-o-α-arabinopyranosyl(1'''→6'')-β-glucopyranoside	4.010017	$C_{32}H_{38}O_{21}$	757.1840506	431.1174, 437.7267, 451.1230, 462.0799, 487.6448, 510.3964, 517.1694, 595.1302, 625.1276, 757.1830	M-H	NEG
**M0767**	Isosalsoline	4.038933	$C_{11}H_{15}NO_2$	211.1439239		M+NH_4	POS
**M0768**	4-Carboxycarbostyril	4.059117	$C_{10}H_7NO_3$	190.0497558	158.0348, 162.0547, 163.0264, 163.1227, 167.9636, 173.0803, 173.1317, 184.7719, 188.3518, 190.0494	M+H	POS
**M0769**	3,4-dimethoxyphenolβ-d-apiofuranosyl(1→6)-β-d-glucopyranoside	4.059117	$C_{19}H_{28}O_{12}$	466.1900623		M+NH_4	POS
**M0770**	1-o-β-d-glucopyranosylamplexin	4.0702	$C_{16}H_{26}O_9$	361.1500873	292.9982, 293.1244, 293.9671, 299.0882, 316.9959, 317.0648, 317.1014, 317.1609, 361.0584, 361.1507	M-H	NEG
**M0771**	4-hydroxy-2 hexenoic acid	4.109367	$C_6H_{10}O_3$	175.0610624	157.0144, 157.0504, 171.0922, 174.4746, 174.9283, 174.9561, 174.9978, 175.0247, 175.0325, 175.0610	M+FA-H	NEG

**M0772**	Glucosyringicacid	4.1305	$C_{15}H_{20}O_{10}$	341.0875096	203.0338, 208.6192, 208.8606, 211.1898, 221.0448, 251.0558, 281.0663, 295.0782, 323.0756, 341.0849	M-H_2O-H	NEG
**M0773**	curculigin	4.150867	$C_{23}H_{28}O_{12}$	495.1506613	415.1023, 449.2399, 451.1248, 465.1393, 477.0684, 477.1407, 477.1611, 495.0690, 495.1503, 495.1667	M-H, M+FA-H	NEG
**M0774**	2-(3,4-Dihydroxybenzoyloxy)-4,6-dihydroxyphenylacetic acid	4.171183	$C_{15}H_{12}O_{8}$	301.0351296	177.0186, 185.0607, 187.0394, 213.0199, 213.0549, 257.0457, 267.3423, 300.9651, 300.9981, 301.0345	M-H_2O-H	NEG
**M0775**	(3S,5R,6R,7E,9S)-megastigman-7-ene-3,5,6,9-tetrol-9-o-β-d-glucopyranoside	4.171183	$C_{19}H_{34}O_{9}$	451.2183413	313.0592, 331.0801, 335.1231, 336.1068, 353.0869, 405.2123, 407.1453, 451.0850, 451.1299, 451.2175	M+FA-H	NEG
**M0776**	pikuroside	4.171183	$C_{23}H_{30}O_{14}$	529.156087	281.5787, 290.3797, 348.5218, 353.0834, 375.0144, 493.1630, 513.4384, 517.1365, 529.1432, 529.1517	M-H	NEG
**M0777**	Syringin	4.1801	$C_{17}H_{24}O_{9}$	395.130571	283.1290, 324.1887, 332.0070, 364.1119, 377.0758, 377.1290, 377.1819, 394.7133, 394.7205, 395.1323	M+Na	POS
**M0778**	p-Coumaroyl-b-D-glucose	4.191417	$C_{15}H_{18}O_{8}$	325.0926509	281.0452, 281.0671, 289.0698, 299.0549, 307.0813, 325.0148, 325.0463, 325.0517, 325.0578, 325.0920	M-H	NEG
**M0779**	4'-o-methyl leucopelargonidin-3-mono-glucofuranoside	4.191417	$C_{22}H_{26}O_{11}$	511.1452538	153.0193, 165.0550, 169.0145, 179.0549, 179.0708, 214.7637, 313.0560, 375.0688, 494.9211, 511.1462	M+FA-H	NEG
**M0780**	(1S,2S)-1,2-Di(2-furyl)-1,2-ethanediol	4.233283	$C_{10}H_{10}O_{4}$	239.0561215	196.0391, 209.0453, 211.0247, 221.0088, 221.0448, 223.0279, 224.0328, 238.8918, 239.0194, 239.0558	M+FA-H	NEG
**M0781**	epigeoside	4.233283	$C_{27}H_{34}O_{16}$	595.1663302	457.1150, 462.0828, 474.5695, 475.1250, 487.1278, 505.1379,	M-H_2O-H	NEG

					510.6559, 549.2529, 577.1625, 595.1672		
**M0782**	peruvianoside iii	4.233283	$C_{33}H_{40}O_{21}$	771.2000152	462.0803, 563.1751, 605.1902, 609.1438, 625.1462, 639.1551, 641.1653, 725.2275, 733.1738, 771.1991	M-H	NEG
**M0783**	(1R,2R,3S)-3-methylcyclopentane-1,2-diol	4.25365	$C_6H_{12}O_2$	161.0818886	133.0507, 142.9252, 143.0348, 159.0303, 160.8921, 160.9350, 161.0242, 161.0454, 161.0604, 161.0817	M+FA-H	NEG
**M0784**	2,3,4,9-Tetrahydro-1H-beta-carboline-3-carboxylic acid	4.261817	$C_{12}H_{12}N_2O_2$	217.097117	188.0703, 189.0539, 199.0956, 199.1472, 200.0702, 200.1282, 205.0555, 206.0412, 217.0464, 217.0962	M+H	POS
**M0785**	3- Ferulylquinic acid	4.2945	$C_{17}H_{20}O_9$	367.1030846	193.0504, 209.3251, 212.4441, 213.0407, 215.0401, 237.5337, 259.2699, 280.1929, 339.8371, 367.1042	M-H	NEG
**M0786**	7-Glucosyl quercetin	4.314383	$C_{21}H_{20}O_{12}$	509.0935441		M+FA-H	NEG
**M0787**	madreselvin A	4.314383	$C_{28}H_{32}O_{17}$	639.1569463	476.0174, 476.0956, 476.1704, 477.1039, 519.1144, 543.1137, 561.1222, 593.1506, 621.1116, 639.1566	M-H, M+FA-H	NEG
**M0788**	1,2,3,9-tetrahydropyrrolo(2,1-b)quinazolin-1-carboxylicacid	4.334717	$C_{12}H_{12}N_2O_2$	261.0879901	217.0155, 217.0512, 217.0980, 218.0817, 219.0773, 235.0612, 243.0769, 261.0048, 261.0414, 261.0881	M+FA-H	NEG
**M0789**	(2s,3s)-1-phenyl-2,3-butanediol3-o-β-d-glucopyranoside	4.334717	$C_{16}H_{24}O_7$	373.150172		M+FA-H	NEG
**M0790**	3,5,7-trihydroxychromone	4.35425	$C_9H_6O_5$	239.0197809		M+FA-H	NEG
**M0791**	4-Hydroxy-2-methoxybenzoicacid	4.38625	$C_8H_8O_4$	151.0389148		M+H-H_2O	POS
**M0792**	tectoruside	4.397	$C_{21}H_{30}O_{13}$	489.1614144		M-H	NEG

**M0793**	benzyl alcoholβ-d-(2'-o-β-xylopyranosyl)glucopyranoside	4.406467	$C_{18}H_{26}O_{10}$	425.1411646	353.2340, 375.6470, 385.7654, 389.1705, 404.2053, 407.0320, 407.1802, 424.7268, 425.0517, 425.1414	M+H-H_2O, M+NH_4, M+Na	POS
**M0794**	6-O-acetyl shanzhiside methyl ester	4.417383	$C_{19}H_{28}O_{12}$	447.1506755	285.0719, 293.0879, 305.1477, 341.0721, 401.1439, 401.2061, 447.0858, 447.0918, 447.1423, 447.1479	M-H	NEG
**M0795**	(6s,9r)-vomifoliol-9-o-β-xylopyranosyl-(1"→6')-o-β-glucopyranoside	4.437767	$C_{24}H_{38}O_{12}$	563.2344693	353.0663, 383.0762, 385.1845, 387.1659, 443.0970, 495.1520, 517.1504, 517.2275, 563.1389, 563.2277	M+FA-H	NEG
**M0796**	(?)-olivil-4"-o-β-d-glucopyranoside	4.437767	$C_{26}H_{34}O_{12}$	583.203124	327.1233, 345.1331, 351.1288, 357.1353, 375.1454, 386.2886, 446.5791, 537.1952, 583.1062, 583.2114	M+FA-H	NEG
**M0797**	8-hydroxy-10-hydrosweroside	4.448483	$C_{16}H_{24}O_{10}$	399.1270635		M+Na	POS
**M0798**	5,7-Dihydroxy-4-oxo-4H-chromene-2-carboxylic acid	4.45805	$C_{10}H_6O_6$	221.0091148	180.0700, 186.2171, 191.0353, 193.0493, 198.8437, 203.0351, 206.0232, 208.1243, 208.1471, 221.0088	M-H	NEG
**M0799**	northalifoline(tautomericstructure 1)	4.468667	$C_{10}H_{11}NO_3$	194.0810831	162.0625, 163.0388, 163.0741, 164.0703, 166.0859, 167.0343, 176.0701, 177.0542, 194.0509, 194.0805	M+H	POS
**M0800**	1-ribityl-2,3-diketo-1,2,3,4-tetrahydro-6,7-dimethyl-quinoxaline	4.48885	$C_{15}H_{20}N_2O_6$	325.1387547	281.1330, 289.1162, 290.1043, 293.0796, 294.0952, 303.0491, 307.1255, 308.1308, 325.0349, 325.1300	M+H	POS
**M0801**	Yadanzioside I	4.48885	$C_{29}H_{38}O_{16}$	665.2040835	347.0934, 409.1868, 433.1353, 500.1462, 503.1502, 545.7974, 546.0474, 664.3400, 664.8303, 665.2047	M+NH_4, M+Na	POS
**M0802**	sinapaldehyde glucoside	4.508883	$C_{17}H_{22}O_9$	353.1223708	222.1255, 254.1196, 303.0486, 333.0533, 342.0587, 352.2939, 352.6820, 353.0292, 353.0825, 353.1223	M+H-H_2O	POS

**M0803**	2-methyl-2,3-dihydropyran-6-one	4.5189	$C_6H_8O_2$	157.0505131	116.9956, 118.6510, 122.3118, 127.0038, 132.1375, 137.0036, 139.0776, 156.8942, 157.0118, 157.0504	M+FA-H	NEG
**M0804**	3-O-p-coumaroylquinic acid	4.5189	$C_{16}H_{18}O_8$	337.0928603	258.0768, 269.0996, 275.0937, 275.1024, 293.0327, 293.0667, 293.1025, 301.0836, 319.0928, 337.0930	M-H	NEG
**M0805**	ningpogenin	4.5404	$C_9H_{14}O_3$	215.0924098	197.0819, 205.9779, 212.5147, 214.7623, 214.9803, 214.9996, 215.0163, 215.0333, 215.0539, 215.0923	M+FA-H	NEG
**M0806**	glehlinoside c	4.560383	$C_{26}H_{32}O_{13}$	551.1778617	389.1772, 431.1343, 447.1440, 461.1456, 491.1519, 503.8181, 507.1768, 550.2605, 550.2700, 551.1772	M-H	NEG
**M0807**	5'-hydroxyiso-muronulatol-2',5'-di-O-glucoside	4.560383	$C_{29}H_{38}O_{16}$	687.2148027	501.1595, 519.1721, 535.1660, 593.1870, 611.1976, 619.2244, 623.1953, 625.1418, 641.2079, 687.2115	M+FA-H	NEG
**M0808**	beta-Hydroxypropiosyringone	4.57055	$C_{11}H_{14}O_5$	227.0909912		M+H	POS
**M0809**	Cyclo(Pro-Val)	4.580733	$C_{10}H_{16}N_2O_2$	241.1193143	198.1138, 207.8603, 208.4880, 208.4982, 208.5996, 213.0555, 240.9809, 241.0345, 241.0708, 241.1193	M+FA-H	NEG
**M0810**	doryphornine	4.601033	$C_{11}H_{11}NO_3$	250.0719709	206.0820, 207.0300, 207.0661, 208.4620, 208.4707, 215.9870, 230.9627, 232.0614, 237.8809, 250.0716	M+FA-H	NEG
**M0811**	paeonilactone b	4.6104	$C_{10}H_{12}O_4$	197.0806404	151.0387, 151.0750, 151.1112, 161.0595, 161.0961, 169.0862, 169.1333, 179.0699, 179.1047, 197.0813	M+H	POS
**M0812**	(3R,4S)-3-(4-hydroxy-3-methoxy-benzyl)chroman-3,4,7-triol	4.6104	$C_{17}H_{18}O_6$	319.1169362	245.0449, 261.1301, 273.0408, 273.0762, 301.0348, 301.0700, 301.1101, 310.6617, 319.0432, 319.0800	M+H	POS
**M0813**	6'-o-e-feruloylmonotropein	4.64985	$C_{26}H_{30}O_{14}$	549.1592794		M+H-H_2O	POS

**M0814**	Glycolophenone	4.6621	$C_8H_8O_2$	181.050515		M+FA-H	NEG
**M0815**	Methyl p-hydroxybenzoate glucoside	4.6818	$C_{14}H_{18}O_8$	295.0820166		M-H_2O-H	NEG
**M0816**	Flavoplatycoside	4.6818	$C_{27}H_{32}O_{16}$	593.1497451	473.1471, 479.1530, 525.1595, 547.1344, 547.1513, 547.2531, 547.2723, 592.3041, 593.1520, 593.1610	M-H_2O-H	NEG
**M0817**	2-Hex-3-enoxy-6-(hydroxymethyl)oxane-3,4,5-triol	4.701583	$C_{12}H_{22}O_6$	261.1341377		M-H	NEG
**M0818**	mineoside	4.701583	$C_{25}H_{30}O_{13}$	583.1670069		M+FA-H	NEG
**M0819**	Indolizin	4.7092	C_8H_7N	118.0653006	107.6347, 109.3504, 111.7951, 112.1123, 112.2456, 116.0577, 116.2508, 117.5307, 117.8106, 118.0654	M+H	POS
**M0820**	zivulgarin_qt	4.7092	$C_{22}H_{22}O_{10}$	447.1277174		M+H	POS
**M0821**	Rehmaionoside B	4.727383	$C_{19}H_{34}O_8$	413.2138651	250.9992, 251.1254, 269.0092, 367.0460, 373.0338, 377.0297, 395.0396, 395.1997, 413.0420, 413.2197	M+NH_4, M+Na	POS
**M0822**	feroxidin	4.742133	$C_{11}H_{14}O_3$	239.0923506		M+FA-H	NEG
**M0823**	Isohyenanchin	4.742133	$C_{15}H_{20}O_7$	623.2352409		$_2$M-H	NEG
**M0824**	C-Homoerythrinan, 1,6-didehydro-3,15,16-trimethoxy-, (3.beta.)-	4.747617	$C_{20}H_{27}NO_3$	368.1628041	305.0437, 323.0540, 324.1860, 332.1976, 341.0660, 350.1564, 350.2053, 351.0469, 351.0990, 368.1656	M+K	POS
**M0825**	3-methylcyclopentane-1,2-diol	4.7616	$C_6H_{12}O_2$	161.0817594		M+FA-H	NEG
**M0826**	Phenethyl rutinoside	4.766817	$C_{20}H_{30}O_{10}$	453.1730729		M+Na	POS
**M0827**	(2R)-2-butoxybutanedioic acid	4.783	$C_8H_{14}O_5$	189.0767381	148.4945, 156.0757, 161.0607, 164.7348, 171.0662, 179.5574, 180.8526, 181.4981, 188.9299, 189.0766	M-H_2O-H, M-H	NEG
**M0828**	Catalposide	4.80555	$C_{22}H_{26}O_{12}$	505.1331488		M+Na	POS
**M0829**	hemipholin	4.8229	$C_{21}H_{22}O_{10}$	433.1143456		M-H	NEG
**M0830**	4-O-Glucosyl-5-O-methyl-visamminol	4.825417	$C_{22}H_{28}O_{10}$	435.1634465		M+H-H_2O	POS

**M0831**	Dihydrofisetin	4.843383	$C_{15}H_{12}O_6$	269.0450159	239.0343, 240.0426, 241.0496, 250.9821, 257.0443, 267.9343, 268.0388, 268.5987, 268.9910, 269.0453	M-H_2O-H	NEG
**M0832**	protosappanin c	4.843383	$C_{16}H_{14}O_6$	347.0769768		M+FA-H	NEG
**M0833**	syringetin-3-rutinoside	4.864317	$C_{29}H_{34}O_{17}$	653.1730751	187.0399, 189.0766, 285.0397, 287.0563, 301.0356, 463.0884, 473.1405, 652.6555, 652.7721, 653.1757	M-H	NEG
**M0834**	Apigenin 7-O-(2G-rhamnosyl)gentiobioside	4.86395	$C_{33}H_{40}O_{19}$	723.2119634		M+H-H_2O	POS
**M0835**	3a,4-Dihydro-2-benzofuran-1,3-dione	4.884717	$C_8H_6O_3$	195.0297104	154.9928, 165.0548, 175.0013, 177.0405, 180.0062, 180.0428, 194.8762, 194.9052, 194.9911, 195.0303	M+FA-H	NEG
**M0836**	(1s,2r,4s,7r)-vicodiol 2-o-β-d-glucopyrano-side	4.883433	$C_{16}H_{28}O_7$	315.1789333		M+H-H_2O	POS
**M0837**	apocynoside i	4.883433	$C_{19}H_{30}O_8$	409.1823103	375.1653, 391.2084, 406.4446, 406.4578, 407.1111, 407.1210, 408.6820, 409.1116, 409.1187, 409.1810	M+H, M+K, M+Na	POS
**M0838**	(E)-P-Coumaricacid	4.904733	$C_9H_8O_3$	163.0399878	119.3808, 119.6504, 120.0447, 135.0447, 142.9926, 145.5630, 147.0453, 162.8926, 163.0031, 163.0398	M-H, $_2$M-H	NEG
**M0839**	Viscumiside A	4.902817	$C_{22}H_{24}O_{11}$	447.1275095		M+H-H_2O	POS
**M0840**	quercetin-3-o-[(6-o-sinapoyl)-β-d-glucopyra-nosyl-(1→2)-β-d-galactopyranoside]	4.902817	$C_{38}H_{40}O_{21}$	833.2126619	547.2642, 600.7394, 635.4625, 832.3845, 832.4073, 832.7429, 832.8786, 832.9063, 833.0374, 833.2213	M+H, M+Na	POS
**M0841**	(4S)-2,3-dehydroleucocyanidin	4.9237	$C_{15}H_{12}O_7$	285.039095	244.5643, 245.0634, 257.0451, 257.1395, 264.9930, 267.0295, 267.1216, 282.8465, 284.5434, 285.0402	M-H_2O-H	NEG
**M0842**	Rehmaionoside	4.9237	$C_{19}H_{32}O_8$	433.2078718	297.1708, 313.0627, 327.0863, 369.1819, 387.2003, 389.0849, 389.1261, 433.0660, 433.1118, 433.2051	M+FA-H	NEG

**M0843**	A-D-xylopyranoside	4.9237	$C_{25}H_{26}O_{13}$	579.1359624	343.2100, 353.0967, 371.2057, 399.0951, 417.1042, 459.1272, 532.2485, 533.2593, 578.2618, 579.1354	M+FA-H	NEG
**M0844**	7-O-Methylmorroniside	4.944233	$C_{18}H_{28}O_{11}$	419.1557026		M-H	NEG
**M0845**	2alpha-Methylcyclopentanone	4.98065	$C_6H_{10}O$	116.1071843	84.5713, 86.0606, 88.0761, 98.4923, 99.0805, 102.2704, 108.1217, 115.0543, 116.0706, 116.1071	M+NH4	POS
**M0846**	Tricin-7-O-beta-D-lucopyranoside	4.98065	$C_{23}H_{24}O_{12}$	493.1327695		M+H-H2O, M+H	POS
**M0847**	quercetin-3-o-[(6-o-feruloyl)-β-d-glucopyra-nosyl-(1→2)-β-d-galactopyranoside]	4.98065	$C_{37}H_{38}O_{20}$	803.201662	440.6346, 465.1020, 501.1571, 565.1490, 802.3871, 802.4026, 802.8986, 802.9173, 803.1909, 803.2075	M+H, M+Na	POS
**M0848**	SALVIANOLICACID D	5.00415	$C_{20}H_{18}O_{10}$	417.084525		M-H	NEG
**M0849**	jioglutin E	5.020233	$C_{11}H_{20}O_5$	215.1275153		M+H-H2O	POS
**M0850**	scabrans G5_qt	5.024633	$C_{10}H_{10}O_4$	239.0559048		M+FA-H	NEG
**M0851**	schizonepetoside E	5.024633	$C_{16}H_{28}O_8$	329.1606918		M-H2O-H	NEG
**M0852**	16-hydroxytriptolide	5.059767	$C_{20}H_{24}O_7$	377.1581042	319.1351, 323.1268, 331.1520, 341.1377, 345.0712, 354.0766, 356.1275, 359.1483, 376.6749, 377.1564	M+H-H2O, M+H	POS
**M0853**	(-)-pinoresinol glucoside	5.065517	$C_{26}H_{32}O_{11}$	565.1927273	389.0740, 403.1333, 417.1177, 489.1750, 501.1747, 519.1826, 519.2794, 565.1262, 565.1552, 565.2233	M-H, M+FA-H	NEG
**M0854**	Dihydrodehydroconiferyl alcohol 4-O-glucoside	5.084733	$C_{26}H_{34}O_{11}$	567.2089977	385.1128, 405.1558, 417.1121, 491.1902, 503.1881, 521.1999, 521.2950, 549.2064, 550.2869, 567.2086	M+FA-H	NEG
**M0855**	Hydrangetin	5.105167	$C_{10}H_8O_4$	383.077285		2M-H	NEG
**M0856**	8β-(4'-hydroxytigloyloxy)-3β,14-dihydroxy-6βh,7αh-germacra-1(10)z,4 e,-11(13)-trien-6,12-olide	5.120067	$C_{20}H_{26}O_7$	401.1572715	351.1219, 353.1009, 356.1226, 368.1237, 369.1351, 371.1497, 373.1663, 383.1485, 401.1512, 401.1566	M+Na	POS
**M0857**	Myristicinaldehyd	5.125517	$C_9H_8O_4$	359.0774007		2M-H	NEG

**M0858**	2,3,5,4'-tetrahydroxystilbene-2-o-(6"-o-acetyl)-beta-d-glucopyranoside	5.125517	$C_{22}H_{24}O_{10}$	493.1346295	317.0278, 317.0661, 331.0418, 331.0812, 331.1237, 349.0593, 361.1648, 447.1895, 492.2772, 493.1378	M+FA-H	NEG
**M0859**	limocitrin-3-glucoside	5.125517	$C_{23}H_{24}O_{13}$	507.1146851		M-H	NEG
**M0860**	blumenol B	5.160017	$C_{13}H_{22}O_3$	227.1637928	163.1477, 165.1269, 167.0699, 167.1062, 173.1321, 189.1116, 191.1425, 209.1530, 227.1275, 227.1640	M+H	POS
**M0861**	Cyclo(Phe-Pro)	5.160017	$C_{14}H_{16}N_2O_2$	245.1280581		M+H	POS
**M0862**	delamide	5.160017	$C_{13}H_{16}N_2O_4$	247.1073597		M+H-H_2O	POS
**M0863**	2-hex-2-enoxy-6-(hydroxymethyl)oxane-3,4,5-triol	5.160017	$C_{12}H_{22}O_6$	285.1301886	258.0472, 267.0647, 267.1123, 267.1590, 267.2171, 270.0513, 285.0387, 285.0742, 285.0813, 285.1303	M+H-H_2O, M+NH_4, M+Na	POS
**M0864**	bruceoside e	5.165683	$C_{31}H_{42}O_{16}$	651.2287388		M-H_2O-H	NEG
**M0865**	(+)-4-Hexanolide	5.186017	$C_6H_{10}O_2$	159.066196		M+FA-H	NEG
**M0866**	Daphnetin-7-methylether	5.186017	$C_{10}H_8O_4$	237.0404953	207.7928, 207.8943, 208.0749, 209.5780, 216.9900, 219.0300, 219.1024, 236.5096, 236.9996, 237.0399	M+FA-H	NEG
**M0867**	Diosbulbin J	5.201533	$C_{19}H_{22}O_8$	379.1374092		M+H	POS
**M0868**	Araldite RD 4	5.208017	$C_8H_{12}O_2$	185.0817999		M+FA-H	NEG
**M0869**	villoside	5.208017	$C_{16}H_{26}O_8$	345.1554144	273.0789, 281.0418, 283.0609, 301.0356, 301.0717, 304.7108, 327.0526, 344.6541, 345.0611, 345.1551	M-H	NEG
**M0870**	angelitriol	5.221633	$C_{15}H_{18}O_6$	277.1061349		M+H-H_2O	POS
**M0871**	deca-6,8-diene-1,3,5-triol	5.228367	$C_{10}H_{18}O_3$	231.1236723	210.1795, 210.9818, 211.0202, 213.0769, 213.1130, 230.9888, 231.0295, 231.0549, 231.0676, 231.1234	M+FA-H	NEG
**M0872**	staphylionoside e	5.260967	$C_{19}H_{32}O_8$	389.2160798		M+H	POS
**M0873**	chrysoeriol 4'-o-β-d-glucopyranoside	5.260967	$C_{22}H_{22}O_{11}$	463.1223535		M+H	POS
**M0874**	hispidulin-7-o-glucuronide	5.260967	$C_{22}H_{20}O_{12}$	477.1018642	299.7643, 301.0081, 301.0697, 302.3832, 315.0862, 411.6736, 452.0626, 459.2768, 476.3304, 477.1019	M+H	POS

**M0875**	12,13-dihydroxyeuparin	5.280867	$C_{13}H_{14}O_5$	233.080511		M+H-H_2O	POS
**M0876**	staphylionoside d	5.280867	$C_{19}H_{30}O_8$	409.1822636	345.2368, 347.0611, 347.2201, 368.2968, 373.1973, 390.4716, 391.0826, 391.2097, 409.0856, 409.1818	M+NH_4, M+Na	POS
**M0877**	Dunnisinin	5.361633	$C_{11}H_{14}O_5$	227.0909929		M+H	POS
**M0878**	isololiolide	5.401083	$C_{11}H_{16}O_3$	197.1169526	155.0701, 161.0595, 161.0958, 161.1322, 169.0858, 179.0700, 179.1063, 179.1427, 197.0805, 197.1168	M+H, M+NH_4, M+H-H_2O	POS
**M0879**	Dimethylpimelinsaure	5.410867	$C_9H_{16}O_4$	187.097501	169.0869, 185.9285, 186.9300, 186.9561, 186.9815, 186.9995, 187.0402, 187.0600, 187.0663, 187.0974	M-H	NEG
**M0880**	6-Oxooctanoic acid	5.430967	$C_8H_{14}O_3$	203.0924367	159.0441, 159.1029, 175.0394, 182.9876, 185.0447, 185.0821, 202.9943, 203.0157, 203.0352, 203.0922	M+FA-H	NEG
**M0881**	Wallichoside	5.430967	$C_{20}H_{28}O_8$	377.1601901		M-H_2O-H	NEG
**M0882**	Cnidimol B	5.451283	$C_{15}H_{16}O_6$	291.0871845		M-H	NEG
**M0883**	(6R,9S)-vomifoliol	5.460883	$C_{13}H_{20}O_3$	225.1481358	189.1270, 190.0202, 190.5218, 199.0255, 201.5280, 207.0651, 207.1013, 207.1376, 225.1100, 225.1482	M+H, M+NH_4	POS
**M0884**	Illudin I	5.5009	$C_{15}H_{22}O_3$	251.1633051	215.1062, 215.1420, 229.0740, 233.0803, 233.1155, 233.1533, 251.0490, 251.0911, 251.1248, 251.1635	M+H	POS
**M0885**	Zedoarolide B	5.530817	$C_{15}H_{22}O_5$	281.1391766	243.1236, 243.1522, 243.1605, 245.1687, 276.5257, 281.0065, 281.0428, 281.0776, 281.1066, 281.1389	M-H	NEG
**M0886**	eupalinilide f	5.540483	$C_{20}H_{26}O_8$	417.1533313		M+Na	POS
**M0887**	(1r,2r)-p-menth-4(5)-ene-1,2-diol1-o-β-d-(6-o-acetyl)glucopyranoside	5.551367	$C_{18}H_{30}O_8$	355.175582		M-H_2O-H	NEG
**M0888**	isorinic acid	5.560217	$C_{18}H_{16}O_7$	367.0799037		M+Na	POS
**M0889**	crepidiaside A	5.560217	$C_{21}H_{26}O_9$	405.1531393		M+H-H_2O	POS

**M0890**	Martynoside	5.571717	$C_{31}H_{40}O_{15}$	651.2298645	400.6887, 400.9384, 475.1858, 503.1799, 505.1761, 515.2958, 549.1956, 562.9924, 588.5455, 651.2291	M-H, M+FA-H	NEG
**M0891**	sec-hydroxyaegineticacid	5.580267	$C_{15}H_{24}O_5$	307.1508102	246.9899, 253.1577, 263.1625, 271.1693, 285.1183, 289.1408, 289.1795, 290.1177, 290.2110, 307.1510	M+H-H_2O, M+Na	POS
**M0892**	Licorice glycoside A	5.6116	$C_{36}H_{38}O_{16}$	725.2106209	399.1067, 404.0335, 417.1191, 445.0754, 445.2071, 457.1712, 517.3135, 531.1517, 549.1595, 725.2059	M-H	NEG
**M0893**	dendroside e	5.639267	$C_{21}H_{36}O_8$	434.2737779		M+NH_4	POS
**M0894**	persicogenin-3'glucoside	5.639267	$C_{23}H_{26}O_{11}$	461.1432987		M+H-H_2O	POS
**M0895**	9-Hydroxythymol	5.713133	$C_{10}H_{14}O_2$	211.0975391		M+FA-H	NEG
**M0896**	Consume close grain	5.713133	$C_{16}H_{14}O_6$	283.0610508		M-H_2O-H	NEG
**M0897**	Aureusidin	5.720333	$C_{15}H_{10}O_6$	287.0540897	270.0499, 270.1477, 284.8172, 284.8494, 285.4528, 285.8354, 285.8438, 286.0460, 286.9981, 287.0541	M+H	POS
**M0898**	(3S,3aR,5S,6S,7aR)-5,6-dihydroxy-3,6-dimethyl-3,3a,4,5,7,7a-hexahydrobenzofuran-2-one	5.733467	$C_{10}H_{16}O_4$	199.0975792	178.9935, 179.0125, 181.0866, 198.5488, 198.9823, 199.0025, 199.0214, 199.0548, 199.0615, 199.0974	M-H	NEG
**M0899**	3-methyl-4-hydroxycinnamic acid	5.7405	$C_{10}H_{10}O_3$	161.0596214		M+H-H_2O	POS
**M0900**	Gibberellin A95	5.7405	$C_{19}H_{22}O_5$	331.1527297	301.1427, 313.0350, 313.0704, 313.1078, 313.1426, 313.1753, 331.0436, 331.0794, 331.1153, 331.1523	M+H	POS
**M0901**	mioporosidegenin	5.77375	$C_{12}H_{22}O_5$	245.1392865		M-H	NEG
**M0902**	borneol-2-o-β-d-apiofuranosyl(1→6)-β-d-glucopyranoside	5.794	$C_{21}H_{36}O_{10}$	493.2293171	409.2026, 436.1528, 447.2228, 451.3503, 492.1953, 492.2830, 492.2933, 492.7306, 493.1082, 493.2264	M-H, M+FA-H	NEG
**M0903**	(Z)-(1S,5R)-beta-pinen-10-yl-beta-vicianoside	5.814383	$C_{21}H_{34}O_{10}$	491.2134338	327.0857, 329.0341, 329.0656, 329.2319, 431.1005, 445.2076, 447.1424, 491.1328, 491.1441, 491.2120	M+FA-H	NEG

**M0904**	1,3,6-trihydroxy-2-methyl-9,10-anthra-quinone-3-o-(6'-o-acetyl)-beta-d-glucoside	5.822883	$C_{23}H_{22}O_{11}$	475.1222887	60.5694, 79.7423, 96.9501, 114.9763, 126.3220, 209.7425, 271.0592, 457.1743, 457.2556, 475.1252	M+H	POS
**M0905**	Ganoine	5.8347	$C_{11}H_{17}NO_2$	240.1243251	195.1383, 196.1340, 196.9843, 197.0600, 197.1178, 205.9785, 220.9840, 240.0157, 240.0416, 240.1242	M+FA-H	NEG
**M0906**	Dehydrovomifoliol	5.8347	$C_{13}H_{18}O_3$	267.1237588	239.0763, 247.0004, 248.9992, 249.1119, 252.0421, 266.9848, 267.0285, 267.0662, 267.1058, 267.1230	M+FA-H	NEG
**M0907**	Umckalin	5.883717	$C_{11}H_{10}O_5$	223.0596478		M+H	POS
**M0908**	clemaphenolA	5.9039	$C_{20}H_{22}O_6$	359.1475292	295.1310, 299.0533, 313.1053, 313.1423, 323.1268, 323.1817, 327.1185, 341.1376, 359.0732, 359.1470	M+H, M+Na	POS
**M0909**	fibraurin	5.8974	$C_{20}H_{20}O_7$	371.1134688		M-H_2O-H, M-H	NEG
**M0910**	dendronobiloside b	5.8974	$C_{21}H_{38}O_8$	463.255113	254.0579, 285.0396, 287.0484, 301.0333, 417.1193, 417.1949, 417.2484, 463.0869, 463.1794, 463.2606	M+FA-H	NEG
**M0911**	Lyoniresinol	5.9467	$C_{22}H_{28}O_8$	443.1684668		M+Na	POS
**M0912**	Acetylbenzoyl	5.961133	$C_9H_8O_2$	193.0505556		M+FA-H	NEG
**M0913**	ciliatoside a	5.967	$C_{36}H_{40}O_{19}$	759.2092305		M+H-H_2O	POS
**M0914**	(2R)-7-hydroxy-2-(4-hydroxyphenyl)chroman-4-one	5.987217	$C_{15}H_{12}O_4$	239.0697528		M+H-H_2O	POS
**M0915**	3'-Hydroxymelanettin	6.007433	$C_{16}H_{12}O_6$	301.0699908		M+H	POS
**M0916**	p-Methoxyhydrocinnamic acid	6.0222	$C_{10}H_{12}O_3$	179.071354		M-H	NEG
**M0917**	3-Acetonylcyclopentanone	6.042667	$C_8H_{12}O_2$	185.0818728	116.9284, 116.9956, 121.5985, 123.0812, 141.0919, 164.9967, 184.9850, 185.0038, 185.0446, 185.0813	M+FA-H	NEG
**M0918**	javanicolide c	6.042667	$C_{26}H_{36}O_{11}$	569.2241099	493.2072, 501.1945, 501.2738, 523.2202, 523.3524, 533.8752, 551.2272, 569.0612, 569.0746, 569.2161	M+FA-H	NEG

**M0919**	2,6,6-trimethyl-2-hydroxycyclohexanone	6.063067	$C_9H_{16}O_2$	201.1132275	184.7280, 198.0390, 199.8114, 200.3909, 200.5797, 200.9970, 201.0174, 201.0555, 201.0771, 201.1131	M+FA-H	NEG
**M0920**	n-trans-feruloylmethoxytyramine	6.068133	$C_{19}H_{21}NO_5$	344.148196	309.2056, 317.1743, 326.1093, 326.2020, 327.1217, 327.1591, 327.1665, 327.1985, 344.0798, 344.1489	M+H	POS
**M0921**	galanol B	6.068133	$C_{20}H_{32}O_6$	391.2085408	355.1169, 368.0204, 368.0617, 373.1236, 373.2004, 391.0370, 391.0422, 391.1377, 391.1442, 391.2102	M+Na	POS
**M0922**	Ammiol	6.083417	$C_{14}H_{12}O_6$	257.0454689		M-H_2O-H	NEG
**M0923**	Coronopolin	6.083417	$C_{15}H_{20}O_4$	263.1286753	236.0170, 242.9857, 245.0816, 245.1190, 262.9910, 263.0184, 263.0567, 263.0909, 263.1009, 263.1282	M-H	NEG
**M0924**	japonicumin D	6.083417	$C_{13}H_{24}O_3$	273.1706747	255.1245, 255.1596, 273.0008, 273.0151, 273.0347, 273.0405, 273.0492, 273.0764, 273.1365, 273.1703	M+FA-H	NEG
**M0925**	Lucidumoside D	6.083417	$C_{27}H_{36}O_{13}$	567.2079782		M-H	NEG
**M0926**	3'-Methoxydaidzein	6.103717	$C_{16}H_{12}O_5$	329.066424	285.0405, 285.0761, 285.1119, 299.0908, 311.0195, 311.0554, 311.0910, 328.1830, 328.2207, 329.0663	M+FA-H	NEG
**M0927**	6-hydroxymusizin,8-o-beta-d-glucoside	6.103717	$C_{20}H_{24}O_9$	407.1346093	297.0399, 305.1449, 327.0877, 339.0489, 345.1329, 361.2974, 363.0869, 407.0366, 407.1283, 407.1335	M-H, $_2$M-H	NEG
**M0928**	eupalinilide a	6.103717	$C_{19}H_{23}ClO_7$	443.1114707	254.9050, 300.9936, 305.1512, 317.1957, 317.8529, 343.2106, 395.5538, 407.1346, 442.2455, 443.1157	M+FA-H	NEG
**M0929**	Norsantal	6.145033	$C_{15}H_{10}O_6$	285.0402616	243.0299, 243.1584, 244.9821, 257.0454, 264.9908, 266.9890, 267.0296, 270.0171, 270.0504, 285.0402	M-H, $_2$M-H	NEG
**M0930**	Isolappaol C	6.165517	$C_{30}H_{34}O_{10}$	553.2078507		M-H	NEG

**M0931**	4-methoxy-3H-1,2-benzodioxole	6.18585	$C_8H_8O_3$	151.039973	131.0346, 132.1121, 133.0507, 133.3581, 135.0470, 136.0164, 149.0451, 150.9217, 151.0037, 151.0399	M-H	NEG
**M0932**	5,7,4'-Trihydroxy-8-methoxyflavanone	6.206317	$C_{16}H_{14}O_6$	283.0610451		M-H_2O-H	NEG
**M0933**	14-deoxyandrographolide-19-β-D-glucoside	6.206317	$C_{26}H_{40}O_9$	495.2600407	314.3069, 315.1948, 327.1129, 332.1943, 333.2067, 335.7633, 432.0996, 449.2385, 495.1783, 495.2597	M-H	NEG
**M0934**	Oroxylin A-7-o-beta-D-glucuronide	6.2262	$C_{22}H_{20}O_{11}$	459.0932004		M-H	NEG
**M0935**	3,4,3',4'-Tetrahydroxy-2-methoxychalcone	6.251067	$C_{16}H_{14}O_6$	303.085549		M+H	POS
**M0936**	Chrysophanol 8-O-EC-D-glucoside	6.2663	$C_{21}H_{20}O_9$	461.1088306	297.0399, 299.0549, 313.0567, 401.0869, 415.1010, 415.2342, 461.0768, 461.0924, 461.1030, 461.1123	M+FA-H	NEG
**M0937**	Methyl-(2-hydoxy-3-ethoxy-benzyl)ether	6.30825	$C_{10}H_{14}O_3$	363.1816748		$_2$M-H	NEG
**M0938**	eupatoroxin	6.30825	$C_{20}H_{24}O_8$	373.1291062		M-H_2O-H	NEG
**M0939**	gibberellin 7	6.30825	$C_{19}H_{22}O_5$	375.1446556	331.5284, 341.3901, 342.1104, 345.1321, 357.1336, 367.2549, 372.5617, 374.2561, 374.2613, 375.1443	M+FA-H	NEG
**M0940**	hildecarpin	6.348833	$C_{17}H_{14}O_7$	329.0663889		M-H	NEG
**M0941**	4-hydroxy-1-naphthalenyl-β-d-glucopyranoside	6.348833	$C_{20}H_{28}O_7$	361.165707	305.1462, 306.1394, 313.5297, 315.1608, 317.1382, 317.1757, 325.2007, 328.1308, 343.1563, 361.1660	M-H_2O-H	NEG
**M0942**	adenostemmoside b	6.348833	$C_{26}H_{38}O_{10}$	509.23891	379.0814, 386.6647, 442.1874, 448.6342, 463.1572, 463.2284, 463.2907, 465.2175, 477.2724, 509.2386	M-H	NEG
**M0943**	vaccaxanthone	6.371333	$C_{16}H_{12}O_8$	313.0352784	271.0853, 283.0251, 292.5750, 295.0254, 298.0126, 298.0493, 312.1911, 312.4754, 312.7202, 313.0351	M-H_2O-H	NEG
**M0944**	Denchrysan A	6.39175	$C_{14}H_{10}O_5$	257.0455031		M-H	NEG

**M0945**	Gomphrenol	6.393783	$C_{16}H_{10}O_7$	315.0491188	269.0444, 269.0730, 283.0597, 283.0919, 285.0374, 293.1139, 297.0386, 297.1822, 300.0620, 315.0490	M+H	POS
**M0946**	2-(methoxymethyl)-2,4,5-trimethyl-1,3-dioxolane	6.4124	$C_8H_{16}O_3$	159.1026313		M-H	NEG
**M0947**	eupalinilide j	6.455633	$C_{22}H_{28}O_{10}$	475.1559431		M+Na	POS
**M0948**	5-hydroxy-p-menth-6-en-2-one	6.514983	$C_{10}H_{16}O_2$	213.1132796	208.8463, 209.3698, 209.3878, 210.2938, 210.3109, 212.9966, 213.0148, 213.0549, 213.0762, 213.1129	M+FA-H	NEG
**M0949**	3-methyl-7-methylideneoct-2-ene-1,8-diol	6.537917	$C_{10}H_{18}O_2$	169.1234274	140.9257, 144.5293, 148.9864, 149.0018, 151.7993, 168.9904, 169.0127, 169.0500, 169.0872, 169.1231	M-H	NEG
**M0950**	andrographidine A	6.536933	$C_{23}H_{26}O_{10}$	463.1587753	305.2479, 312.7208, 320.2428, 323.1119, 338.2539, 406.1245, 427.1383, 445.0870, 445.1479, 463.0881	M+H	POS
**M0951**	Lappaol E	6.536933	$C_{30}H_{34}O_{10}$	577.2037178		M+Na	POS
**M0952**	Acbglu	6.536933	$C_{30}H_{26}O_{12}$	579.1482928	415.5928, 456.1218, 482.0979, 517.1420, 520.6483, 525.1191, 543.1276, 561.1378, 578.3313, 579.1406	M+H, M+Na, M+H-H_2O	POS
**M0953**	1,6,7-trihydroxy-3-methoxy-9,10-anthraquinone	6.558333	$C_{15}H_{10}O_6$	267.0298736	227.0348, 238.0640, 238.9969, 239.0717, 241.0503, 247.0004, 249.1478, 252.0433, 266.6063, 267.0305	M-H_2O-H	NEG
**M0954**	Irilin B	6.558333	$C_{16}H_{12}O_6$	281.0454376		M-H_2O-H	NEG
**M0955**	(3S,5R,6S,7E)-5,6-epoxy-3-hydroxy-7-megaslig-men-9-one	6.61905	$C_{13}H_{20}O_3$	207.1376656	171.1172, 174.1030, 175.0383, 179.0700, 189.0538, 189.1270, 189.1634, 207.0647, 207.1035, 207.1376	M+H-H_2O	POS
**M0956**	magnolone	6.621467	$C_{21}H_{22}O_7$	385.1293943		M-H	NEG
**M0957**	nagilactone e	6.63925	$C_{19}H_{24}O_6$	371.1479403		M+Na	POS
**M0958**	Pseudoginsenoside FII	6.726317	$C_{42}H_{72}O_{14}$	845.4914548	515.2813, 637.4305, 727.8778, 769.4600, 799.3207, 799.4853, 845.1663, 845.3881, 845.4826, 845.5076	M-H, M+FA-H	NEG

**M0959**	graminones B	6.740983	$C_{21}H_{22}O_8$	385.1271097		M+H-H_2O	POS
**M0960**	omphalin	6.761283	$C_{16}H_{17}NO_2$	256.1326049		M+H	POS
**M0961**	EUPALINILIDE C	6.761283	$C_{20}H_{24}O_7$	399.1426846	173.3217, 177.0544, 209.0957, 211.1922, 237.0902, 341.1169, 359.7268, 381.1343, 398.6842, 399.1308	M+Na	POS
**M0962**	5-METHYLFURFURAL	6.767467	$C_6H_6O_2$	219.0661816		$_2$M-H	NEG
**M0963**	7,2'-Dihydroxy-4'methoxyisoflavanon	6.767467	$C_{16}H_{14}O_5$	285.0765966		M-H	NEG
**M0964**	hallactone a	6.767467	$C_{19}H_{22}O_6$	391.1396528	121.3594, 222.0510, 297.0401, 345.1669, 347.1090, 347.1374, 347.1507, 358.1010, 373.1293, 391.1387	M+FA-H	NEG
**M0965**	alnustinol	6.782733	$C_{16}H_{14}O_6$	303.0853349	273.5987, 277.3994, 281.5477, 285.1486, 285.2220, 287.1621, 288.0609, 289.0548, 303.0483, 303.0851	M+H	POS
**M0966**	3-hydroxydehydroiso-alpha-lapachone	6.78805	$C_{15}H_{12}O_4$	301.0713047	258.0519, 259.0555, 260.9776, 268.0359, 280.9865, 286.0479, 287.0513, 300.9958, 301.0344, 301.0710	M+FA-H	NEG
**M0967**	tomentogenin	6.821717	$C_{21}H_{36}O_5$	369.2624306	259.2047, 269.1688, 277.2159, 295.2265, 337.1053, 351.1120, 351.2538, 369.1303, 369.2215, 369.2642	M+H-H_2O, M+H	POS
**M0968**	Lilac alcohol	6.82825	$C_{10}H_{18}O_2$	215.1286693	214.3332, 214.9476, 214.9944, 215.0136, 215.0343, 215.0718, 215.0780, 215.0917, 215.1018, 215.1286	M+FA-H	NEG
**M0969**	desacetyleupaserrin	6.82825	$C_{20}H_{26}O_6$	361.1653929	284.0334, 284.1072, 299.0554, 299.1297, 317.1391, 325.2020, 328.1301, 343.1529, 361.0605, 361.1655	M-H	NEG
**M0970**	Gibberellin A119	6.82825	$C_{19}H_{24}O_5$	377.1602706	333.1334, 341.1964, 345.1345, 352.8743, 359.1498, 359.1954, 373.3055, 373.8165, 376.7362, 377.1598	M+FA-H	NEG
**M0971**	[4]-Gingerdiol	6.842083	$C_{15}H_{24}O_4$	269.174149	246.0461, 246.5480, 248.5502, 251.1635, 255.0651, 257.5537, 258.0558, 269.0803, 269.1364, 269.1749	M+H	POS

**M0972**	(1r,5s,6s,7s,10r)-1β,6α-dihydroxyeudesman-4-one	6.869917	$C_{14}H_{24}O_3$	285.1704349		M+FA-H	NEG
**M0973**	7,6'-dihydroxy-3'-methoxyisoflavone	6.884233	$C_{16}H_{12}O_5$	285.0750123	261.0500, 261.5534, 262.0148, 264.0558, 267.0652, 267.1945, 270.0524, 273.0610, 284.2964, 285.0750	M+H	POS
**M0974**	Lucidin omega-methyl ether	6.911017	$C_{16}H_{12}O_5$	283.0608333	255.0648, 255.5522, 262.9946, 265.0495, 265.1448, 268.0375, 280.8896, 283.0041, 283.0246, 283.0605	M-H	NEG
**M0975**	Taurodeoxycholate;Deoxycholyltaurine	6.93025	$C_{26}H_{45}NO_6S$	498.289631	396.6441, 403.4004, 409.1938, 411.2284, 426.0822, 463.6728, 491.0608, 493.2166, 495.4885, 498.2886	M-H	NEG
**M0976**	notoginsenosider2	6.972433	$C_{41}H_{70}O_{13}$	815.4810093	463.4552, 475.3788, 619.4213, 637.4327, 703.2526, 725.4792, 743.1418, 769.4745, 814.8936, 815.4845	M-H, M+FA-H	NEG
**M0977**	asiaticoside F	7.013533	$C_{48}H_{78}O_{18}$	987.5186299	218.0681, 223.2571, 231.5604, 514.2851, 633.3854, 643.5057, 719.6413, 795.4587, 941.5115, 987.5146	M+FA-H	NEG
**M0978**	3-ethylpenta-2,4-dien-1-ol	7.036	$C_7H_{12}O$	157.0868771		M+FA-H	NEG
**M0979**	2,7-Dihydroxy-1-methylphenanthrene-5-carbaldehyde	7.066533	$C_{16}H_{12}O_3$	270.1119102		$M+NH_4$	POS
**M0980**	Hypnone	7.086783	C_8H_8O	103.0545837		$M+H-H_2O$	POS
**M0981**	PICRASINOSIDE B	7.096283	$C_{28}H_{40}O_{11}$	551.2498942	373.1617, 384.9862, 385.0387, 388.1894, 389.1968, 394.2134, 505.3295, 505.3387, 524.6494, 551.2501	M-H	NEG
**M0982**	germacranolide	7.127317	$C_{15}H_{22}O_4$	289.1403535	127.0389, 131.0499, 135.0811, 145.1015, 180.1090, 220.9345, 253.1606, 271.1702, 289.0919, 289.1415	M+Na	POS
**M0983**	oxyphyllenodiol a	7.18005	$C_{14}H_{22}O_3$	283.1548673		M+FA-H	NEG

**M0984**	hydrocotyloside ii	7.18005	$C_{48}H_{76}O_{19}$	955.4917789	795.4482, 835.4478, 837.3914, 859.3759, 942.0771, 942.5886, 948.0595, 953.4838, 955.1286, 955.4918	M-H	NEG
**M0985**	Flavidin	7.200783	$C_{15}H_{12}O_3$	285.0764688		M+FA-H	NEG
**M0986**	aromadendrin-5,7-dimethyl ether	7.242017	$C_{17}H_{16}O_6$	315.087229		M-H	NEG
**M0987**	5-methyoxymarmesin	7.262483	$C_{15}H_{16}O_5$	257.0818829		M-H_2O-H	NEG
**M0988**	2-(3-hydroxy-2-pent-2-enylcyclopentyl)acetic acid	7.352883	$C_{12}H_{20}O_3$	195.1377018	159.1167, 163.0387, 171.9587, 172.9775, 177.0537, 177.0911, 177.1271, 195.0795, 195.1012, 195.1373	M+H-H_2O, M+H	POS
**M0989**	pseudolaricacid f	7.352883	$C_{22}H_{26}O_7$	385.1657959	331.1369, 349.1456, 349.1942, 351.2158, 367.1046, 367.1559, 367.2071, 367.2460, 385.1583, 385.1657	M+H-H_2O	POS
**M0990**	Dipotassium glycyrrhizinate	7.352883	$C_{42}H_{60}O_{16}$-2	821.3940909	433.3073, 451.3194, 469.3305, 487.3374, 503.5635, 645.3587, 661.6494, 747.7203, 801.1570, 821.4153	M+NH_4, M+Na, M+H	POS
**M0991**	Protoaescigenin	7.373533	$C_{30}H_{50}O_6$	507.3673473		M+H	POS
**M0992**	Visammiol	7.427967	$C_{15}H_{16}O_5$	257.0817523		M-H_2O-H	NEG
**M0993**	cadinanetriol	7.427967	$C_{15}H_{28}O_3$	301.201817		M+FA-H	NEG
**M0994**	5α-hydroxytriptonide	7.449117	$C_{20}H_{22}O_7$	373.1289536	332.9927, 340.0937, 353.0018, 355.1178, 355.2300, 358.1046, 372.2416, 373.0104, 373.0179, 373.1269	M-H	NEG
**M0995**	Azelaone	7.46935	$C_8H_{14}O$	125.0971262		M-H	NEG
**M0996**	Cyclooct-2-enone	7.46935	$C_8H_{12}O$	169.0869227	148.9853, 149.0006, 155.9646, 166.7068, 166.9370, 168.6572, 168.9901, 169.0137, 169.0511, 169.0868	M+FA-H	NEG
**M0997**	1'-Acetoxyeugenol	7.47465	$C_{14}H_{16}O_5$	247.0959401	197.0604, 198.0673, 201.0905, 201.1266, 205.0856, 208.1159, 214.0622, 229.0855, 229.1945, 247.0963	M+H-H_2O	POS
**M0998**	(2R)- pterosin B 14- O- β-(4′- p-coumaroyl)- glucopyranoside	7.46935	$C_{29}H_{34}O_9$	525.2137343	293.0406, 321.8648, 327.1069, 449.1445, 457.2264, 479.0867, 479.1529, 525.0132, 525.0933, 525.2142	M-H	NEG

**M0999**	9H-Xanthene-2-carboxylic acid	7.4895	$C_{14}H_{10}O_3$	271.0611264	210.9992, 227.0713, 230.9858, 231.0067, 239.6037, 250.9907, 253.0508, 270.9790, 271.0031, 271.0616	M+FA-H	NEG
**M1000**	6-Hydroxycleroda-3,13-dien-16,15-olid-18-oic acid	7.4895	$C_{20}H_{28}O_5$	393.1914525	307.1928, 311.1656, 325.2022, 329.1746, 347.1867, 357.3762, 393.0123, 393.0186, 393.0998, 393.1919	M+FA-H	NEG
**M1001**	6,8-dihydroxy-3-phenyl-3,4-dihydroisochromen-1-one	7.510117	$C_{15}H_{12}O_4$	255.0662031	214.9937, 221.9336, 222.7032, 235.0000, 237.1527, 239.0289, 253.2091, 254.9853, 255.0154, 255.0658	M-H	NEG
**M1002**	Isotrifoliol	7.5936	$C_{16}H_{10}O_6$	297.0401959	256.9821, 260.1069, 276.9901, 279.1600, 282.0518, 282.8006, 296.6169, 296.7493, 296.9981, 297.0401	M-H	NEG
**M1003**	Angelol G	7.5936	$C_{20}H_{24}O_7$	375.1448439	293.0443, 293.2106, 307.1883, 310.0303, 311.1608, 314.4479, 329.1707, 331.1164, 331.1559, 375.1415	M-H	NEG
**M1004**	taxezopidin b	7.6346	$C_{26}H_{38}O_{10}$	491.228675	145.5928, 208.0942, 283.0581, 285.1856, 305.1479, 329.1751, 363.2501, 405.2627, 423.2694, 491.2247	M-H_2O-H	NEG
**M1005**	5,8-Dihydroxy-6,7-dimethoxyflavone	7.675683	$C_{17}H_{14}O_6$	295.0609929		M-H_2O-H	NEG
**M1006**	Gancaonin V	7.6984	$C_{19}H_{20}O_4$	357.1342349		M+FA-H	NEG
**M1007**	Methoxyacetic acid, 1-cyclopentylethyl ester	7.6984	$C_{10}H_{18}O_3$	371.2436469		$_2$M-H	NEG
**M1008**	Euponin	7.739467	$C_{20}H_{24}O_6$	341.1390881		M-H_2O-H	NEG
**M1009**	Anhydrocinnzeylanine	7.739467	$C_{22}H_{32}O_7$	453.2111566		M+FA-H	NEG
**M1010**	Butylbutyryllactate	7.759583	$C_{11}H_{20}O_4$	215.128759		M-H	NEG
**M1011**	Toralactone	7.759583	$C_{15}H_{12}O_5$	253.0504175	238.9632, 251.2125, 251.2806, 251.7274, 252.0362, 252.6446, 252.7879, 252.8191, 253.0023, 253.0503	M-H_2O-H	NEG
**M1012**	4',5-Dihydroxy-7-methoxyflavanone	7.759583	$C_{16}H_{14}O_5$	267.0660575	239.0712, 246.9999, 249.1491, 251.0345, 252.0424, 263.5100, 266.7784, 266.9846, 267.0355, 267.0659	M-H_2O-H, M-H	NEG

**M1013**	6-Methyl-7-(3-oxobutyl)bicyclo[4.1.0]heptan-3-one	7.7802	$C_{12}H_{18}O_2$	239.1288002	221.0807, 223.0281, 238.8686, 238.8945, 238.9923, 239.0229, 239.0335, 239.0702, 239.0993, 239.1287	M+FA-H	NEG
**M1014**	Pallidiflorin	7.78705	$C_{16}H_{12}O_4$	269.0802038	226.8660, 233.1531, 233.1892, 237.0524, 251.1635, 251.1741, 251.1997, 253.0508, 254.0561, 269.0801	M+H, M+Na	POS
**M1015**	Heliangin	7.8213	$C_{20}H_{26}O_6$	361.1656454	305.1474, 317.1750, 320.9967, 325.2012, 328.1329, 340.9858, 340.9918, 343.1547, 361.0025, 361.1656	M-H	NEG
**M1016**	tuberoside a	7.8213	$C_{45}H_{74}O_{18}$	883.4703807	837.4658, 843.3800, 845.3781, 851.3526, 851.8599, 859.8550, 860.3565, 865.3641, 865.8557, 883.4825	M-H_2O-H	NEG
**M1017**	alpha-Glycyrrhizin	7.863683	$C_{42}H_{62}O_{16}$	821.3973934	351.1078, 469.3322, 514.0925, 583.3646, 627.3548, 645.3669, 759.3928, 820.0195, 821.1075, 821.3970	M-H	NEG
**M1018**	schizonol	7.924983	$C_{10}H_{16}O_2$	213.1130873	193.0080, 195.1032, 207.3193, 209.1459, 212.9971, 213.0176, 213.0380, 213.0561, 213.0766, 213.1127	M+FA-H, $_2$M-H	NEG
**M1019**	Pachybasic acid	7.924983	$C_{15}H_8O_5$	313.0350988	269.0452, 269.4699, 272.9963, 273.5590, 292.9868, 305.1883, 305.1965, 312.2262, 312.9945, 313.0358	M+FA-H	NEG
**M1020**	Rubilactone	7.960217	$C_{15}H_{10}O_5$	251.0347649	223.0976, 224.2022, 224.8577, 225.0555, 230.3100, 230.9852, 231.0053, 250.1454, 250.9940, 251.0347	M-H_2O-H	NEG
**M1021**	9,10,11-trihydroxyoctadeca-12,15-dienoic acid	8.004567	$C_{18}H_{32}O_5$	327.2174414	291.1967, 306.9804, 306.9868, 307.0005, 309.2068, 326.9835, 326.9895, 327.1599, 327.1702, 327.2173	M-H_2O-H, M-H	NEG
**M1022**	denbinobin	8.049967	$C_{16}H_{12}O_5$	285.0750344	262.1411, 264.1423, 264.6434, 267.0646, 270.0511, 273.1479, 273.6492, 284.2930, 284.6563, 285.0749	M+H	POS

**M1023**	LIGNAN DERIV	8.069283	$C_{21}H_{16}O_7$	381.0958257	335.0905, 337.1092, 348.0612, 350.0782, 352.2934, 363.0853, 363.2164, 363.2990, 366.0727, 381.0955	M+H	POS
**M1024**	4-methyl-1-prop-1-en-2-ylcyclohex-3-en-1-ol	8.136217	$C_{10}H_{16}O$	197.118268		M+FA-H	NEG
**M1025**	(z)-6,7-epoxy-6,7-dihydroligustilide	8.136217	$C_{12}H_{14}O_3$	251.0921536		M+FA-H	NEG
**M1026**	Licoricesaponin D3	8.156817	$C_{50}H_{76}O_{21}$	1011.480028		M-H	NEG
**M1027**	Moracin B	8.17715	$C_{16}H_{14}O_5$	267.0664265		M-H_2O-H	NEG
**M1028**	Blumenol C	8.194233	$C_{13}H_{22}O_2$	228.1954422	151.1484, 155.1059, 165.1270, 165.1635, 175.1477, 193.1583, 210.1848, 211.1681, 228.1223, 228.1980	M+H-H_2O, M+NH_4, M+H	POS
**M1029**	p-(1-Propenyl)anisole	8.240317	$C_{10}H_{12}O$	193.0869147	149.0611, 150.0018, 152.9970, 161.0455, 173.0019, 178.0270, 192.9469, 192.9906, 193.0098, 193.0867	M+FA-H	NEG
**M1030**	Ugaxanthone	8.240317	$C_{18}H_{16}O_6$	373.0927064	332.9938, 342.2064, 343.0454, 343.0902, 343.1172, 352.9920, 352.9995, 358.0687, 373.0045, 373.0875	M+FA-H	NEG
**M1031**	5,4'-Dihidroxy-7,8,2',3'-tetramethoxyflavone	8.27545	$C_{19}H_{18}O_8$	375.1063516	347.0615, 352.0654, 352.0926, 352.1108, 357.2023, 359.0744, 360.0836, 370.0768, 370.2016, 375.1063	M+H	POS
**M1032**	Sandosaponin A	8.3043	$C_{48}H_{76}O_{19}$	955.4915743	339.0929, 415.5976, 435.1172, 497.1140, 510.9405, 537.6741, 661.6746, 727.6923, 858.1999, 955.4921	M-H	NEG
**M1033**	kosamol v	8.3249	$C_{21}H_{22}O_6$	369.134251		M-H	NEG
**M1034**	Penduletin	8.336617	$C_{18}H_{16}O_7$	345.0959567	305.2028, 312.0622, 315.0495, 316.0649, 327.2322, 328.1577, 329.0686, 330.0724, 343.4962, 345.0958	M+H, M+Na	POS
**M1035**	28-o-β-d-glucopyranosyl-2α-3β-dihydroxy-olean-12-ene-24,28-dioicacid	8.336617	$C_{36}H_{56}O_{11}$	647.3778604	311.2344, 317.2093, 357.2413, 369.2424, 389.3165, 407.3292, 435.3246, 453.3351, 471.3456, 647.3791	M+H-H_2O	POS

**M1036**	Uralsaponin B	8.34545	$C_{42}H_{62}O_{16}$	821.3977047	583.3579, 617.3962, 627.3492, 645.3610, 761.4472, 777.4414, 779.4493, 779.4638, 821.1076, 821.3972	M-H	NEG
**M1037**	lemmatoxin	8.419967	$C_{48}H_{78}O_{18}$	943.5248584		M+H, M+Na	POS
**M1038**	(S)-Columbianetin acetate	8.50175	$C_{16}H_{16}O_5$	271.0958964	210.0216, 211.1476, 217.1933, 235.2022, 240.2315, 253.0496, 254.2472, 270.2776, 271.0591, 271.0951	M+H-H_2O	POS
**M1039**	Theaspirone	8.511483	$C_{13}H_{20}O_2$	253.1444966	210.0314, 211.1871, 212.9971, 225.0552, 232.9832, 238.0269, 252.9885, 253.0502, 253.1124, 253.1444	M+FA-H	NEG
**M1040**	cubebinone	8.511483	$C_{23}H_{26}O_8$	475.1608769		M+FA-H	NEG
**M1041**	Saikosaponin A	8.543683	$C_{42}H_{68}O_{13}$	803.4538371	641.4043, 691.3821, 691.5192, 713.3217, 739.3932, 757.4125, 772.4229, 772.4426, 773.4432, 803.4545	M+H, M+NH_4, M+Na, M+H-H_2O	POS
**M1042**	Helminthosporin	8.554917	$C_{15}H_{10}O_5$	251.035001		M-H_2O-H	NEG
**M1043**	Benzenepropanal	8.584317	$C_9H_{10}O$	152.1068651		M+NH_4	POS
**M1044**	Wighteone	8.618983	$C_{20}H_{18}O_5$	337.1080514	268.8683, 268.9877, 269.0467, 276.9901, 293.2110, 296.9882, 296.9971, 316.9859, 336.9909, 337.1083	M-H	NEG
**M1045**	abrisaponin	8.680767	$C_{48}H_{78}O_{18}$	987.5183592	383.2509, 422.0099, 425.7875, 537.2418, 617.3996, 779.4465, 941.5117, 941.7267, 987.5037, 987.5297	M+FA-H	NEG
**M1046**	8-NONENOIC ACID	8.70385	$C_9H_{16}O_2$	311.2225982	293.2108, 296.0708, 310.9865, 310.9950, 311.0133, 311.0516, 311.0568, 311.0897, 311.1848, 311.2212	$_2$M-H	NEG
**M1047**	Oleanane-2H, +2O, 1COOH, O-HexA-HexA	8.769783	$C_{42}H_{64}O_{16}$	825.4245541	397.3095, 409.3449, 437.3403, 453.3346, 454.3386, 455.3507, 613.3679, 631.3795, 649.3914, 825.4277	M+H, M+Na	POS
**M1048**	4,4'-Bianisole	8.78865	$C_{14}H_{14}O_2$	259.0974562	216.0798, 218.9862, 229.0493, 230.9852, 238.9939, 243.0664, 244.0737, 258.9748, 259.0024, 259.0972	M+FA-H	NEG

**M1049**	Araboglycyrrhizin	8.809267	$C_{41}H_{62}O_{14}$	777.407657	339.8692, 469.3311, 541.6809, 583.3666, 585.3832, 615.8832, 627.3511, 700.6491, 715.4025, 777.4085	M-H	NEG
**M1050**	4-methoxy-5-hydroxybisabola-2,10-diene-9-one	8.82985	$C_{16}H_{26}O_3$	311.1862672	305.1422, 305.1482, 310.6346, 310.8706, 310.9892, 310.9960, 311.0104, 311.0418, 311.0566, 311.1864	M+FA-H	NEG
**M1051**	euphoheliosnoid a	8.8513	$C_{29}H_{36}O_8$	495.2393167		M+H-H_2O	POS
**M1052**	citrusinol	8.90935	$C_{20}H_{16}O_6$	353.1013906		M+H	POS
**M1053**	7beta-Hydroxypregn-4-ene-3,16-dione	8.97095	$C_{21}H_{30}O_3$	313.2154687		M+H-H_2O	POS
**M1054**	Gingerenone B	9.011483	$C_{22}H_{26}O_6$	409.1610314	189.8647, 208.0814, 208.5423, 230.6670, 242.1986, 305.2524, 355.2628, 373.2743, 408.3302, 409.1616	M+NH_4, M+Na, M+H	POS
**M1055**	23-o-acetyl-3β,12β,23s,24r-tetrahydroxy-20s,25-epoxydammarane 3-o-[β-d-xylopyranos-yl(1→2)]-β-d-xylopyranoside	9.070933	$C_{42}H_{70}O_{14}$	797.4704228	511.1758, 525.9866, 619.4130, 635.0840, 698.6719, 699.2874, 724.6384, 751.4639, 797.4552, 797.4747	M-H	NEG
**M1056**	Tuberoside D	9.0916	$C_{45}H_{74}O_{17}$	867.4758516	727.8149, 761.4491, 779.2927, 779.4589, 795.2978, 821.4705, 821.6581, 823.4832, 867.2920, 867.4747	M-H_2O-H	NEG
**M1057**	Glycyrrhisoflavanone	9.136967	$C_{21}H_{20}O_6$	369.1323937	327.1204, 328.1577, 341.1352, 341.1413, 351.1208, 351.1607, 351.2479, 351.2570, 359.2475, 369.1318	M+H	POS
**M1058**	isocarneagenin	9.136967	$C_{27}H_{44}O_5$	431.3147118		M+H-H_2O	POS
**M1059**	Cudraflavanone B	9.155033	$C_{20}H_{20}O_6$	355.1186068	314.9845, 314.9900, 315.0009, 317.8729, 334.9876, 334.9929, 339.2244, 345.2570, 355.0002, 355.1182	M-H	NEG
**M1060**	Dihydrooroxylin	9.175717	$C_{16}H_{14}O_5$	267.0661407	239.0714, 246.9998, 248.9984, 252.0426, 252.4347, 253.8906, 266.9834, 266.9888, 267.0304, 267.0659	M-H_2O-H	NEG

**M1061**	SNXDCBQJYXHIML-UHFFFAOYSA-N	9.175717	$C_{30}H_{48}O_6$	549.3436384		M+FA-H	NEG
**M1062**	chromolaevanedione	9.216983	$C_{14}H_{22}O_2$	267.1601472		M+FA-H	NEG
**M1063**	Chrysanthediol	9.216983	$C_{15}H_{24}O_2$	281.1758541		M+FA-H	NEG
**M1064**	18- hydroxy-7-oxo-15,16-epoxy friedolabda-5,13(16), 14- triene	9.219017	$C_{20}H_{28}O_3$	317.2100727	257.1512, 257.1873, 259.1674, 271.2054, 275.1988, 281.1893, 299.2000, 317.1331, 317.1706, 317.2095	M+H-H_2O, M+H	POS
**M1065**	leoheterin	9.219017	$C_{20}H_{30}O_4$	357.2021997		M+Na	POS
**M1066**	Isopregomisin	9.219017	$C_{22}H_{30}O_6$	391.2102562	271.1678, 295.1661, 313.1768, 331.1898, 337.2509, 355.1849, 355.2627, 373.2728, 391.1124, 391.2069	M+H	POS
**M1067**	Auxin b	9.237517	$C_{18}H_{30}O_4$	309.2070996	268.9831, 273.1837, 288.9890, 291.1963, 308.6334, 308.9983, 309.0384, 309.1208, 309.1693, 309.2067	M-H	NEG
**M1068**	15,16-bisnor-13-oxo-8(17)-labden-19-oicacid	9.259867	$C_{18}H_{28}O_3$	293.2103239	221.1528, 223.1329, 229.1952, 239.1780, 247.2046, 257.1888, 275.1996, 293.1115, 293.1715, 293.2097	M+H	POS
**M1069**	Isodihydrofutoquinol B	9.258117	$C_{21}H_{24}O_5$	355.1550326	294.9788, 309.2046, 311.2225, 314.9861, 323.1267, 334.9901, 337.2018, 354.9975, 355.1148, 355.1540	M-H, M+FA-H	NEG
**M1070**	noranthrone	9.3018	$C_{20}H_{20}O_6$	337.1081682		M-H_2O-H	NEG
**M1071**	Gerberinol	9.3236	$C_{21}H_{16}O_6$	387.0827706	209.7325, 211.0621, 216.1770, 229.0871, 289.6693, 302.2641, 305.2499, 369.2426, 370.5474, 387.0835	M+Na, M+K, M+H	POS
**M1072**	Isoneorautenol	9.365767	$C_{20}H_{18}O_4$	321.1131078		M-H	NEG
**M1073**	Gancaonin N	9.366833	$C_{21}H_{20}O_6$	391.1141586		M+Na	POS
**M1074**	VALERICANHYDRIDE	9.407033	$C_{10}H_{18}O_3$	371.2438088		$_2$M-H	NEG
**M1075**	senecioylplenolin	9.44855	$C_{20}H_{26}O_5$	369.1667324		M+Na	POS
**M1076**	Triptonoditerpenic acid	9.447883	$C_{21}H_{28}O_4$	389.1968893	345.1693, 348.9909, 348.9968, 353.1001, 368.9962, 383.1618, 388.0076, 388.9904, 389.0060, 389.1969	M+FA-H	NEG
**M1077**	chikusetsusaponin III	9.447883	$C_{47}H_{80}O_{17}$	961.5383227		M+FA-H	NEG

**M1078**	3-teracrylmelazolide b	9.46905	$C_{18}H_{26}O_4$	329.1716874		M+H-H_2O, M+Na	POS
**M1079**	Licoarylcoumarin	9.46905	$C_{21}H_{20}O_6$	369.1324417	319.0952, 320.1035, 321.1119, 322.1181, 323.1231, 333.1102, 333.2031, 351.1213, 351.2083, 369.1341	M+H	POS
**M1080**	subprogenin B	9.489183	$C_{30}H_{48}O_5$	533.3482928	465.2389, 465.3199, 487.3439, 512.9966, 513.0063, 513.0181, 526.7635, 533.0136, 533.0220, 533.3081	M-H, M+FA-H	NEG
**M1081**	6-isopropenyl-4,4a-dimethyl-1,2,3,4,4a,5,6,7-octahydro-naphthalen-1-ol	9.532467	$C_{15}H_{24}O$	203.1792221	161.0956, 161.1328, 175.1479, 184.0445, 185.1330, 203.0510, 203.0691, 203.1063, 203.1445, 203.1791	M+H-H_2O	POS
**M1082**	teucladiol	9.553117	$C_{15}H_{26}O_2$	283.1913545		M+FA-H	NEG
**M1083**	Pichtosin	9.61505	$C_{12}H_{20}O_2$	195.1390318		M-H	NEG
**M1084**	2-[(1R)-4-methyl-1-cyclohex-3-enyl]prop-2-enyl acetate	9.61505	$C_{12}H_{18}O_2$	239.1289072	195.1389, 198.9808, 213.3350, 218.9867, 221.1176, 223.0275, 238.9932, 239.0340, 239.0648, 239.1281	M+FA-H	NEG
**M1085**	(3R,5S)-3,5-dimethyloctan-4-one	9.654367	$C_{10}H_{20}O$	201.1496626	155.1436, 180.9914, 181.0080, 183.1018, 183.1176, 200.9975, 201.0171, 201.0771, 201.1139, 201.1491	M+FA-H	NEG
**M1086**	licoricesaponin c2	9.696717	$C_{42}H_{62}O_{15}$	805.4029182	214.7718, 225.0376, 242.8234, 249.4147, 319.7002, 349.1531, 351.0586, 464.8885, 677.5095, 805.4035	M-H	NEG
**M1087**	Glycyrin	9.738317	$C_{22}H_{22}O_6$	383.1479177	297.0396, 297.0751, 299.0909, 311.0529, 312.0617, 320.1949, 327.0854, 365.3200, 367.5547, 383.1445	M+H, M+Na	POS
**M1088**	Cimigoside	9.7787	$C_{35}H_{56}O_9$	643.3802911		M+Na	POS
**M1089**	Vaccaroside	9.7787	$C_{36}H_{54}O_{10}$	647.3778841	357.2419, 369.2394, 383.2599, 389.3224, 407.3308, 435.3226, 453.3354, 471.3448, 647.2670, 647.3806	M+H, M+NH_4, M+Na	POS

**M1090**	Tifentai	9.799083	$C_{27}H_{28}N_2O_4$	467.1930401	305.2520, 309.0749, 316.0383, 354.6975, 376.3316, 395.2581, 407.1713, 442.8875, 465.4242, 467.1936	M+Na, M+K, M+H	POS
**M1091**	KJKNNQHQAHDKSZ-UHFFFAOYSA-N	9.839717	$C_{15}H_{24}O$	203.1791806	168.0656, 174.1408, 175.1479, 185.1342, 192.1668, 203.0518, 203.0698, 203.1067, 203.1457, 203.1791	M+H-H_2O	POS
**M1092**	hex-3-en-1-yl butanoate	9.843267	$C_{10}H_{18}O_2$	339.2538032		$_2$M-H	NEG
**M1093**	3β,5α,9α,14β-tetrahydroxy-(22e)-ergosta-7,22-dien-6-one	9.843267	$C_{28}H_{44}O_5$	505.3172773		M+FA-H	NEG
**M1094**	1,2,5,5-Tetramethyl-1,3-cyclopentadiene	9.86075	C_9H_{14}	123.1169503		M+H	POS
**M1095**	caesaldekarin e	9.86075	$C_{24}H_{30}O_6$	437.1923247	365.3181, 366.3221, 383.3289, 384.3315, 401.3216, 407.3300, 419.3304, 436.3803, 437.0996, 437.1924	M+Na, M+K, M+NH_4, M+H	POS
**M1096**	Ginsenoside Rg3	9.86075	$C_{42}H_{72}O_{13}$	785.5031277		M+H	POS
**M1097**	1,5-di-isobutyl-3,3-dimethyl[3,1,0]-cyclohexadione	9.9073	$C_{16}H_{26}O_2$	295.1912248	266.9859, 274.9936, 277.1793, 294.9832, 294.9989, 295.0058, 295.0198, 295.0585, 295.1368, 295.1902	M+FA-H	NEG
**M1098**	9(S)-HOT	9.9448	$C_{18}H_{30}O_3$	295.2260293	175.1482, 179.1429, 189.1127, 231.2098, 241.1943, 249.2224, 259.2049, 277.2152, 295.1647, 295.2246	M+H	POS
**M1099**	Acetytastragaloside	9.9482	$C_{47}H_{74}O_{17}$	909.4869441	761.4401, 779.4503, 803.4521, 821.4685, 863.4781, 863.6796, 876.8296, 894.3227, 909.3011, 909.4846	M-H	NEG
**M1100**	6-Methylquinizarin	9.989367	$C_{15}H_{10}O_4$	253.0505865	215.9925, 216.2215, 225.0561, 232.9829, 234.9976, 238.0265, 243.4203, 252.1697, 252.9880, 253.0504	M-H	NEG

The columns provide critical information for component confirmation:

Retention time (min): Refers to the time elapsed from sample injection to the moment when the concentration of the separated component reaches its maximum value at the column outlet; this parameter is critical for qualitative matching of compounds and evaluating separation efficiency.

Mass Error (ppm): Represents the relative deviation between the measured mass-to-charge ratio (m/z) of the ion and the m/z of the matched ion in the database. Generally, a smaller absolute value indicates higher accuracy of compound identification, with values typically required to be within ±5 ppm for reliable confirmation.

Formula: The molecular formula of the metabolite, which is derived from the theoretical m/z and elemental composition analysis, providing a foundational basis for structural elucidation.

Theoretical m/z: The calculated mass-to-charge ratio of the target ion (e.g., $[M+H]^+$, $[M-H]^-$) based on the metabolite’s molecular formula, used as a reference for comparing with the measured m/z.

m/z: The experimentally detected mass-to-charge ratio of the target ion in the MS spectrum, which is compared with the theoretical m/z to verify the consistency of the compound’s molecular weight.

Fragment Ions: The top 10 secondary fragment ions with a relative intensity of ≥5% in the MS/MS spectrum. These characteristic fragment peaks are generated by the cleavage of chemical bonds in the parent ion, and their mass-to-charge ratios and relative intensities are key for confirming the compound’s structure (e.g., distinguishing isomers or verifying functional groups).

Adducts: The form of adduct ions formed between the metabolite and matrix molecules (e.g., H^+, H_2O, FA^-) during ionization, such as M-H (deprotonated ion), M+H (protonated ion), or M+FA-H (formate adduct ion); this parameter helps determine the optimal ion mode for detecting the compound.

Ion mode: Specifies the mass spectrometry detection mode used for the compound: "POS" (Positive Ion Mode) for compounds that are easily protonated (e.g., alkaloids, terpenes), and "NEG" (Negative Ion Mode) for compounds that tend to lose protons (e.g., phenols, organic acids).

Table S2. List of the potential drug-like compounds of CS (OB: oral bioavailability, DL: drug-likeness).

NO.	Compounds	Formula	InChIKey	OB	DL
**CS1**	(+)-Balanophonin	$C_{20}H_{20}O_6$	GWCSSLSMGCFIFR-LNFBDUAVSA-N	54.74	0.4
**CS2**	(1R,3R)-3-[(E)-3-Methoxy-2-methyl-3-oxo-1-propenyl]-2,2-dimethylcyclopropanecarboxylic acid (S)-3-(2-butenyl)-2-methyl-4-oxo-2-cyclopenten-1-yl	$C_{21}H_{28}O_5$	SHCRDCOTRILILT-LGPFIRNVSA-N	62.52	0.31
**CS3**	(2R)-7-hydroxy-2-(4-hydroxyphenyl)chroman-4-one	$C_{15}H_{12}O_4$	FURUXTVZLHCCNA-CQSZACIVSA-N	71.12	0.18
**CS4**	2,7-Dihydroxy-1-methylphenanthrene-5-carbaldehyde	$C_{16}H_{12}O_3$	VTKYCHCADSWEBT-UHFFFAOYSA-N	79.55	0.21
**CS5**	3'-Hydroxymelanettin	$C_{16}H_{12}O_6$	IGHVYQBBZBLNDW-UHFFFAOYSA-N	30.69	0.27
**CS6**	3'-Methoxydaidzein	$C_{16}H_{12}O_5$	MUYAUELJBWQNDH-UHFFFAOYSA-N	48.57	0.24
**CS7**	4',5-Dihydroxy-7-methoxyflavanone	$C_{16}H_{14}O_5$	DJOJDHGQRNZXQQ-CQSZACIVSA-N	39.6	0.24
**CS8**	4',5-Dihydroxyflavone	$C_{15}H_{10}O_4$	OKRNDQLCMXUCGG-UHFFFAOYSA-N	48.55	0.19
**CS9**	5,4'-Dihidroxy-7,8,2',3'-tetramethoxyflavone	$C_{19}H_{18}O_8$	XJMLRDUBVCDIKJ-UHFFFAOYSA-N	77.13	0.45
**CS10**	5-Hydroxy-7,8,4'-trimethoxyflavone	$C_{18}H_{16}O_6$	RRZRJBICWWNHRB-UHFFFAOYSA-N	65.82	0.33
**CS11**	6-Hydroxyluteolin	$C_{15}H_{10}O_7$	VYAKIUWQLHRZGK-UHFFFAOYSA-N	46.93	0.28
**CS12**	Alisol C	$C_{30}H_{46}O_5$	DORJGGFFCMZTHW-KXVAGGRESA-N	32.7	0.82
**CS13**	Aloe emodin	$C_{15}H_{10}O_5$	YDQWDHRMZQUTBA-UHFFFAOYSA-N	83.38	0.24
**CS14**	Andropanolide	$C_{20}H_{30}O_5$	BOJKULTULYSRAS-QPSYGYIJSA-N	46.96	0.36
**CS15**	Angelol G	$C_{20}H_{24}O_7$	BAHUBXAYVOCLNA-FNYRBRLGSA-N	46.03	0.34
**CS16**	Aureusidin	$C_{15}H_{10}O_6$	WBEFUVAYFSOUEA-PQMHYQBVSA-N	53.42	0.24
**CS17**	Azaleatin	$C_{16}H_{12}O_7$	RJBAXROZAXAEEM-UHFFFAOYSA-N	54.28	0.3
**CS18**	Catechin	$C_{15}H_{14}O_6$	PFTAWBLQPZVEMU-DZGCQCFKSA-N	54.83	0.24
**CS19**	Cirsiliol	$C_{17}H_{14}O_7$	IMEYGBIXGJLUIS-UHFFFAOYSA-N	43.46	0.34
**CS20**	Cirsimaritin	$C_{17}H_{14}O_6$	ZIIAJIWLQUVGHB-UHFFFAOYSA-N	30.35	0.3

**CS21**	Cnidilin	$C_{17}H_{16}O_5$	NNDOCYLWULORAM-UHFFFAOYSA-N	32.69	0.28
**CS22**	Consume close grain	$C_{16}H_{14}O_6$	LHUSIUJRMSQNJO-CQSZACIVSA-N	68.12	0.27
**CS23**	cubebinone	$C_{23}H_{26}O_8$	BFDODNWEBMWJIS-JKSUJKDBSA-N	38.95	0.75
**CS24**	Dehydrocorydaline	$C_{22}H_{24}NO_4^+$	RFKQJTRWODZPHF-UHFFFAOYSA-N	41.98	0.68
**CS25**	Deoxyelephantopin	$C_{19}H_{20}O_6$	JMUOPRSXUVOHFE-GZZMZBIISA-N	105.32	0.4
**CS26**	Deoxypodophyllotoxin	$C_{22}H_{22}O_7$	ZGLXUQQMLLIKAN-SVIJTADQSA-N	37.75	0.83
**CS27**	Diphyllin	$C_{21}H_{16}O_7$	VMEJANRODATDOF-UHFFFAOYSA-N	36.23	0.75
**CS28**	Ellagic acid	$C_{14}H_6O_8$	AFSDNFLWKVMVRB-UHFFFAOYSA-N	43.06	0.43
**CS29**	Eriodictyol	$C_{15}H_{12}O_6$	SBHXYTNGIZCORC-ZDUSSCGKSA-N	71.79	0.24
**CS30**	Eupatorin	$C_{18}H_{16}O_7$	KLAOKWJLUQKWIF-UHFFFAOYSA-N	30.23	0.37
**CS31**	Flavidin	$C_{15}H_{12}O_3$	QMOLHJKSZMURCV-UHFFFAOYSA-N	30.1	0.26
**CS32**	Flazin	$C_{17}H_{12}N_2O_4$	USBWYUYKHHILLZ-UHFFFAOYSA-N	94.28	0.39
**CS33**	Galangin	$C_{15}H_{10}O_5$	VCCRNZQBSJXYJD-UHFFFAOYSA-N	45.55	0.21
**CS34**	gibberellin 7	$C_{19}H_{22}O_5$	SEEGHKWOBVVBTQ-NFMPGMCNSA-N	73.8	0.5
**CS35**	Gibberellin A119	$C_{19}H_{24}O_5$	JKQCGVABHHQYKQ-XZXFKYPMSA-N	76.36	0.49
**CS36**	Glyasperin C	$C_{21}H_{24}O_5$	RCZMWVKBVFOCEE-ZDUSSCGKSA-N	45.56	0.4
**CS37**	Glycyrin	$C_{22}H_{22}O_6$	FWWGXZYUURXJLK-UHFFFAOYSA-N	52.61	0.47
**CS38**	Gomphrenol	$C_{16}H_{10}O_7$	WBQNGHIOSSPSDW-UHFFFAOYSA-N	48.63	0.4
**CS39**	Hedysarimcoumestan B	$C_{16}H_{10}O_6$	AAKHRTZXSZBLFQ-UHFFFAOYSA-N	48.14	0.43
**CS40**	Herbacetin	$C_{15}H_{10}O_7$	ZDOTZEDNGNPOEW-UHFFFAOYSA-N	36.07	0.27
**CS41**	Hispidulin	$C_{16}H_{12}O_6$	IHFBPDAQLQOCBX-UHFFFAOYSA-N	30.97	0.27
**CS42**	Irisolidone	$C_{17}H_{14}O_6$	VOOFPOMXNLNEOF-UHFFFAOYSA-N	37.78	0.3
**CS43**	Iristectorigenin A	$C_{17}H_{14}O_7$	CCRPIWFQMLICCY-UHFFFAOYSA-N	63.36	0.34
**CS44**	Isoformononetin	$C_{16}H_{12}O_4$	LNIQZRIHAMVRJA-UHFFFAOYSA-N	38.37	0.21
**CS45**	Isolariciresinol	$C_{20}H_{24}O_6$	OGFXBIXJCWAUCH-KPHUOKFYSA-N	66.51	0.39
**CS46**	Isorhamnetin	$C_{16}H_{12}O_7$	IZQSVPBOUDKVDZ-UHFFFAOYSA-N	49.6	0.31

**CS47**	Isotrifoliol	$C_{16}H_{10}O_6$	OVLUQDOJWGTPHA-UHFFFAOYSA-N	31.94	0.42
**CS48**	Kaempferol	$C_{15}H_{10}O_6$	IYRMWMYZSQPJKC-UHFFFAOYSA-N	41.88	0.24
**CS49**	Leucocyanidin	$C_{15}H_{14}O_7$	SBZWTSHAFILOTE-SOUVJXGZSA-N	30.84	0.27
**CS50**	Licoarylcoumarin	$C_{21}H_{20}O_6$	LCRIQVFKVCYUAO-UHFFFAOYSA-N	59.62	0.43
**CS51**	Licochalcone A	$C_{21}H_{22}O_4$	KAZSKMJFUPEHHW-DHZHZOJOSA-N	40.79	0.29
**CS52**	Licochalcone B	$C_{16}H_{14}O_5$	DRDRYGIIYOPBBZ-XBXARRHUSA-N	76.76	0.19
**CS53**	Licoisoflavone A	$C_{20}H_{18}O_6$	KCUZCRLRQVRBBV-UHFFFAOYSA-N	41.61	0.42
**CS54**	Licoricone	$C_{22}H_{22}O_6$	GGWMNTNDTRKETA-UHFFFAOYSA-N	63.58	0.47
**CS55**	Liquiritigenin	$C_{15}H_{12}O_4$	FURUXTVZLHCCNA-AWEZNQCLSA-N	32.76	0.18
**CS56**	Lupiwighteone	$C_{20}H_{18}O_5$	YGCCASGFIOIXIN-UHFFFAOYSA-N	51.64	0.37
**CS57**	Medioresil	$C_{21}H_{24}O_7$	VJOBNGRIBLNUKN-BMHXQBNDSA-N	57.2	0.62
**CS58**	Methyl p-hydroxybenzoate glucoside	$C_{14}H_{18}O_8$	IZEJPIRXGMAQFL-YGEZULPYSA-N	38.31	0.22
**CS59**	Moracin B	$C_{16}H_{14}O_5$	GOUSNRMGQRTROZ-UHFFFAOYSA-N	55.85	0.23
**CS60**	Morin	$C_{15}H_{10}O_7$	YXOLAZRVSSWPPT-UHFFFAOYSA-N	46.23	0.27
**CS61**	Mosloflavone	$C_{17}H_{14}O_5$	SIVAITYPYQQYAP-UHFFFAOYSA-N	34.04	0.26
**CS62**	Negletein	$C_{16}H_{12}O_5$	ZTHLHHDJRXJGRX-UHFFFAOYSA-N	41.16	0.23
**CS63**	Neoglycyrol	$C_{21}H_{18}O_6$	LWESBHWAOZORCQ-UHFFFAOYSA-N	90.78	0.67
**CS64**	N-Feruloyltyramine	$C_{18}H_{19}NO_4$	NPNNKDMSXVRADT-WEVVVXLNSA-N	86.71	0.26
**CS65**	Nodakenetin	$C_{14}H_{14}O_4$	FWYSBEAFFPBAQU-GFCCVEGCSA-N	84.77	0.18
**CS66**	Oroxylin A	$C_{16}H_{12}O_5$	LKOJGSWUMISDOF-UHFFFAOYSA-N	41.37	0.23
**CS67**	Pachybasic acid	$C_{15}H_8O_5$	ICBHXKIMYNFEIB-UHFFFAOYSA-N	45.98	0.25
**CS68**	Pinocembrin	$C_{15}H_{12}O_4$	URFCJEUYXNAHFI-ZDUSSCGKSA-N	64.72	0.18
**CS69**	Quercetin	$C_{15}H_{10}O_7$	REFJWTPEDVJJIY-UHFFFAOYSA-N	46.43	0.28
**CS70**	Rhamnazin	$C_{17}H_{14}O_7$	MYMGKIQXYXSRIJ-UHFFFAOYSA-N	47.14	0.34
**CS71**	Rhein	$C_{15}H_8O_6$	FCDLCPWAQCPTKC-UHFFFAOYSA-N	47.07	0.28
**CS72**	Rutaretin	$C_{14}H_{14}O_5$	FVFQELHSZVFPDZ-VIFPVBQESA-N	70.1	0.2

**CS73**	Semilicoisoflavone B	$C_{20}H_{16}O_6$	LWZACZCRAUQSLH-UHFFFAOYSA-N	48.78	0.55
**CS74**	Sesamolin	$C_{20}H_{18}O_7$	ZZMNWJVJUKMZJY-AFHBHXEDSA-N	40.13	0.88
**CS75**	Skimmin	$C_{15}H_{16}O_8$	VPAOSFFTKWUGAD-TVKJYDDYSA-N	38.35	0.32
**CS76**	Syringetin	$C_{17}H_{14}O_8$	UZMAPBJVXOGOFT-UHFFFAOYSA-N	36.82	0.37
**CS77**	Aurantiamide Acetate	$C_{27}H_{28}N_2O_4$	VZPAURMDJZOGHU-DQEYMECFSA-N	58.02	0.52
**CS78**	TMC-58B	$C_{25}H_{26}N_2O_3$	KSVKECXWDNCRTM-GOTSBHOMSA-N	45.76	0.43
**CS79**	tomentogenin	$C_{21}H_{36}O_5$	VLJHJGYZWGQIKC-VBDNBMKMSA-N	37.84	0.53
**CS80**	Toralactone	$C_{15}H_{12}O_5$	WEHXAEGTVPWKDY-UHFFFAOYSA-N	46.46	0.24
**CS81**	Triptonide	$C_{20}H_{22}O_6$	SWOVVKGLGOOUKI-ZHGGVEMFSA-N	68.45	0.68
**CS82**	Wighteone	$C_{20}H_{18}O_5$	KIMDVVKVNNSHGZ-UHFFFAOYSA-N	42.8	0.36
**CS83**	Wogonin	$C_{16}H_{12}O_5$	XLTFNNCXVBYBSX-UHFFFAOYSA-N	30.68	0.23
**CS84**	Zedoarolide B	$C_{15}H_{22}O_5$	DXGIJGSOOPTGDC-LCLLMFOLSA-N	135.56	0.21

Table S3. Mean binding energies of CS drug-like compounds against selected breast-cancer-related proteins.

Values (kcal mol^{-1}) represent the average over nine independent docking poses (three technical replicates) for each compound–protein pair; poses were generated with AutoVina and ranked by cluster size. All means are provided in Table S3.

	Gene name	TP53	SRC	AKT1	PIK3R1	MAPK1	PIK3CA	STAT3	HSP90AA1	ESR1
	PDB ID	2IG0	2SRC	3CQW	2IUH	2OJG	4JPS	6NJS	1YET	3ERT
1	(+)-Balanophonin	–7.20	–8.36	–8.41	–6.41	–9.38	–8.08	–6.40	–7.88	–8.56
2	(1R,3R)–3–[(E)–3–Methoxy–2–methyl–3–oxo–1–propenyl]–2,2–dimethylcyclopropanecarboxylic acid (S)–3–(2–butenyl)–2–methyl–4–oxo–2–cyclopenten–1–yl	–7.76	–8.33	–8.21	–6.40	–8.88	–7.92	–6.34	–7.78	–8.16
3	(2R)–7–hydroxy–2–(4–hydroxyphenyl)chroman–4–one	–7.98	–9.27	–9.33	–6.96	–9.28	–8.77	–6.58	–7.98	–8.88
4	2,7–Dihydroxy–1–methylphenanthrene–5–carbaldehyde	–6.88	–9.30	–9.90	–6.88	–9.69	–8.64	–6.77	–7.97	–8.98
5	3'–Hydroxymelanettin	–7.67	–8.90	–8.57	–6.87	–9.19	–8.06	–6.80	–8.36	–8.79
6	3'–Methoxydaidzein	–6.01	–7.98	–7.86	–5.81	–8.39	–7.99	–5.66	–6.92	–6.68
7	4',5–Dihydroxy–7–methoxyflavanone	–7.73	–8.89	–8.99	–6.58	–9.28	–8.50	–6.68	–7.89	–8.73
8	4',5–Dihydroxyflavone	–5.79	–7.77	–7.60	–5.56	–8.34	–7.84	–5.72	–6.86	–7.34
9	5,4'–Dihidroxy–7,8,2',3'–tetramethoxyflavone	–5.44	–7.28	–7.32	–5.43	–7.73	–7.27	–5.42	–6.67	–6.16
10	5–Hydroxy–7,8,4'–trimethoxyflavone	–5.74	–7.42	–7.19	–5.34	–7.76	–7.40	–5.71	–6.54	–6.96
11	6–Hydroxyluteolin	–6.06	–8.04	–8.06	–5.69	–8.76	–7.73	–6.13	–7.37	–6.76
12	Alisol C	–5.33	–8.73	–4.66	–5.98	–7.10	–6.42	–6.26	–7.46	–8.08
13	Aloe emodin	–5.83	–7.91	–7.97	–5.71	–9.04	–7.76	–5.53	–6.88	–6.82
14	Andropanolide	–7.92	–10.12	–10.09	–7.46	–10.67	–9.38	–7.44	–8.79	–10.16
15	Angelol G	–7.02	–8.26	–7.65	–5.83	–7.93	–7.38	–6.10	–6.81	–8.22
16	Aureusidin	–5.96	–7.84	–7.91	–5.69	–8.63	–7.71	–6.02	–6.94	–6.96
17	Azaleatin	–5.91	–7.84	–7.43	–5.43	–8.60	–7.17	–5.73	–6.96	–6.73
18	Catechin	–6.01	–7.77	–7.43	–5.67	–8.61	–7.24	–5.82	–6.97	–6.79

19	Cirsiliol	–6.04	–7.72	–7.62	–5.42	–8.14	–7.58	–6.06	–6.63	–6.66
20	Cirsimaritin	–5.64	–7.80	–7.20	–5.40	–7.91	–7.32	–5.90	–6.59	–6.80
21	Cnidilin	–6.39	–7.43	–6.86	–5.38	–7.96	–7.34	–5.82	–6.51	–7.29
22	Consume close grain	–7.88	–8.90	–9.12	–6.79	–9.36	–8.63	–6.84	–8.08	–8.91
23	cubebinone	–5.37	–7.94	–8.07	–5.64	–8.38	–8.13	–6.37	–7.24	–7.14
24	Dehydrocorydaline	–5.60	–7.40	–4.85	–5.11	–8.28	–7.57	–5.51	–6.47	–6.53
25	Deoxyelephantopin	–5.98	–7.74	–6.66	–5.66	–7.70	–6.87	–6.19	–7.06	–8.00
26	Deoxypodophyllotoxin	–5.63	–7.63	–6.99	–5.57	–7.76	–7.36	–5.88	–7.28	–7.26
27	Diphyllin	–5.83	–8.84	–6.73	–6.12	–8.97	–7.89	–6.49	–6.93	–6.80
28	Ellagic acid	–5.71	–8.50	–7.72	–6.10	–9.36	–8.02	–6.22	–7.37	–6.74
29	Eriodictyol	–6.20	–8.13	–7.88	–5.62	–8.83	–7.76	–5.87	–7.27	–7.14
30	Eupatorin	–5.69	–7.67	–7.28	–5.21	–7.79	–7.44	–5.90	–6.46	–6.69
31	Flavidin	–5.51	–7.86	–7.70	–5.83	–8.86	–7.76	–5.52	–7.07	–7.47
32	Flazin	–6.20	–8.23	–7.99	–5.89	–8.94	–7.87	–6.01	–7.20	–7.26
33	Galangin	–5.86	–7.66	–7.81	–5.64	–8.32	–7.38	–5.59	–6.90	–7.02
34	gibberellin 7	–6.04	–7.50	–6.08	–5.81	–6.43	–6.16	–5.84	–7.01	–7.67
35	Gibberellin A119	–9.16	–11.97	–12.84	–8.74	–12.44	–11.35	–8.71	–10.52	–11.38
36	Glyasperin C	–6.19	–7.78	–7.22	–5.27	–8.03	–7.41	–5.88	–6.79	–6.80
37	Glycyrin	–6.31	–7.81	–7.07	–5.53	–7.93	–7.54	–6.13	–6.97	–7.07
38	Gomphrenol	–6.00	–8.07	–8.34	–6.00	–8.61	–7.58	–6.21	–7.11	–6.92
39	Hedysarimcoumestan B	–7.59	–9.58	–9.50	–7.03	–9.97	–8.94	–6.90	–8.39	–9.28
40	Herbacetin	–5.81	–7.96	–7.96	–5.54	–8.40	–7.28	–5.81	–6.92	–6.87
41	Hispidulin	–5.64	–7.79	–7.47	–5.43	–7.91	–7.72	–5.79	–6.67	–6.72
42	Irisolidone	–5.58	–7.37	–6.22	–5.53	–7.44	–7.12	–5.43	–6.39	–6.22
43	Iristectorigenin A	–6.08	–7.69	–7.76	–5.90	–8.40	–7.43	–5.57	–6.62	–6.53
44	Isoformononetin	–5.80	–7.83	–7.36	–5.41	–7.60	–7.81	–5.60	–6.44	–6.62
45	Isolariciresinol	–6.80	–8.68	–8.74	–6.39	–9.20	–7.62	–6.44	–8.02	–8.34
46	Isorhamnetin	–6.17	–8.18	–8.02	–5.74	–8.66	–7.61	–5.71	–7.23	–6.87
47	Isotrifoliol	–5.96	–7.97	–7.66	–5.77	–9.08	–8.08	–5.88	–7.32	–7.04
48	Kaempferol	–5.89	–7.78	–7.43	–5.52	–8.28	–7.23	–5.82	–6.82	–6.97
49	Leucocyanidin	–5.88	–7.50	–7.26	–5.77	–8.56	–6.96	–5.88	–6.93	–6.70
50	Licoarylcoumarin	–5.92	–7.63	–6.88	–5.61	–8.28	–7.28	–6.17	–6.89	–6.93
51	Licochalcone A	–6.09	–7.67	–7.77	–5.38	–8.13	–7.06	–6.03	–6.54	–7.68

52	Licochalcone B	–5.77	–7.48	–7.24	–5.31	–8.09	–7.03	–5.49	–6.61	–6.92
53	Licoisoflavone A	–6.62	–8.33	–7.52	–5.62	–8.23	–7.51	–6.08	–7.14	–7.30
54	Licoricone	–6.20	–7.81	–7.81	–5.47	–7.48	–6.81	–5.78	–6.86	–7.04
55	Liquiritigenin	–5.93	–7.87	–7.83	–5.49	–8.67	–7.54	–5.62	–6.90	–7.59
56	Lupiwighteone	–6.34	–8.29	–7.52	–5.62	–8.10	–8.34	–6.20	–6.97	–7.46
57	Medioresil	–6.97	–8.96	–9.21	–6.50	–9.56	–9.26	–6.78	–8.36	–8.44
58	Methyl p–hydroxybenzoate glucoside	–6.79	–7.32	–7.54	–5.87	–7.63	–7.02	–5.83	–7.07	–7.38
59	Moracin B	–6.38	–7.47	–7.74	–5.60	–8.01	–7.29	–5.39	–6.89	–6.60
60	Morin	–6.22	–7.72	–7.48	–5.59	–8.20	–7.40	–5.74	–6.90	–6.97
61	Mosloflavone	–6.07	–7.67	–7.56	–5.28	–7.86	–7.49	–5.84	–6.70	–6.82
62	Negletein	–6.09	–7.81	–7.80	–5.50	–8.14	–7.70	–5.92	–7.18	–6.88
63	Neoglycyrol	–6.33	–8.44	–7.27	–5.87	–8.73	–8.38	–6.28	–7.38	–7.02
64	N–Feruloyltyramine	–6.68	–7.90	–8.14	–5.41	–8.01	–7.61	–6.09	–6.73	–7.82
65	Nodakenetin	–7.58	–8.61	–8.92	–6.49	–8.82	–8.50	–6.47	–7.74	–8.63
66	Oroxylin A	–6.07	–7.80	–7.59	–5.24	–7.87	–7.57	–5.83	–6.72	–6.72
67	Pachybasic acid	–5.98	–8.16	–8.00	–5.88	–9.16	–8.21	–5.82	–7.01	–7.28
68	Pinocembrin	–5.73	–6.57	–6.83	–5.56	–6.65	–6.28	–5.98	–7.06	–6.62
69	Quercetin	–6.08	–8.07	–7.68	–5.80	–8.74	–7.37	–5.94	–7.27	–7.02
70	Rhamnazin	–5.91	–7.80	–7.61	–5.61	–8.13	–7.62	–5.66	–7.11	–6.70
71	Rhein	–5.89	–8.23	–8.14	–6.10	–9.28	–8.10	–5.96	–7.16	–6.73
72	Rutaretin	–7.44	–8.67	–8.82	–6.69	–8.84	–8.22	–6.62	–7.82	–8.69
73	Semilicoisoflavone B	–6.66	–9.19	–7.38	–6.27	–9.34	–8.71	–6.44	–7.18	–7.77
74	Sesamolin	–8.77	–10.30	–10.78	–7.13	–10.62	–9.84	–7.32	–8.97	–10.36
75	Skimmin	–7.52	–8.98	–8.59	–6.74	–8.81	–8.08	–6.66	–8.39	–8.62
76	Syringetin	–6.17	–7.77	–8.19	–5.77	–8.59	–7.91	–5.52	–7.01	–6.44
77	Aurantiamide Acetate	–9.60	–8.88	–8.17	–6.48	–9.93	–9.12	–6.87	–8.53	–10.19
78	TMC–58B	–6.73	–7.42	–7.82	–5.03	–8.61	–6.26	–5.22	–7.13	–8.44
79	tomentogenin	–8.46	–11.21	–10.98	–8.30	–11.73	–10.71	–8.33	–9.76	–10.83
80	Toralactone	–5.77	–7.91	–7.78	–5.53	–8.56	–7.61	–5.53	–6.71	–6.66
81	Triptonide	–5.91	–7.67	–7.34	–5.99	–7.59	–7.03	–6.14	–7.14	–7.73
82	Wighteone	–6.62	–8.28	–7.94	–5.71	–7.89	–7.34	–5.94	–7.16	–6.80

83	Wogonin	–5.87	–7.66	–7.40	–5.27	–8.51	–7.52	–5.81	–6.89	–6.99
84	Zedoarolide B	–6.92	–9.12	–9.77	–6.81	–9.28	–8.33	–6.54	–8.02	–8.48

Table S4. List of Mass Spectrometry Parameter Information.

This table details the key mass spectrometry parameters used in positive (POS) and negative (NEG) ion modes for UHPLC–Q–Exactive Orbitrap MS/MS analysis. Parameters include spray voltage, capillary temperature, auxiliary gas heater temperature, sheath gas flow rate, auxiliary gas flow rate, S–lens RF level, mass range, full MS resolution, MS/MS resolution, and collision energy (NCE/stepped NCE). These optimized parameters ensure high sensitivity, accuracy, and reproducibility for the identification and quantification of CS chemical constituents.

Parameters	POS	NEG
Spray Voltage (V)	3800	–3000
Capillary Temperature (°C)	320	320
Aux gas heater temperature (°C)	350	350
Sheath Gas Flow Rate (Arb)	35	35
Aux gas flow rate (Arb)	8	8
S–lens RF level	50	50
Mass range (m/z)	100–1500	100–1500
Full ms resolution	60000	60000
MS/MS resolution	15000	15000
NCE/stepped NCE	10，20，40	10，20，40

Table S5. MD stability summary across complexes

This table summarizes key molecular dynamics (MD) stability metrics for each protein–ligand complex over the analysis window (primarily 30–100 ns, unless otherwise specified). Reported descriptors include protein backbone RMSD, ligand RMSD, protein–ligand center‑of‑mass (COM) distance, protein radius of gyration (Rg), and the minimum protein–ligand heavy‑atom distance (mindist) where available. For each metric, mean ± SD, median, and the 5–95% interval are provided to characterize both central tendency and fluctuation range. These statistics are used to justify the selection of the "stable plateau" window for downstream interaction and energetics analyses; missing entries indicate that the corresponding raw trajectory‑derived output was unavailable (e.g., sesamolin mindist file not found) and therefore not included.

Complex	Metric	Mean ± SD	Median	5–95%	Window (ns)	n_frames
SRC–tomentogenin	Backbone RMSD (Å)	2.05 ± 0.19	2.04	—	30–100	—
SRC–tomentogenin	Ligand RMSD (Å, aligned)	7.88 ± 0.47	7.89	—	30–100	—
SRC–tomentogenin	Protein–ligand COM distance (Å)	16.76 ± 0.52	16.78	15.89–17.56	30–100	7001
SRC–tomentogenin	Radius of gyration Rg (nm)	2.465 ± 0.010	2.465	2.449–2.482	10–100	9001
SRC–tomentogenin	Min heavy–atom distance (Å)	1.86 ± 0.15	1.85	1.63–2.11	30–100	7001
SRC–sesamolin	Backbone RMSD (Å)	1.62 ± 0.18	1.59	1.36–1.96	30–100	7001
SRC–sesamolin	Protein–ligand COM distance (Å)	13.73 ± 0.35	13.72	13.18–14.32	30–100	7001
SRC–sesamolin	Radius of gyration Rg (nm)	2.455 ± 0.011	2.455	2.437–2.473	30–100	7001
SRC–sesamolin	Min heavy–atom distance (Å)	—	—	—	30–100	—
TP53–sesamolin	Min heavy–atom distance (Å), median	2.54	—	—	30–100	—
TP53–sesamolin	Frames with min dist >10 Å (%)	9.7	—	—	30–100	—

TP53–sesamolin	Max min heavy–atom distance (Å)	22.42, 42.75 ns	—	—	30–100	—
TP53–sesamolin	Backbone RMSD (Å)	4.21 ± 1.43	4.26	—	30–100	—
TP53–sesamolin	Radius of gyration Rg (nm)	1.500 ± 0.044	1.492	1.441–1.586	30–100	7001

Table S6. Quality control (QC) summary statistics for MD production runs.

This table reports the quality control (QC) statistics derived from the MD engine energy/output files to verify stable thermodynamic conditions under the applied ensemble. Summary descriptors (mean, SD, minimum, and maximum) are provided for temperature, pressure, density, and selected energy terms (e.g., potential energy, when available), calculated over the production trajectory (or the stated QC window). These QC metrics demonstrate that the simulations remained near the target conditions (e.g., temperature and density stability under NPT), supporting the reliability of the subsequent structural and interaction analyses. Full‑frame time series (when needed) are provided as Supplementary Data, while this table retains only compact, reviewer‑friendly summary statistics.

System	Metric	Mean	SD	Min	Max	Window (ns)
SRC–tomentogenin	Temperature	300.002	1.242	294.806	304.993	0–100
SRC–tomentogenin	Pressure	0.416	132.28	–496.421	514.468	0–100
SRC–tomentogenin	Density	1024.304	3.665	1010.375	1037.673	0–100
SRC–tomentogenin	Potential	–98,3958.808	1235.195	–988,538.312	–979,179.438	0–100
SRC–tomentogenin	Total Energy	–797,960.984	1549.346	–803,726.5	–792,237.25	0–100
SRC–sesamolin	Temperature	300.018	1.319	295.049	304.756	0–100
SRC–sesamolin	Pressure	1.673	139.342	–545.828	559.798	0–100
SRC–sesamolin	Density	1021.288	3.922	1006.579	1037.624	0–100
SRC–sesamolin	Potential	–864,307.79	1195.851	–868,591.875	–860,186.75	0–100
SRC–sesamolin	Total Energy	–691,905.067	1507.386	–697,581.812	–685,454.25	0–100
SRC–sesamolin	Temperature	300.01	1.313	295.049	304.756	30–100
SRC–sesamolin	Pressure	1.041	138.526	–545.828	539.898	30–100
SRC–sesamolin	Density	1021.253	3.93	1006.579	1036.062	30–100
SRC–sesamolin	Potential	–864,332.401	1202.542	–868,591.875	–860,186.75	30–100
SRC–sesamolin	Total Energy	–691,934.311	1512.345	–697,581.812	–686,048.625	30–100

Table S7. Top flexible residues from Cα RMSF analysis (SRC‑Tomentogenin, 30‑100 ns).

This table lists the top 10 most mobile residues based on Cα root‑mean‑square fluctuation (RMSF) computed over the selected stable trajectory segment (30–100 ns). For each residue, residue index/identity (and chain labeling if applicable) and RMSF magnitude are reported to highlight flexible regions that may contribute to binding‑site adaptability or global conformational dynamics. The table provides a concise complement to structural stability metrics by localizing motion to specific sequence positions, enabling comparison with contact/hydrogen‑bond hotspots and facilitating interpretation of binding‑site plasticity.

Rank	Chain	Residue	RMSF (Å)
1	A	MET82	3.134
2	A	SER209	2.967
3	A	GLY210	2.869
4	A	ALA194	2.852
5	A	ASP208	2.761
6	A	ASN193	2.714
7	A	VAL83	2.557
8	A	THR114	2.51
9	A	LYS195	2.506
10	A	LEU533	2.395

Table S8. Top protein‑ligand contact residues and occupancies (SRC‑Tomentogenin, 30‑100 ns).

This table ranks the most persistent protein–ligand contact residues identified within the stable analysis window (30–100 ns) using the stated geometric contact cutoff (e.g., heavy‑atom distance criterion as used in your contact analysis). For each ranked residue, contact occupancy (%) and the corresponding number of contact frames are reported relative to the total analyzed frames, providing a quantitative map of the dominant binding‑pocket interactions. This contact‑frequency summary supports the main mechanistic interpretation that stable binding is maintained by a core set of high‑occupancy pocket residues, while lower‑occupancy contacts reflect transient or peripheral interactions. The full residue‑by‑residue contact table is retained as Supplementary Data for completeness.

Rank	Residue	Contact occupancy (%)	Contact frames	Total frames
1	PHE324	100	183	183
2	LEU244	91.3	167	183
3	LEU326	91.3	167	183
4	MET233	90.7	166	183
5	TYR301	88	161	183
6	GLU229	87.4	160	183
7	ASP323	86.9	159	183
8	ILE255	78.1	143	183
9	GLY325	71	130	183
10	PHE226	68.9	126	183
11	LEU329	63.9	117	183
12	VAL232	63.9	117	183
13	ARG328	54.6	100	183
14	THR257	48.6	89	183
15	VAL242	43.7	80	183
16	LYS214	42.6	78	183
17	ALA230	37.2	68	183
18	ALA225	30.1	55	183
19	LEU216	25.1	46	183
20	LEU236	24.6	45	183

Table S9. Hydrogen‑bond statistics and key H‑bonding residues (SRC‑Tomentogenin, 30‑100 ns).

This table compiles hydrogen bond (H‑bond) statistics over the stable trajectory segment (30–100 ns) using the geometric criterion d ⩽ 3.5 Å and donor–H–acceptor angle ⩾ 150° (analysis stride as specified in the dataset). It includes (i) residue‑level H‑bond occupancy (%) to identify key H‑bonding residues, (ii) donor→acceptor pair occupancies for the most frequent protein–ligand H‑bond pairs, and (iii) a compact distribution of the H‑bond count state (0/1/2/⩾3) to summarize how often the complex is supported by none versus multiple H‑bonds. Together, these outputs distinguish persistent anchoring H‑bonds from intermittent events and provide a reviewer‑friendly quantitative basis for statements about whether binding is primarily H‑bond–driven or dominated by nonpolar/shape‑complementarity contacts.

Item	Value	Notes
Window	30–100 ns	n = 7001 frames
H–bonds per frame (mean ± SD)	0.983 ± 0.528	
Frames with 0 H–bonds (%)	13.9	
Frames with 1 H–bonds (%)	74.7	
Frames with 2 H–bonds (%)	10.6	
Frames with ≥3 H–bonds (%)	0.8	
Top H–bonding residues (occupancy %)		Residue–level occupancy
GLU229	71	Residue occupancy (%)
ASP323	26.1	Residue occupancy (%)
LYS214	8.7	Residue occupancy (%)
THR257	5.8	Residue occupancy (%)
Top H–bond pairs (occupancy %)		Donor–acceptor pairs; 30–100 ns
LIG453:O5 → GLU229:O	70.3	Ligand→Protein
LIG453:O4 → ASP323:OD1	14.5	Ligand→Protein
LIG453:O2 → ASP323:OD1	14.5	Ligand→Protein
LYS214:NZ → LIG453:O4	8	Protein→Ligand
LIG453:O4 → ASP323:OD2	8	Ligand→Protein
LIG453:O2 → ASP323:OD2	8	Ligand→Protein
THR257:OG1 → LIG453:O1	5.8	Protein→Ligand
LIG453:O5 → ALA225:O	1.4	Ligand→Protein

LIG453:O4 → VAL242:O	1.4	Ligand→Protein
LYS214:NZ → LIG453:O3	0.7	Protein→Ligand
LIG453:O5 → GLU229:OE2	0.7	Ligand→Protein

Table S10. MM/(P)BSA binding free energy decomposition with block‑SEM (SRC‑Tomentogenin, 30‑100 ns).

This table presents the MM/(P)BSA binding free energy decomposition computed over the stable window (30–100 ns), reported separately under GBSA and PBSA models. Component terms include van der Waals (ΔVDW), electrostatics (ΔEEL), polar solvation (ΔSOLV), nonpolar solvation/surface term (ΔSURF), and the resulting total binding energy (ΔTOTAL). Values are reported as mean with uncertainty estimated by block‑averaged SEM (block‑SEM), enabling assessment of both the dominant energetic contributions and their robustness to trajectory fluctuations. This decomposition supports mechanistic interpretation by indicating whether binding is driven primarily by dispersion/packing (ΔVDW), electrostatics (ΔEEL), or offset by solvation penalties (ΔSOLV), and it provides the numeric basis underlying the corresponding main/supplementary figures.

Energy term	GBSA (mean ± SEM, kJ/mol)	PBSA (mean ± SEM, kJ/mol)
ΔVDW	–50.728 ± 0.313	–50.711 ± 0.321
ΔEEL	–12.165 ± 0.456	–12.095 ± 0.492
ΔSOLV	27.602 ± 0.523	38.276 ± 0.599
ΔSURF	–6.373 ± 0.031	–4.144 ± 0.010
ΔTOTAL	–41.665 ± 0.541	–28.674 ± 0.545

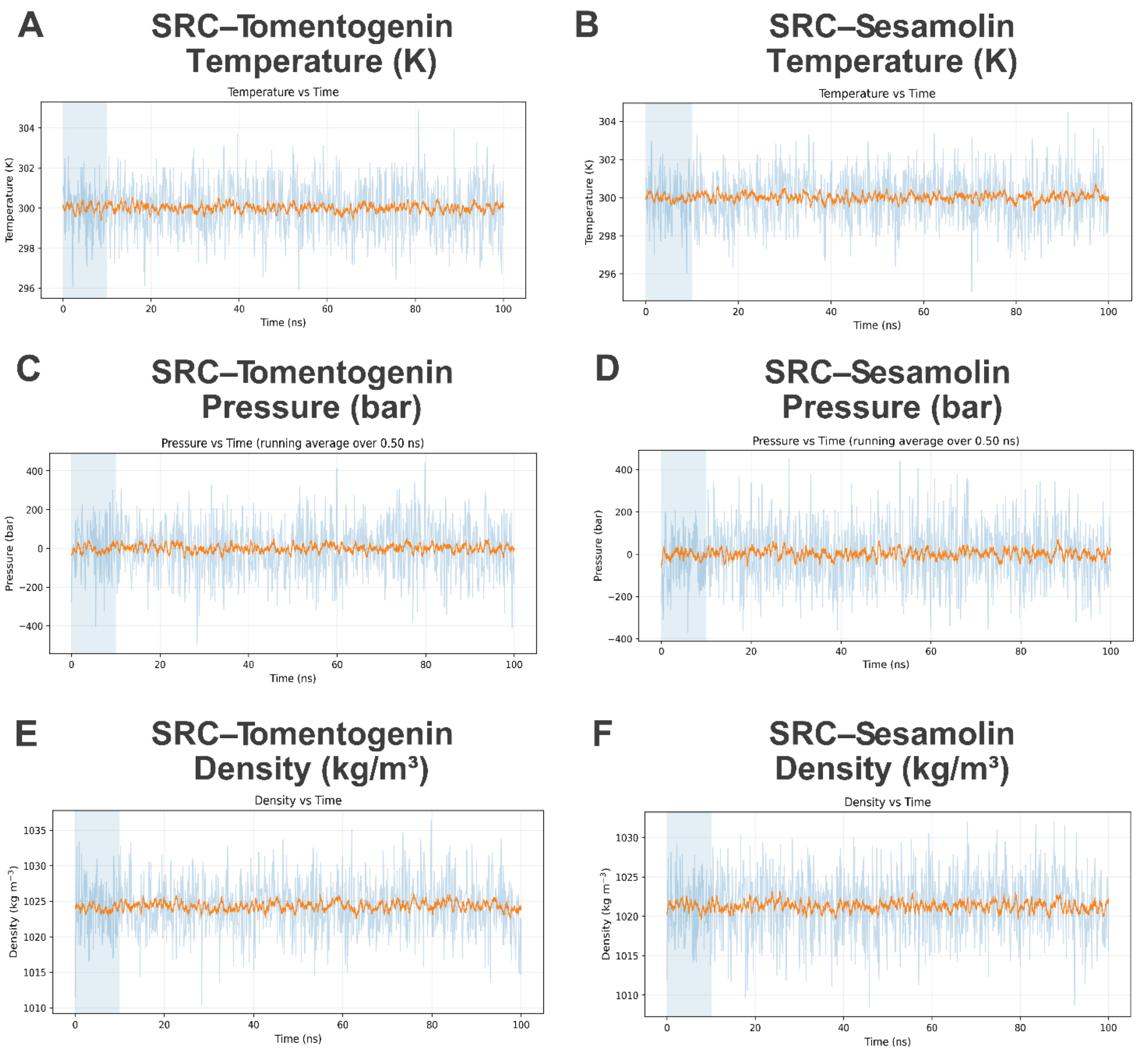

Figure S1. Quality－control (QC) profiles for MD simulations of the SRC–Tomentogenin and SRC–Sesamolin complexes. Time evolution of temperature (**A**,**B**), pressure (**C**,**D**), and density (**E**,**F**) during the 100 ns production MD runs. The shaded region indicates the initial equilibration period (first 10 ns), which was excluded from quantitative interpretation. Temperature remained stably controlled around the target value (~300 K), and density converged to a stable level consistent with well－equilibrated NPT conditions. Instantaneous pressure exhibited large fluctuations, as expected for atomistic simulations, but showed no systematic drift over time. Together, these QC profiles support the thermodynamic stability and proper equilibration of the simulated systems.

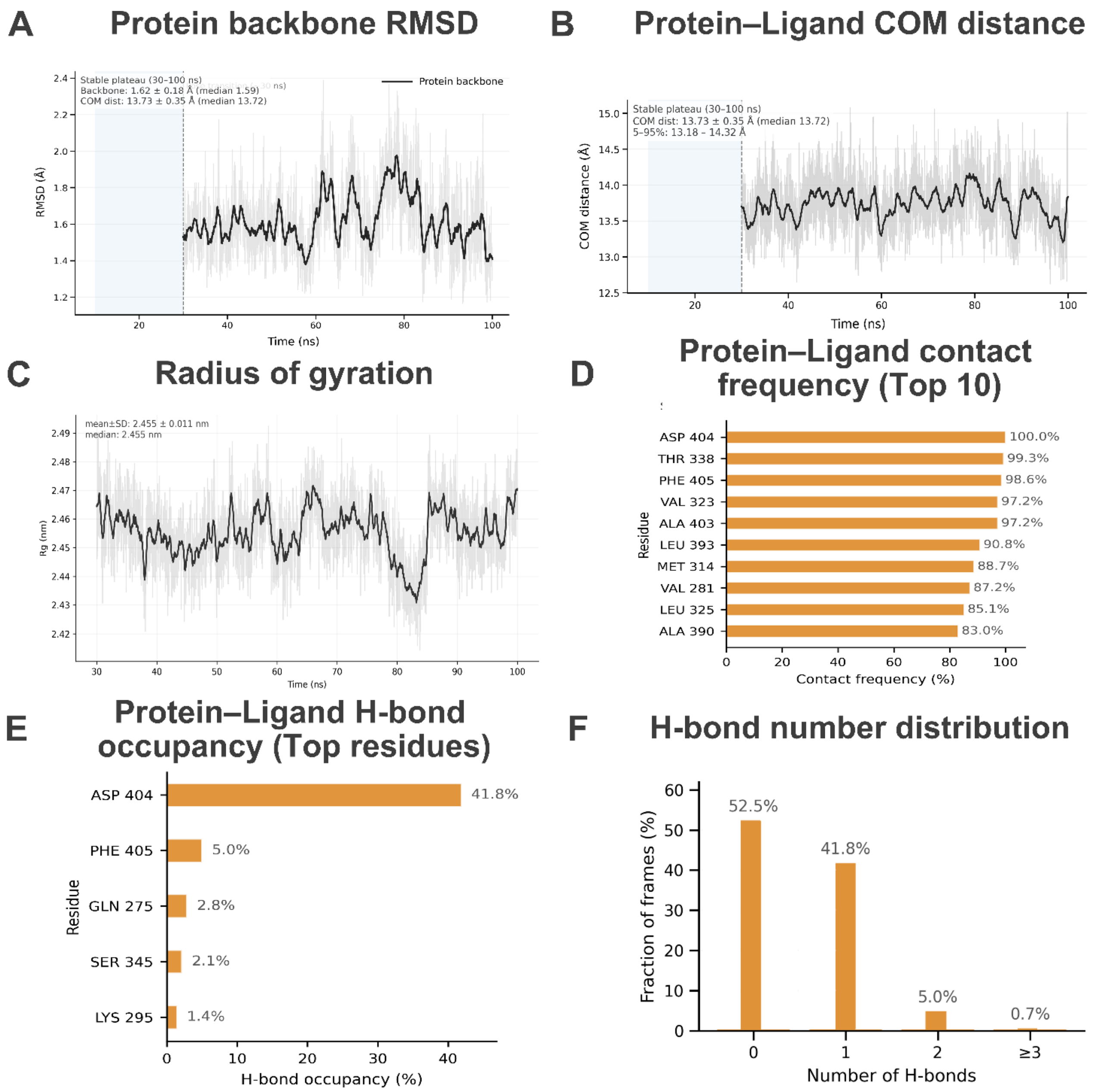

Figure S2. Structural stability and interaction profile of the SRC–Sesamolin complex during MD (30–100 ns). (**A**) Protein backbone RMSD calculated after fitting to the protein backbone, showing a stable plateau over 30–100 ns. (**B**) Protein–ligand center–of–mass (COM) distance over 30–100 ns, indicating a compact binding geometry with limited fluctuations. (**C**) Radius of gyration (Rg) of the protein, reflecting overall compactness during the production window. (**D**) Top residue–wise protein–ligand contact frequencies (distance cutoff < 4.0 Å) summarizing persistent pocket contacts. (**E**) Top protein→ligand hydrogen–bond occupancies computed using a geometric definition (donor–acceptor distance ⩽ 3.5 Å and angle ⩾ 150°) over 30–100 ns. (**F**) Distribution of the number of protein–ligand hydrogen bonds per frame over 30–100 ns, highlighting the typical H–bonding state during binding.

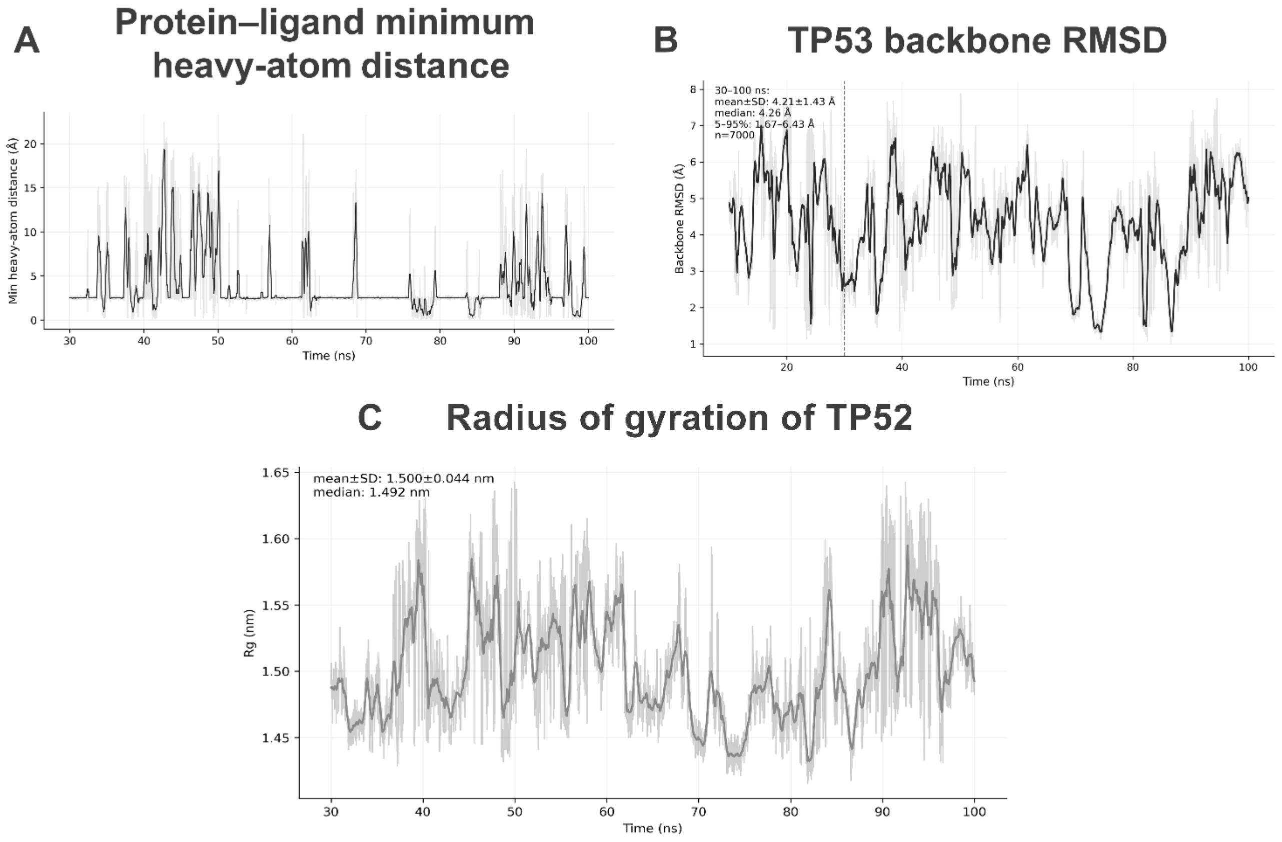

Figure S3. TP53 ‑ Sesamolin shows insufficient MD stability (negative/inconclusive). (A) Protein ‑ ligand minimum heavy ‑ atom distance over 30–100 ns. The distance distribution indicates intermittent ligand departure events (median 2.54 Å; 9.70% frames >10 Å; maximum 22.42 Å at 42.75 ns). (B) TP53 backbone RMSD time series (shown for 10–100 ns; statistics computed for 30–100 ns): mean ± SD 4.21±1.43 Å; 5–95% 1.67–6.43 Å. (C) TP53 radius of gyration (Rg) over 30–100 ns: mean ± SD 1.500±0.044 nm; median 1.492 nm; 5–95% 1.441–1.586 nm (n = 7001 frames).

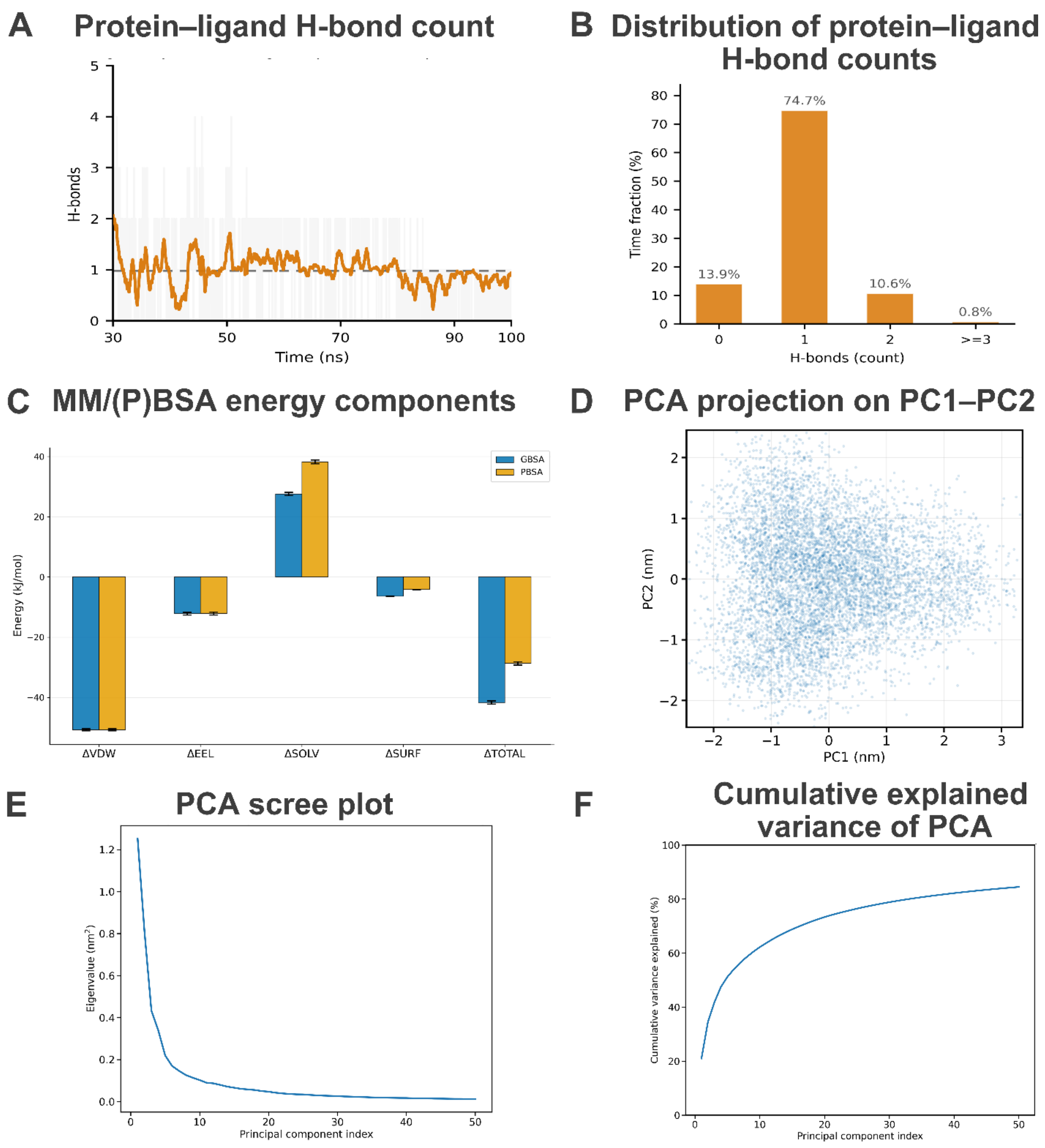

Figure S4. Additional interaction and PCA diagnostics for SRC－Tomentogenin (30–100 ns). (**A**) Time series of the number of protein－ligand hydrogen bonds within the stable window (30–100 ns) using the geometric criterion d ⩽ 3.5 Å and angle ⩾ 150° (analysis stride as indicated in the panel). (**B**) Distribution of hydrogen－bond counts over 30–100 ns, summarizing the occupancy of typical H－bonding states. (**C**) MM/(P)BSA energy decomposition averaged over 30–100 ns (bars show mean; error bars represent block－averaged SEM), reporting van der Waals, electrostatic, solvation, surface, and total binding energy terms. (**D**) Scatter projection of the trajectory onto PC1－PC2 derived from Cα coordinates (30–100 ns; sampling interval as indicated), illustrating the sampled conformational space. (**E**) PCA scree plot showing the variance explained by each principal

component. (**F**) Cumulative explained variance, indicating the fraction of total motion captured by the leading PCs.
